# Decoding the Molecular Effects of Atovaquone Linked Resistant Mutations on *Plasmodium falciparum* Cytb-ISP Complex in the Phospholipid Bilayer Membrane

**DOI:** 10.3390/ijms22042138

**Published:** 2021-02-21

**Authors:** Lorna Chebon-Bore, Taremekedzwa Allan Sanyanga, Colleen Varaidzo Manyumwa, Afrah Khairallah, Özlem Tastan Bishop

**Affiliations:** Research Unit in Bioinformatics (RUBi), Department of Biochemistry and Microbiology, Rhodes University, Makhanda 6140, South Africa; lornajemosop@gmail.com (L.C.-B.); asanyanga@gmail.com (T.A.S.); colleen.manyumwa06@gmail.com (C.V.M.); afrahkhairalla@gmail.com (A.K.)

**Keywords:** *Plasmodium falciparum* cytochrome bc_1_ complex, MD simulations, ATQ resistance, POPC:POPE phospholipid bilayer, heme and [2FE-2S] (rieske) cluster cofactors, dynamic residue network analysis, MD-TASK, MDM-TASK-web

## Abstract

Atovaquone (ATQ) is a drug used to prevent and treat malaria that functions by targeting the *Plasmodium falciparum* cytochrome b (PfCytb) protein. PfCytb catalyzes the transmembrane electron transfer (ET) pathway which maintains the mitochondrial membrane potential. The ubiquinol substrate binding site of the protein has heme bL, heme bH and iron-sulphur [2FE-2S] cluster cofactors that act as redox centers to aid in ET. Recent studies investigating ATQ resistance mechanisms have shown that point mutations of PfCytb confer resistance. Thus, understanding the resistance mechanisms at the molecular level via computational approaches incorporating phospholipid bilayer would help in the design of new efficacious drugs that are also capable of bypassing parasite resistance. With this knowledge gap, this article seeks to explore the effect of three drug resistant mutations Y268C, Y268N and Y268S on the PfCytb structure and function in the presence and absence of ATQ. To draw reliable conclusions, 350 ns all-atom membrane (POPC:POPE phospholipid bilayer) molecular dynamics (MD) simulations with derived metal parameters for the holo and ATQ-bound -proteins were performed. Thereafter, simulation outputs were analyzed using dynamic residue network (DRN) analysis. Across the triplicate MD runs, hydrophobic interactions, reported to be crucial in protein function were assessed. In both, the presence and absence of ATQ and a loss of key active site residue interactions were observed as a result of mutations. These active site residues included: Met 133, Trp136, Val140, Thr142, Ile258, Val259, Pro260 and Phe264. These changes to residue interactions are likely to destabilize the overall intra-protein residue communication network where the proteins’ function could be implicated. Protein dynamics of the ATQ-bound mutant complexes showed that they assumed a different pose to the wild-type, resulting in diminished residue interactions in the mutant proteins. In summary, this study presents insights on the possible effect of the mutations on ATQ drug activity causing resistance and describes accurate MD simulations in the presence of the lipid bilayer prior to conducting inhibitory drug discovery for the PfCytb-iron sulphur protein (Cytb-ISP) complex.

## 1. Introduction

Malaria continues to be a life-threatening disease caused by an intracellular protozoan parasite of the genus *Plasmodium,* transmitted by an infected female anopheles mosquito [1]. Five species of the *Plasmodium* genus are known to cause malaria in humans, namely *Plasmodium falciparum*, *Plasmodium vivax*, *Plasmodium ovale*, *Plasmodium malariae* and *Plasmodium knowlesi* [1,2]. Among the five *Plasmodium* spp., *P. falciparum* remains the most prevalent and lethal. The widespread resistance of *P. falciparum* parasite to available antimalarial drugs poses one of the greatest threats to malaria control [1,3]. Currently, the following antimalarials are clinically approved as first-line treatment for uncomplicated *P. falciparum* malaria. These are artemisinin-based combination therapies including artemether-lumefantrine (AL), amodiaquine-artesunate (ASAQ), mefloquine-artesunate (MQAS) and dihydroartemisinin-piperaquine (DHA-PQ) [1]. Additionally, there are six recommended chemoprophylactic drugs: atovaquone (ATQ)/proguanil, chloroquine, doxycycline, mefloquine, primaquine and tafenoquine (www.cdc.gov/malaria/travelers/drugs.html, accessed on 1 February 2021). Most antimalarials target the erythrocytic stages of the parasite with most crossing different cell membranes, including host cell membranes, to access their intracellular targets. However, there are some that target integral membrane proteins including but not limited to the *P. falciparum* cytochrome bc_1_ (cytbc_1_) complex [4]. ATQ is one such drug that is used to prevent and treat the disease. It functions by inhibiting the cytbc_1_ complex and causes a collapse to the mitochondrial membrane potential [5,6]. Unfortunately, the rapid appearance of ATQ-resistant malarial parasite strains has rendered the drug ineffective [7], indicating the need to design new efficacious malarial drugs. However, the priority should not only focus on designing effective drugs, but also on drugs capable of bypassing parasite resistance. Thus, elucidating the molecular effects of mutations on protein targets and understanding the resistance mechanisms is highly crucial in the initial steps of in silico drug discovery, hence the emphasis of this article.

**Figure 1:**
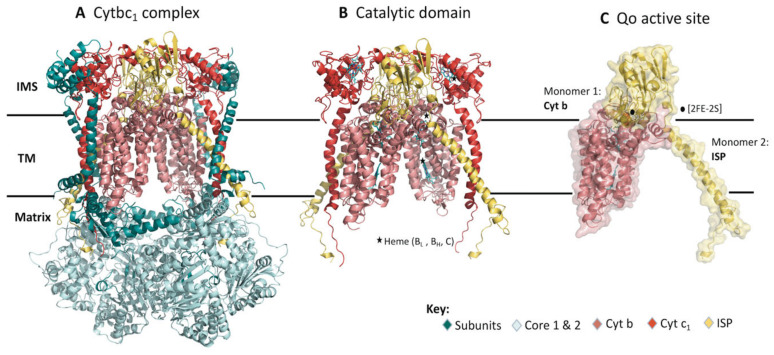
The structural model of the dimeric bc_1_ complex in bovine (PDB ID: 1PP9). (**A**) The crystal structure in cartoon representation showing the multi-subunit dimer. The different subunits are color coded (key provided) and labeled accordingly. (**B**) Catalytic domain composed of Cytb, cyt c_1_ and ISP spans across the inner mitochondrial membrane. (**C**) Heterodimeric structure made up of Cytb and ISP both of which form the Qo active site. Black stars and circles represent the hemes and [2FE-2S] cluster, respectively. (IMS—Intermembrane space, TM—transmembrane).

The structure of the cytbc_1_ is a homodimer embedded in the inner mitochondrial membrane (IMM). The IMM of *P. falciparum* parasite is mainly composed of phosphatidylcholine (PC), which is the most abundant phospholipid comprising 40%, followed by phosphatidylethanolamine (PE) (30%), cardiolipin (CL) (15%), phosphatidylserine (PS) and phosphatidic acid (PA) (5%) and other lipids in small portions such as sphingolipids, phosphatidylinositol (PI), phosphatidylglycerol (PG) and cholesterol [8,9,10]. Figure 1A shows the *Bos taurus* (PDB ID: 1PP9) schematic representation of the cytbc_1_ structure complex. The bovine and human cytbc_1_ proteins share 79% sequence identity. In *P. falciparum*, the homodimer consists of a total of 20 subunits with each monomer composed of 10 subunits having five low-molecular-weight, two core and three respiratory subunits. The respiratory subunits form the catalytic domain consisting of cytb, cytochrome c_1_ and the iron sulphur protein (ISP) which are embedded in the IMM as shown in Figure 1B [11]. The rest of the subunits in the enzyme complex are structural units essential in stabilizing the protein structure [12]. The quinol oxidation (Qo) active site is displayed in Figure 1C.

**Figure 2:**
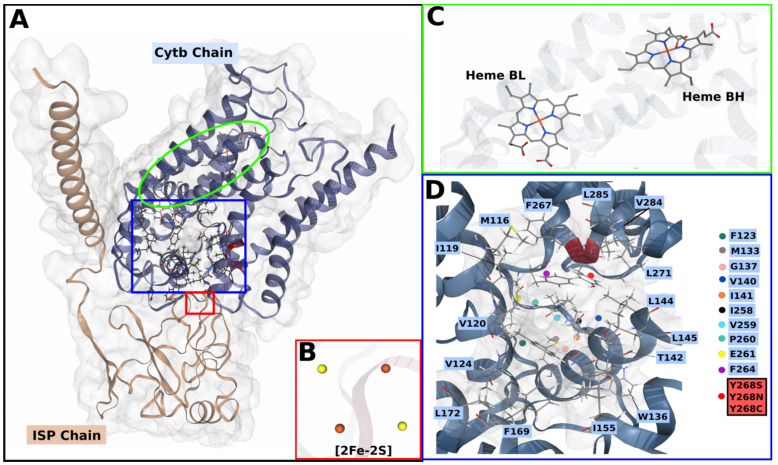
3D cartoon representation of PfCytb-ISP protein complex. (**A**) PfCytb-ISP structure with different subunits color coded (key provided) and labelled accordingly. The red, green and blue delimitations indicate the [2FE-2S] cluster, the heme groups and the active site, respectively. (**B**) [2FE-2S] cluster, showing sulphur atoms in yellow and Fe^2+^ atoms in orange. (**C**) Heme bL and bH groups. (**D**) The zoomed in active site showing its contributing residues which are highlighted in blue. The point mutations at position 268 are shown in the red colored box and indicated by a red sphere in the active site. This position is also colored red on the cartoon structure.

Figure 2A shows an expanded description of the heterodimer forming the Qo active site comprising of cytb that has a total of eight transmembrane (TM) helices, while the ISP subunit has one alpha-helix and an extrinsic domain. The active site is in the IMM towards the ISP chain interface where a total of three cofactors are located. These include; a [2FE-2S] cluster and two b-type hemes, heme bH and heme bL, where subscripts H and L refer to high- and low-potential, respectively, as displayed in Figure 2B,C. The two b-type hemes are intercalated in the transmembrane helices of the cytb subunit while the [2FE-2S] cluster is embedded in the extrinsic domain of the ISP chain, between two loops held together by a disulphide bridge [13,14].

The cytbc_1_ complex, a component of the electron transport chain (ETC), functions to transport electrons across the IMM. The electron transfer (ET) is mediated by a proton motive Q (quinol) cycle which occurs across the IMM in most eukaryotes including *P. falciparum* [15]. The Q-cycle mechanism is carried out distinctly across two substrate binding sites: quinol oxidation (Qo) and quinone reduction (Qi) catalytic sites [16,17]. Once the ATQ drug binds at the Qo site, hydrophobic residue interactions are formed. The active site residues (*P. falciparum* numbering) shown in Figure 2D include: Met116, Ile119, Val120, Phe123, Val124, Met133, Trp136, Gly137, Val140, Ile141, Thr142, Leu144, Leu145, Ile155, Phe169, Leu172, Ile258, Val259, Pro260, Glu261, Trp262, Tyr263, Phe264, Phe267, Tyr268, Leu271, Val 284, Leu285 [18]. To facilitate the ET process, the metal cofactors such as the [2FE-2S] cluster are involved although they are located ~20 Å away from cytb subunit [19,20]. Here, the extrinsic domain of the ISP chain having the cluster as shown in Figure 2A undergoes an approximate 65° rotational displacement. This domain movement allows the cluster center to move towards the cytb interface to support the ET process [17,21]. Overall, the generated proton gradient is essential in the subsequent production of adenosine triphosphate (ATP), which is crucial for parasitic cellular processes.

Owing to the previous success in targeting the *P. falciparum* cytbc_1_ complex, and it being an integral membrane protein, the protein remains an attractive drug target. Computational methods have since been used in drug design to search for novel antimalarial inhibitors as well as to understand drug action and resistance mechanisms [22,23]. Parasites have since evaded drug action, suggesting their fitness cost to the available drugs resulting in the parasites’ ability to evade drug action mechanism due to the compensatory nature of the mutations. This fitness cost has been proven by measuring the transmissibility of drug resistant or sensitive parasites through feeding of mosquitoes, where there was an increase in infection in those that fed on gametocytes with a specific mutation as compared to wild-type (WT) [24,25]. On ATQ drug action, one such study reports that once the ATQ drug binds to the Qo active site, the ISP domain movement necessary for electron transfer is blocked [26]. The molecular basis of resistance to ATQ has been reported in literature [18,27] where computational and clinical studies link the drug resistance to point mutations. These studies have associated ATQ drug resistance with Y268S point mutation [7,18,28,29,30,31,32]. This compromises the ATQ drug interactions mentioned above by causing a reduction or loss of residue contacts as well as altering the active site pocket volume [33]. There are various studies that report other mutations besides Y268S, that have since been associated with ATQ treatment failures. These mutations include Y268C and Y268N that also occur at the same residue position as the Y268S mutation [7,28,29,34]. While the mechanism of ATQ resistance has been elucidated, focusing on the Y268S mutation model, that of Y268C and Y268N is still not well understood in PfCytb-ISP protein as most studies focus on the Y268S mutation [18,32,33]. However, previous study used a bacterial (*Rhodobacter capsulatus*) system to understand the effect of Y268C (Y302C; bacterial numbering) on ATQ drug where the ability to block the ISP domain movement was lost as a result of this mutation [26]. Thus, studies on *P. falciparum* mutations in PfCytb-ISP are necessary to give more insights on the effect of these mutations on both protein and ATQ activity. The well-known point mutation Y268S as well as other reported mutations (Y268C and Y268N) in the PfCytb subunit are shown in Figure 2D.

Previous in silico studies on ATQ resistance mechanisms have failed to account for the *P. falciparum* cytochrome b (PfCytb) protein embedded in a membrane lipid bilayer. In addition, some of these studies have also only assessed the Y268S mutation using the yeast cytbc_1_ complex as a homologous model [27,32,33]. As such, there remains insufficient information in describing PfCytb protein dynamics embedded in a phospholipid bilayer and within the parasite. As a result, the effect of all the reported mutations (Y268C, Y268N and Y268S) on this protein and ATQ drug activity is still not well understood. Thus, it is crucial to mimic in vivo conditions by incorporating the respective protein in a lipid bilayer in analyses such as protein molecular dynamics (MD) simulations. Ultimately, this provides an accurate interpretation of the protein’s structure and function. However, there are several challenges faced during membrane MD calculations including setting up lipid membrane residues which are more complex and make up a large portion of the simulation system. Also, the assembly of the lipids to form a membrane as well as embedding the protein within the membrane in the correct orientation is challenging [35,36]. Nevertheless, with the recent development of computational tools, MD simulations of membrane proteins have become more viable in understanding protein membrane dynamics and interactions [37,38,39].

For the first time, to the best of our knowledge, our study presents insights on the effect of mutations on PfCytb-ISP protein as well as on ATQ activity occurring at the Qo active site. Using in silico methods, experiments were performed on the holo and ATQ-bound protein complexes embedded in monounsaturated 1-palmitoyl-2-oleoyl-sn-glycero-3-phosphocholine (POPC) and 1-palmitoyl-2-oleoyl-sn-glycero-3-phosphoethanolamine (POPE) bilayers via MD. Using the bonded model approach, force field parameters were derived and validated for both b-type hemes and [2FE-2S] cluster cofactors located in PfCytb-ISP Qo catalytic center, after which MD simulations were performed. Incorporating force field parameters and the phospholipid bilayer in this in silico study ensured that the PfCytb-ISP Qo active site was well described. Dynamic residue network (DRN) analysis was used to investigate the effect of these point mutations, as we proposed previously [40,41]. Consistent with previous findings, point mutation Y268S was reported to affect key active site residues where these residues were observed to lose communication. Likewise, similar resistance mechanisms were noted in both the Y268C and Y268N mutant systems. Ultimately, this approach and findings provide insights into the effect of point mutations Y268C, Y268N and Y268S on PfCytb-ISP protein and ATQ activity, hence its importance to future structure-based virtual screening studies and MD simulations.

## 2. Results and Discussion

### 2.1. Protein Structure Modeling

Due to the absence of the WT plasmodial cytbc_1_ complex x-ray crystal structure, both PfCytb and ISP subunits were separately modeled using I-TASSER (Iterative Threading Assembly Refinement) [42,43,44], and the structural templates were 3CX5 [45], IBGY [46], IPPJ [47], 1EZV [48] and 1PP9 [47] from the Protein Data Bank (PDB). Using HHpred [49] and PRIMO [50], the aforementioned templates had respective sequence identities of 37%, 37%, 41%, 37% and 41% for PfCytb subunit whereas for the ISP subunit, respective sequence identities of 43%, 43%, 43%, 43% and 43% were observed. 1PP9 (*Bos taurus*) having an experimental resolution of 2.1 Å was selected as the main template and used to guide the modeling process [49]. The precision of the modeled structure has been suggested to be more refined upon the use of several templates and this was utilized in this study to improve model quality and resolution of more mobile protein segments [51,52]. After modeling, the quality of the structures was further assessed using the in-built I-TASSER metrics, Ramachandran plot and z-DOPE score.

I-TASSER uses two parameters to evaluate modeled proteins. These include; TM-score which gives a global overview of the protein topology in terms of structural similarity between two proteins, and the confidence (c-score) scoring algorithm, which generates confidence estimates in the overall quality of the predicted model. A TM-score > 0.5 indicates models having correct topology as opposed to <0.17 which signify random topology [42]. C-score is calculated based on clustering structural density, a consensus on multiple threading templates and the convergence of structure assembly, and ranges from −5 to 2; higher values denote higher quality. The TM-score algorithm ensures the least coordinate difference in each modeled protein is measured [53]. TM-scores of 0.99 ± 0.04 and 0.74 ± 0.12 were reported for the PfCytb and ISP chains, respectively. A c-score of 2 was reported for both models denoting good quality [42].

A Ramachandran plot of the proteins’ psi (φ) and phi (ψ) main-chain torsion angles identifies the amino acids with acceptable torsion angles while identifying the outlying residues [54]. The Ramachandran plot showed that 88.9% of the total residues for PfCytb and 83.4% of ISP modeled subunits were in the most favored regions, while 0.3% and 0.7% of residues, respectively, were in disallowed regions. These plots are presented in Figure S1 [55]. The z-DOPE score was then used to evaluate the modeled PfCytb-ISP protein complex. The calculated z-DOPE score of the complete PfCytb-ISP protein was −0.76. The score was close to −1.0 indicating that the model was of high quality. It has been reported that with careful model validation the use of homology models can be reliable and in good accordance with experimentally derived structures [56]. In the present study, the predicted structure quality was shown, by use of several validation metrics, to be suitable for the subsequent experiments.

A comprehensive literature search of ATQ resistance mutations identified Y268C, Y268N and Y268S to be associated with drug resistance. To generate these mutant proteins, the modeled WT protein structure was subsequently mutated using Discovery Studio Visualizer to generate the respective mutant protein models. Model evaluation determined Y268C, Y268N and Y268S to be associated with z-DOPE scores of −0.76, −0.75 and −0.75, respectively.

### 2.2. Mutant Residue Analysis on Static Structure

Following ATQ treatment failures, clinical studies have linked resistance to the reported point mutations; out of which Y268S is more prevalent followed by Y268C and Y268N [29,30,34]. The Y268S point mutation has been shown to compromise hydrophobic interactions between ATQ and amino acids as well as alter the active site pocket volume [33,57]. To provide more insights on the potential effect of mutation on the PfCytb-ISP protein, the location of these point mutations, shown in Figure 2D, was investigated with reference to protein secondary structure. Point mutations Y268C, Y268N and Y268S are located on an alpha helix within the active site vicinity. The amino acid substitution from Tyr to Cys, Asn and Ser does not alter the secondary structure of the protein. However, these mutations have been implicated to alter the active site pocket volume. Qo active pocket analysis using CASTp server [58,59] reported pocket volumes of 675.7, 726.6, 718.8 and 721.2 for WT, Y268C, Y268N and Y268S, respectively. This increased pocket volume observed in all mutant systems compared to the WT is likely to lead to loss of crucial hydrophobic drug interactions, thus interfering with expected drug binding [33]. For instance, Tyr substitution to either Cys or Ser has been reported to decrease the hydrophobic interactions, whereby the aromatic group present in Tyr is replaced by nucleophilic side chains thus decreasing the hydrophobic interactions.

**Figure 3:**
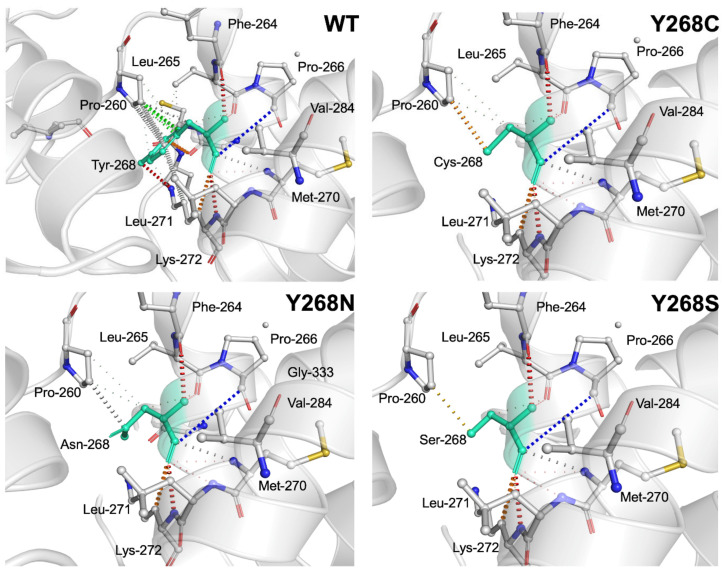
Illustration of interatomic interactions occurring around residue 268 in the WT (wild-type), and Y268C, Y268N, Y268S mutant proteins. Residue 268 in the WT and mutant proteins is colored in light green and shown as sticks. The respective chemical interactions are labeled as dotted lines and colored as follows: Hydrogen bond—(red), weak hydrogen bonds—(orange), hydrophobic contacts—(green), amide-amide contact—(blue) and ionic interactions—(gold). Amino acid residues are also colored according to type, namely; nitrogen (blue), oxygen (red) and sulphur (yellow).

VAPOR (Variant Analysis Portal) [44] prediction tool was used to predict the effect of mutations on the function and stability of PfCytb-ISP protein. From these analyses, three scoring metrics were calculated and reported as shown in Table 1. The ΔΔG values obtained from both I-Mutant [60] and MuPro [61] were close while slightly differing from DynaMut [62]. Nevertheless, all these metrics show decreased protein stability in all three mutations. Data in Table 1 also illustrates that mutations are predicted to cause stability reductions within their respective proteins. Further, prediction of the effect of these mutations on the interatomic interactions occurring in their surrounding was calculated using DynaMut and the results are shown in Figure 3.

Figure 3 shows various interactions formed in the PfCytb-ISP WT active site. The Tyr residue in the WT was observed to form the highlighted residue interactions with its surrounding residues, whereas most of these interactions were seen to be lost in all three mutants. Interestingly and consistent with previous findings [33], hydrophobic contacts colored in green are seen to be lost, evidencing the detrimental effect of these mutations in affecting protein function. In the WT and mutant proteins (Y268C, Y268N and Y268S) as indicated by respective colors, these interactions include: hydrophobic contacts in green (Tyr268-Pro260), weak polar contacts in red (Tyr-Phe264/Lys272), amide-amide contact in blue (Tyr268-Val284), weak van der Waal (vdW) interactions in grey (Asn268-Pro260/Met270, Cys268-Met270), water mediated weak hydrogen bond in orange color (Tyr268-Leu271, Cys268-Pro260) and ionic interactions in gold color (Ser268-Pro260). In comparison to interatomic interactions in WT, vdW contacts (Pro260, Leu265 and Leu271) were observed to be lost in all three mutant proteins.

Figure 3 shows the interactions formed between the Tyr residue in the WT protein and corresponding residues in the mutated proteins (Y268C, Y268N and Y268S) as indicated by respective colors. These interactions include; hydrophobic contacts in green (Tyr268-Pro260), weak polar contacts in red (Tyr-Phe264/Lys272), amide-amide contacts in blue (Tyr268-Val284), weak van der Waal (vdW) interactions in grey (Asn268-Pro260/Met270, Cys268-Met270), water mediated weak hydrogen bonds in the orange color (Tyr268-Leu271, Cys268-Pro260) and ionic interactions in the gold color (Ser268-Pro260). In comparison to interatomic interactions in WT, vdW contacts (Pro260, Leu265 and Leu271) were observed to be lost in all three mutant proteins.

### 2.3. Derived Force Field Parameters

Prior to force field calculations, the entire protein was protonated at an optimal pH of 7.0 at which the protein is active and stable. The p*Ka* values of the ionizable groups (His and Cys) in the PfCytb-ISP metal center (only the coordinating residues) are shown in Table S1. Additionally, the protonation states of all ionizable groups were evaluated and reported as in Table S2 as they constitute essential parts of molecular surfaces.

The WT protein model was used to calculate force field parameters for the heme bL as [2FE-2S] cluster cofactors. Density functional theory (B3LYP/6-31G*) calculations, as explained in the methodology section, were performed to obtain all force field parameters including but not limited to bond distance, angle, force constants and RESP charges. Of keen interest is the metal bond distance which was the metric used to validate the derived parameters. A total of 10 coordinate bonds were included in the calculations for all interacting atoms, and residues involved in these bonds include: Cys299, His301, Cys317 and His320 for the [2FE-2S] cluster and planar N atoms (NA, NB, NC, and ND), His78 and His173 for the heme bL.

**Figure 4:**
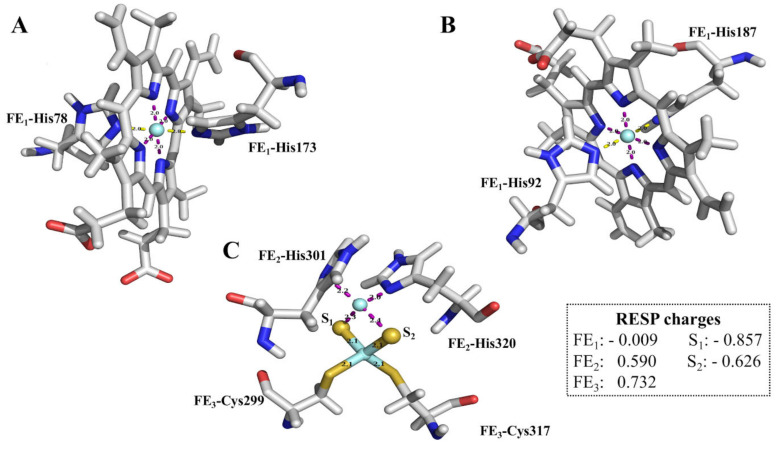
Graphical stick representation of the Quantum mechanics optimized sub-structures of the three prosthetic groups (**A**) Heme bL; (**B**) Heme bH; (**C**) [2FE-2S] cluster. Fe^2+^ metal, nitrogen, sulphur, oxygen and carbon atoms are shown in cyan, blue, mustard, red and grey color, respectively. The dashed purple lines represent the chemical bonds.

Restrained electrostatic potential (RESP) charges for the metal ion, sulphur atoms and interacting atoms are shown in Figure 4, while RESP charges for all coordinating atoms are provided in Table S3. Parameter and coordinate files for the cofactors and all interacting residues are displayed in Table S4.

The derived Fe^2+^ metal charges in the [2FE-2S] cluster were reported to have positive values of 0.590 (FE_2_) and 0.732 (FE_3_), which is consistent with previous studies [63]. However, FE_1_ in b-type heme bL was reported to have RESP charges of −0.009, unlike previous studies that reported the RESP charge for Fe^2+^ to be 0.16 using the HF/6-31G* charge calculation method [64]. Additionally, other studies have reported a charge of 0.45 and 1.58 in a-type and c-type heme structures respectively [65,66]. Heme is axially coordinated by two His residues while c-type heme is axially coordinated by His and Met residues. Despite this observation, it was noted that the low charges did not destabilize the metal coordination sphere of the entire protein. This relatively low Fe^2+^ charge in our study could be a result of the delocalization of the electrons which brings about a stabilizing effect [67]. On the other hand, sulphur partial atomic charges reported in our study were similar to previous literature [66].

The derived bond distances and associated force constants for heme bL (whose calculations also represent heme bH) and [2FE-2S] cofactors calculated are shown in Table S5. Comparison of the mean metal and coordination atom distances data for template 1PP9 and QM derived (pre-MD simulation), show similar values, suggesting an accurate metal coordination environment in the heme bL and [2FE-2S] cluster. These results agree with previous studies [63,68,69,70]. Force constants in the FE_1_-NE2 (His78) and FE_1_-NE2 (His173) bonds were reported to be 42.7 and 43.0 kcal mol^−1^ Å^−2^, respectively, while FE_3_-SG (Cys299) and FE_3_-SG (Cys317) bonds had force constants of 131.0 and 118.6 kcal mol^−1^ Å^−2^, respectively. The similar trend observed in energy profiles in these bonds suggests similar binding properties to Fe^2+^.

Detailed information on bond angles and other associated parameters within the coordination sphere of heme bL and [2FE-2S] cluster is presented in section h of Table S4. Bond angles in the [2FE-2S] cluster involving the sulphur atoms such as S1-FE_2_-S2, S1-FE_3_-S2, FE_2_-S1-FE_3_ or FE_2_-S2-FE_3_ were observed to be consistent with the literature [63]. Angles of bonds coordinating the Fe^2+^ in heme bL showed some deviations compared with previous studies [68]. Structural features could explain the varied observation as calculations in this study used a modeled protein while an x-ray crystal structure (PDB ID: 1YCC; yeast iso-1 cytochrome c) was used in previous studies. Also, this could be accounted for by the different axial ligands in their coordination geometry observed in the different heme groups [65,66]. Despite this observation, the derived values did not cause any geometry distortion during the optimization process.

### 2.4. Validation of the Force Field Parameters; MD Simulations

The derived force field parameters were further evaluated via membrane MD simulation using the AMBER software package [71] to investigate whether the cofactors would be maintained within the protein. Cofactor bond distances were also measured, and the results are presented in Table S5 (post-MD column). Additional standard deviation values are provided for the bond distances representing all sampled points across 350 ns simulations. The average bond distances of all the 10 coordinate bonds involving His and Cys residues of the cofactors were 2.06 Å and 2.13 Å, respectively. This is close to the reported experimental mean bond distances of 2.16 Å (FE_1_-N) and 2.30 Å (FE_3_-SG) [72]. Results evidence that pre-MD and post-MD bond distances have similar values. In addition, MD trajectory analysis highlighted that the octahedral and tetrahedral coordination geometries of the cofactors were maintained within the protein. The bonds lengths over the 350 ns simulation are further presented in Figure S2.

The behavior of all 10 Fe^2+^ coordinated bond distances were monitored throughout the simulations in all the mutant systems as represented in Figure S3. These bond distances were found to be comparable to those observed in the WT system. Additionally, intra-comparison of all protein systems in triplicate runs showed that there was a subtle change in their bond distances. Overall, the bond distances were comparable (*p*-value = 0.7302), and therefore reliable for use in MD calculations.

### 2.5. ATQ Docking Analysis, Trajectory Analysis and Dynamics of the Ligand

**Figure 5:**
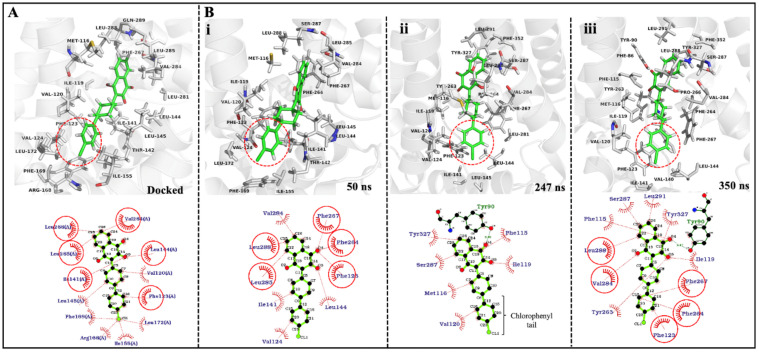
3D and 2D ligand interactions of ATQ drug in docked pose and across different time steps during 350 ns simulations. (**A**) ATQ docked pose and its interaction within 5 Å radius around ATQ drug in the PfCytb-ISP Qo active site pocket; ATQ in green while active site residues in gray. (**B**) ATQ-bound protein and ATQ interactions during three time steps of the MD simulation. The 3D figures show Qo active site pocket of PfCytb-ISP protein in gray while ATQ drug is shown in green as stick representation in both 3D and 2D representation. Red dotted circle represents the chlorophenyl tail of the ATQ drug.

Docking of the ATQ drug into the Qo site of the PfCytb-ISP protein was performed as detailed in the methodology section. The best docked pose and interactions of ATQ, are shown in Figure 5A. The ATQ drug exhibited a binding energy of −7.7 kcal/mol. The final docked pose showed ATQ bound in the Qo active site with its cyclohexyl group facing upward and away from the heterodimer interface of the protein complex.

**Figure 6:**
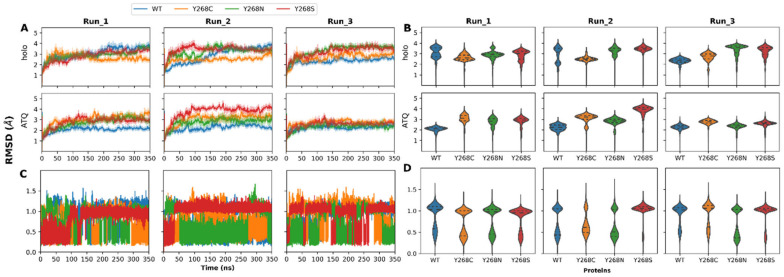
Molecular dynamics trajectory analysis of the changes to protein root mean square deviation (RMSD) both in the holo and ATQ bound systems. (**A**) Protein RMSDs over the 350 ns simulation; (**B**) Protein RMSD violin plot showing conformational distribution and sampling; (**C**) Ligand (ATQ drug) RMSD over the 350ns simulation; (**D**) ATQ RMSD violin plot presenting conformational sampling and distribution.

Figure 5B shows 3D (3 dimensional) and 2D (2 dimensional) ligand interactions in different time steps (50 ns, 247 ns and 350 ns) during MD simulation of the WT system. Here, the ligand interactions within 6 Å around the drug were investigated across each of the time steps (Figure 5B). Additionally, the trajectory was visualized and ATQs chlorophenyl tail was shown to keep flipping throughout the MD simulations in both WT and mutant systems. Figure 5 accounts for this flipping which is illustrated by the red circle (in 3D) as well as rotation exchange of atomic position C20 and C21 (in 2D). These findings agree and further explain the ligand RMSD behavior in Figure 6C, where RMSD jumps were observed across the simulations in all systems while the drug remained bound in the Qo active site. Ligand RMSD will be further discussed in Section 2.6.

### 2.6. Trajectory Analysis of PfCytb-ISP Inside POPC:POPE Bilayer; Holo and ATQ Bound Complex

Prior to MD simulations, inspection of the membrane was performed and revealed that in both leaflets the ratio of POPC to POPE remained approximately 1.3:1 (1.28–1.29). All lipids were accurately constructed, and none were missing head groups or tails. MD simulations were then performed, and the behavior of the waters and lipids closely monitored. Throughout the simulations it was observed that none of the water molecules entered the membrane, illustrating accurate experimental setup and membrane packing.

Upon confirming accurate MD simulations, protein dynamics was then investigated. The changes to RMSD and Rg between the WT and mutant proteins during simulations were plotted (Figure 6A,B) and discussed below. The probability density and data distribution are also presented as violin plots in Figure 6A,B. From Figure 6A, it is observed that across all WT and mutant protein systems, most of the simulations were equilibrating (showing less than 2 Å deviations to RMSD) after a minimum of 50 ns. This effect was most likely due to the increased complexity of the POPC:POPE within each membrane leaflet. As a result, MD simulations were performed over 350 ns to obtain sufficient conformational sampling. In general, simulations over 100 ns are regarded as a good estimate in monitoring protein dynamics [73]. Previous in silico studies by Akhoon et al. [18] (performed without heme and a phospholipid bilayer) included protein dynamics for only the Y268S protein where the mutant RMSD value in the system equilibrated at a higher value compared to the WT [18]. This corroborates with our studies, and the trend is more evident when ATQ is bound to the proteins, except for the holo Y268C systems.

With respect to the holo proteins, RMSD conformational sampling distributions (Figure 6B) present multi-modal distributions for the WT and mutant proteins. This suggests that during MD simulation, the proteins sampled multiple distinct conformations. Comparison of the WT to mutant proteins highlights that although protein distributions and RMSDs of the most sampled conformations may differ, the median structures of proteins across all runs are within 1.0 Å of each other. Worth noting are the presence and number of flexible loop regions in the protein. The simulated PfCytb-ISP protein complex accounts for a total of 33% loop regions which could explain the differences observed in the RMSDs. This, therefore, indicates that core region of the structures shares some degree of conformational similarity. Since the respective mutants confer drug resistance in the parasite, the relative degree of conformational similarity may suggest that the mechanism of resistance may not significantly affect the global protein structure but may have greater effects on the local residue structure.

ATQ presence is associated predominantly with unimodal sampling distributions compared to the holo proteins. This suggests that the binding of ATQ may have a stabilizing effect on the proteins, and this effect is more observable within the first two runs of the holo WT protein that show multiple conformations. From that data it is also observed that Y268S shows different behavior among all three runs. The first run evidences similar median RMSD whereas in the second run the ATQ bound protein is associated with higher median RMSD, whereas in the third run the ATQ bound mutant is associated with a lower median RMSD. This may indicate differing variant mechanisms of resistance when ATQ binds. This is further supported by Y268S having similar median RMSD values across all three runs for the holo protein.

In addition, average median RMSD values and their differences were calculated for all triplicate runs in each protein system to determine which system deviated the most from holo WT protein. The average median difference between WT and mutant systems was performed by subtracting the mutant average median RMSD from the WT protein. From these analyses, the average median values of 3.06, 2.66, 3.35 and 3.38 Å were observed for WT, Y268C, Y268N and Y268S systems, respectively. Additionally, the average median differences were reported as follows: 0.40, −0.29 and −0.32 for mutant systems Y268C, Y268N and Y268S, respectively. This indicates that mutant system Y268C exhibited the largest deviation from the WT system as it sampled lower RMSD values in all triplicate runs as compared to the WT. A marked difference in previous studies [18,33] focusing only on the Y268S mutation is that, in this study, all protein systems were embedded in a lipid bilayer. Here, our results presented protein dynamics for all the three mutant systems in comparison to the WT. The RMSD differences observed in these mutant systems show the varied magnitude of the point mutations on protein’s structure and behavior.

The RMSDs of the ATQ ligand with respect to protein in WT and mutant proteins over the 350 ns simulations are presented in Figure 6C,D. In each case, the entire trajectory was assessed where the ATQ ligand was observed to adopt different poses in the mutant proteins as compared to the WT. Various pose changes are highlighted by shifting RMSD values. Pose changes to ATQ with exception to Y268N (second run) and Y268S (first two runs) are observed. This could be indicative of increased ligand stability. Due to these pose changes, it is possible that within the mutant proteins, ATQ likely loses the ability to block ISP domain movement, which is a mechanism of action that has been reported in previous literature [26].

The Rg line graph and violin plots in Figure S4A,B suggest that Y268C and Y268S mutations might result in more compact proteins than the WT and Y268N systems. This trend is, however, not observed within the ATQ bound structures, suggesting that the presence of ATQ influences the compactness of the proteins. However, unlike the RMSDs of the holo proteins, the Rg violin plots show unimodal distributions suggesting that there were no major changes to the protein center of mass during MD simulation. Results in Figure 5 demonstrate Rg values ranging from 26.5 to 27.5 Å in all systems indicating relatively similar compactness across all protein systems in all triplicate runs. Although RMSD and Rg differences of less than 1.0 Å indicate similar structures, this difference could still be important. The significance of this difference to protein structure and function is further investigated throughout this article.

### 2.7. Conformational Entropy of the WT and Mutant Proteins; Holo and ATQ-Bound Complex

**Figure 7:**
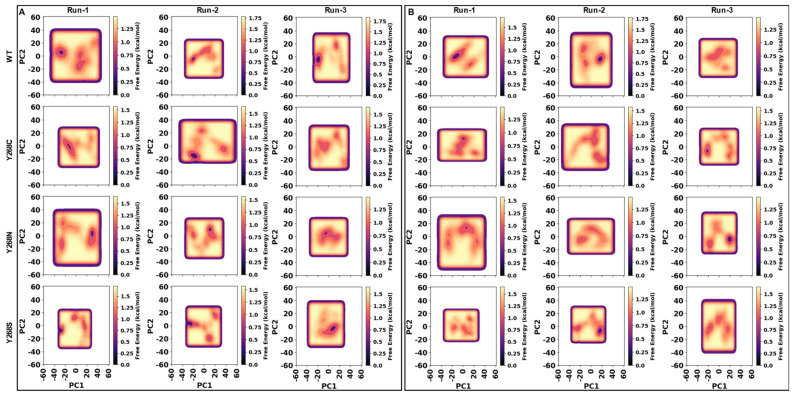
Principal component analysis (PCA) of the holo and ATQ bound proteins. (**A**) Holo; (**B**) ATQ bound protein. The plots show a 3-dimensional (3D) plot of PC1 versus PC2 of the WT and mutant holo proteins as a function of free energy. All protein systems were shown in three replicate runs (Run-1, Run-2 and Run-3). Each scale corresponds to each individual plot where the black color at 0 kcal/mol represents lowest free energy conformations.

Results in Figure 7 show the plots of PC1 and PC2 and associated free energy of each respective conformation in both the holo and ATQ bound systems. Within the holo proteins (Figure 7A), analysis of the WT protein across the three runs reveals that the protein adopts one major conformational cluster of low energy which evidences potential stability within the WT protein. This trend is also noted for all the mutant proteins with exception to Y268C in Run-3 that did not show any evidence of low energy conformations. The single low energy wells within the mutants are however expected. Since the point mutations are associated with drug resistance, it is expected that their presence would not be associated with greater instability within the proteins as that could be detrimental to function.

In the WT protein similar low energy conformations are observed across all runs along PC1 from −20 to −30. Even along PC2 these conformations maintain similarity. Y268C shows similar conformational clusters with low free energy in the first two runs along PC1. Interestingly comparison of the PC1 values with the WT protein reveal similar conformational sampling in 3D space between the proteins. This suggests that although mutant presence confers ATQ resistance, the protein is still able to adopt conformations of low free energy and is structurally similar to that of the WT. Mutant Y268S also shows a similar trend to that of Y268C for PC1 within the first two runs. This may highlight potential similar mutant mechanisms within these two mutants.

Inspection of Y268N may highlight a possible mechanism of resistance of the mutants. Along PC1 the protein adopts low energy conformations at approximately PC1 values of approximately 15–30 in the first two runs which also coincide with the low energy conformation observed in Run-3 of Y268S. Comparison of the Y268N result to that of the WT protein highlights two possible mutant mechanisms. Firstly, the mutant proteins could adopt another protein conformation capable of function and resistance to ATQ. Secondly, the WT protein shows slightly higher free energy conformations at this respective PC1 value (15–30), and mutant Y268N also shows higher energy conformations that coincide with the low energy wells of the WT protein (PC1 of −20 to −30). These two factors suggest that the mutant can occupy a functionally active protein conformation similar to that of the WT but less energetically favorable. Biologically, this could mean that this conformation is not as catalytically active nor abundant as that of the WT but still functional and could have implications for ATQ activity or binding propensity. More research is needed to confirm this, however, within the scope of the literature, experimental analysis has only been performed on the Y268S mutant.

When ATQ is bound (Figure 7B) it is observed that the WT protein structures sample lower energy conformations compared to the mutant proteins. Though Run-3 for the WT lacks a low energy structural well, the conformational sampling is like that of run 1 along both PC1 and PC2. Compared with the mutants, the larger size of the low energy wells (purple dots) indicates that during MD more WT structures occupy these low energy conformations than the mutants. This suggests that when ATQ is bound structural conformations are not as energetically favorable. Of interest is the WT conformation at PC1 of 15 when ATQ is bound. This conformation coincides with that observed for the holo Y268N. This indicates some degree of conformational similarity and could support the previously aforementioned potential resistance mechanism. Overall, factoring in the holo protein PCA results, data illustrates that the binding of ATQ to the mutant proteins results in an increase to free energy.

### 2.8. Effect of Mutations on Residue Flexibility

Mutation effects on residue flexibility were then investigated using RMSF, and the line graphs presenting the changes to RMSF during MD as shown in Figure S5. From the data, it was observed that the greatest RMSF changes are located towards the C-terminal of the PfCytb subunit at the intersection of the two chains as well as the loop regions (PfCytb chain: 21–27, 187–180, 207–213, 218–232, 265–285, 294–315, 334–339; ISP chain: 218–235, 238–243, 270–273, 277–282, 297–306, 317–343) of the PfCytb-ISP protein. Based on the number of loops highlighted in the PfCytb-ISP protein, the higher RMSD values observed in the previous section can also be attributed to the presence of loop regions, as previously reported [74]. The results of observations in Figure S5, however, vary between MD simulations for each case. With RMSF results not presenting any consistent major differences to residue flexibility of WT and mutant proteins among the MD runs, we decided to look at local residue analysis via residue contact map and the results are presented in Section 2.9.

### 2.9. Residue Contact Maps; Effect of Mutation on Protein Residue Interaction Network

**Figure 8:**
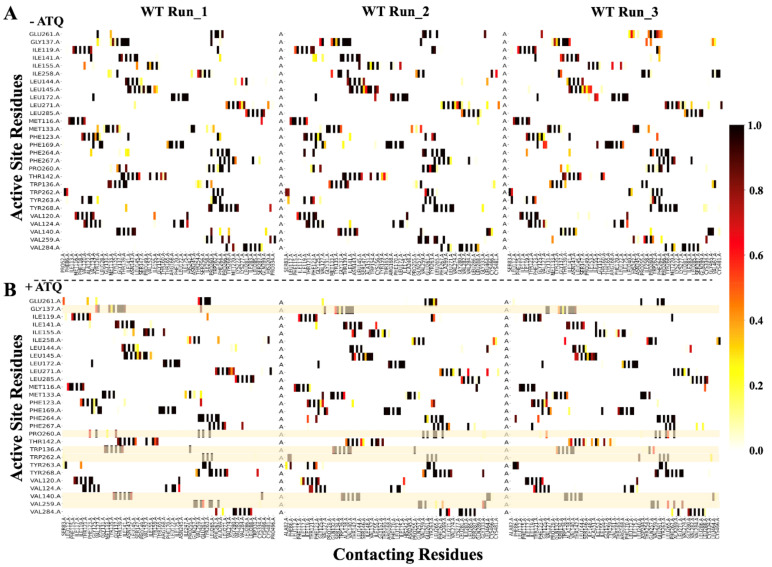
Residue contact heat maps for PfCytb-ISP active site residues. Heat map representations of the active site residues forming contacts with other residues (contacting residues) in (**A**) WT (wild-type) system across triplicate runs in holo protein structure and (**B**) WT system across triplicate runs in ATQ-bound protein structure. The residue contacts were monitored over during the last 100 ns of the simulation time. The yellow lines across the heatmaps in (**B**) highlight residues of either reduced or lost contacts relative to the WT. Color intensity is proportional to the occurrence of residues contact throughout the simulation, i.e., 0–1 on the color bar is equivalent to 0–100% MD simulation time. Zoomed figures comparing each of the mutant systems to WT in both holo and ATQ-bound forms are shown in Figures S6–S8.

To investigate whether the mutations affected immediate residue interactions within the active site and at residue position 268 in both holo and ATQ-bound proteins, local residue network analysis was performed. A contact cut-off distance of 6.7 Å and an iteration step size of 100 were applied to obtain all residue-residue contacts in the mentioned residue positions. The residue interaction changes were monitored throughout the equilibrated phase of MD simulations. This comprised the final 10,001 simulation frames. The percentage values of the residue contacts across simulation time were calculated by dividing the total per frame proportion of residue contacts by the total number of selected MD frames. Comparison was performed among mutant and WT systems in the triplicate runs for both holo and ATQ-bound proteins as presented in Figure 8, Figures S6, S7, S8 and S9, respectively. Unlike the RMSF results, the triplicate MD runs gave consistent results in the residue interaction level.

Residue contact maps of the triplicate runs in the WT for both holo and ATQ-bound was performed as shown in Figure 8. Additionally, residue-residue interactions in mutant systems Y268C, Y268N and Y268S were compared to those in the WT systems as shown in Figures S6–S8. Specifically, this highlights a robust comparison of the residue contacts formed at the active site where WT system was used as the reference. This was inspired by the fact that one of the effects of point mutations on drug binding and PfCytb-ISP active site is destabilization of the hydrophobic interactions [33,57]. As shown in Figure 8, residue-residue interaction analysis in the WT system was performed comparing holo and ATQ-bound proteins in triplicate runs. Across all triplicate runs, interaction Val140-Ile258 was lost in the ATQ-bound system. However, in at least two runs, the following interactions are seen to be either lost or reduced. The interactions reduced include: Val259-Cys334, Pro260-Phe264 and Trp262-Ser83. Those lost are Gly137-Pro260 and Trp136-Ile258 residue-residue interactions. Worth noting is that residue-residue interactions at position 268 were maintained in the ATQ bound protein.

A total of 28 active site residues were analyzed in the residue contact heat maps. These active site residue-residue interactions are important to protein structure and function including substrate binding. In the holo protein, four active site residues out of the total active site residues were reported to have compromised interatomic interactions in the mutant systems as shown in Figures S6–S8. These four unique residues include: Trp136, Gly137, Pro260 and Phe264. With these analyses, active site residue interactions that were compromised (reduced or lost) across three as well as in at least two mutant systems were reported and further discussed below.

Within the WT holo protein, the residue-residue contacts were observed to be maintained 100% of MD simulation time. Comparative analysis of mutant systems Y268C, Y268N and Y268S residue contact map reported reduction/loss in seven residue-residue contacts. The first contact (Trp136-Val140) was reduced in mutant systems (Y268C: 26%, Y268N: 79% and Y268S: 0%). The second contact (Trp136-Met133) was reduced in (Y268C: 59%, Y268N: 0% and Y268S: 16%) mutant systems. Residue-residue contacts of active site residue Trp136 to residues Gln132 and Pro260 were lost in all three mutant systems. The active site residue Gly137 was further assessed in Y268C and Y268N mutant systems, and a reduction in the third contact (Gly137-Ile141) for mutant system Y268C occurring at 70% of simulation time was found. We also observed that fourth contact (Gly137-Pro260) was reduced in Y268C and Y268N mutant systems where the respective contacts occurred at 50% and 1% of MD simulation time. Active site Pro260 was assessed in Y268N and Y268S systems where the fifth contact (Pro260-Phe264) was lost in two runs in the Y268N mutant system and reduced in Y268S mutant system occurring at 50% of the simulation time. Lastly, active site residue Phe264 was assessed where the sixth contact (Phe264-Asn/Ser268) was observed to be reduced in mutant systems Y268N and Y268S, occurring at 94% and 5% of the simulation time, respectively. From the Phe264 residue assessment, the seventh contact (Phe264-Pro260) was reduced to 38% and 50% in Y268N and Y268S systems, respectively. From this analysis, a total of four unique active site residues were affected considering the conservation nature of the bc_1_ activity at Qo active site.

On the other hand, comparative analysis displayed in Figures S6–S8 showed ATQ bound protein having a total of five active site residues that observed both reduction and gain in residue-residue interactions. This was analyzed in the mutant systems (Y268C, Y268N and Y268S) using WT as the reference. These residues include: Met133, Trp136, Thr142, Ile258 and Val259. On interactions that experienced a reduction, mutant system Y268C reported a reduction in Trp136-Gln132 in Run_1 and 2 occurring at 18% and 8% of MD simulation time, respectively. Mutant system Y268N reported interaction Met133-Asp244 to occur 15% and 28% of simulation time in Run_2 and 3, respectively. Lastly, mutant system Y268S reported a reduction in interaction Thr142-Val152 in Run_1 and 2 occurring at 1% of simulation time in each of the runs. Altogether, all mutant systems experienced compromised residue-residue interactions. These reduced and lost interactions will possibly compromise the substrate binding in the Qo active site altering protein function. These findings also support those of the active site volume (refer to Section 2.2), where all mutants are seen to have increased pocket volume which could lead to loss of some of the active site interactions as reported previously [33]. Overall, a total of five unique active site residues were affected in the mutant ATQ-bound proteins. This is a higher number than that reported by the holo protein. Ultimately, these lost/reduced interactions will likely interfere with the binding of the substrate. Evidently, we can link the compromised interactions to the presence of point mutations where the ability to block the ISP domain movement is impaired as reported in literature [26]. Therefore, it is worthy to note that besides the Y268S resistance mechanism which has been reported previously [18,33], our findings highlight point mutations Y268C and Y268N as having similar resistance mechanisms to Y268S, as observed in the compromised residue-residue interactions. Despite the reduced interactions experienced in all mutant systems interactions, there are two interactions that were gained in the Y268C and Y268S mutant systems. These include: Val259-Cys334 in Run_1 (100%) and Run_3 (87%) for Y268C as well as Ile258-Thr254 in Run_1 (93%) and Run_2 (86%) for Y268S mutant systems.

Similarly, Figure S9 highlights changes to local residue-residue contacts at the location of the mutations (residue position 268 only) in both holo and ATQ-bound proteins. The individual residue contact maps for WT and mutant systems in triplicate runs for both holo and ATQ-bound proteins are displayed in Figures S10 and S11. A cut-off distance of 6.7 Å and an iteration step size of 100 was applied in the calculations. Here, we report four distinct residue-residue contacts in the WT holo protein (Tyr268-Gly333, Tyr268-Val259, Tyr268-Pro260 and Tyr268-Phe264) that maintained contact for 66%, 90%, 99% and 99% of the simulation time respectively, that were compromised. Specifically, contact Tyr268-Gly333 was reduced in all three mutant systems (6%, 6%, 0%) while Tyr268-Val259 was occasionally observed in Y268S and Y268C mutant systems (24% and 3%) respectively. Finally, contacts Tyr268-Pro260 and Tyr268-Phe264 were reduced (0% and 8%) in mutant systems Y268N and Y268S, respectively. Also, contacting residues having reduced or lost residue interactions in the mutant systems include: Gly333 and Val259 for all mutant systems as well as Pro260 for the Y268N mutant system only. However, in ATQ-bound systems two distinct residue-residue contacts displayed reduced interactions. These include; Tyr268-Gly333 and Tyr268-Val259 which as compared to WT, the interactions were reduced in all mutant systems (Y268C, Y268N and Y268N) in at least two runs. This further supports the effect of the mutations on substrate binding.

Contact maps do not differentiate between interaction types such as hydrophobic, hydrogen bonds or vdWs. Interatomic interactions predicted by DynaMut (refer to Figure 3) were used to differentiate interaction types calculated by contact maps in Figure 8, Figures S6–S9. The interatomic interaction changes shown in Figure 3 include: Tyr268-Pro260, Tyr268-Leu265 and Tyr268-Leu271. Out of the three interactions, our contact map findings of the reduction of hydrophobic interaction (Tyr268-Pro260) were in good agreement with the DynaMut predictions in Figure 3. The vdW interactions Tyr268-Leu265 and Tyr268-Leu271 from the predictions in Figure 3, were observed to be present 100% of simulation time as seen in Figure S9. Interactions in the mutant proteins between residue 268 and Pro260, Leu265 and Leu271 though not observed in Figure 3, were present in Figure S9. This varied observation could be because prediction analysis was performed on static structure as opposed to weighted residue contact map which samples vast data over MD simulation.

Looking at protein function, we noted that key residues Val140, Val259, Pro260 and Phe264 were crucial in substrate binding, and experienced either reduction or loss of contacts in mutant systems (Y268C and Y268N), (Y268C, Y268S and Y268N), Y268N and Y268S, respectively. Structurally, Val259 and Pro260 are in a loop which is flexible; thus, the interactions in mutant systems are likely to be destabilized. Moreover, in terms of which mutation has the greatest effect on residue-residue interactions, the mutant systems were ranked based on the number of residues that have reduced or lost contacts in comparison to the total number of residues forming contacts in the WT system. The ranking is as follows: Y268S reporting 20% while mutant systems Y268C and Y268N reporting 30% each showing the two latter systems had the greatest effect on residue contact. Residue contacts Ile141, Ile258, Glu261 and Cys334 that were maintained > 90% of trajectory time in mutant systems were not present in WT.

The point mutations (Y268C/N/S) occur at the same residue position. However, the molecular size and their physicochemical difference on residue-residue interactions is the focus of our study. Residue Tyr268 has been shown to be important in maintaining the proteins activity, especially in substrate binding, due to its aromatic side chain that supports substrate stability [75]. At this residue position, a large hydrophobic residue or one that contains an aromatic side chain would be essential in maintaining optimal enzyme activity. In this study, all three mutations lack the aromatic side chain. However, in terms of size, both Ser and Cys residues are small as compared to the Asn residue. Thus, we would expect a severe mutation effect in Y268S and Y268C as opposed to the Y268N mutant system with regards to interatomic interactions. Based on the number of contacts as well as the percentage of contact reduction as shown in Figure 8 and Figure S9, Y268S and Y268C were the most affected and likely to cause higher levels of resistance than the Y268C mutation. This corresponds to the prevalence of cytochrome b mutations in the following order Y268S, Y268C, Y268N previously reported [7,29,30,76]. Overall, these results support previous findings where in the presence of mutations, these key hydrophobic interactions at the protein’s active site were reduced [33].

Overall, four residues out of a total of 20 contacting residues, representing ~20% of the residues forming contacts in WT, were observed in all the mutant systems. Structurally, the four contacting residues (Leu265, Phe267, Ala269 and Leu271) are in the alpha helices of the protein, which are reported to be conserved thus more stable and able to form sufficient stabilizing interactions [77].

### 2.10. Effect of Mutation on Protein Communication and Residue Accessibility

Mutant effects on the global protein’s residue accessibility and communication were then investigated using dynamic residue network (DRN) analysis. Calculations were performed by extracting the last 100 ns (250—350 ns time scale) of the simulation and processing using MD-TASK [78]. This portion represented the equilibrated section of the MD simulation. DRN analysis was performed to identify important residues involved in intra-protein communication in the WT and mutant systems, as applied previously to other cases [41,79,80]. *L* is a metric that highlights the accessibility of a residue to all other residues, while *BC* is indicative of the centrality (hence importance) of a residue to protein communication. Residues associated with a lower *L* are more accessible than those with higher *L* values. Residues associated with higher *BC* values are more important to have function compared to those with a lower *BC*.

**Figure 9:**
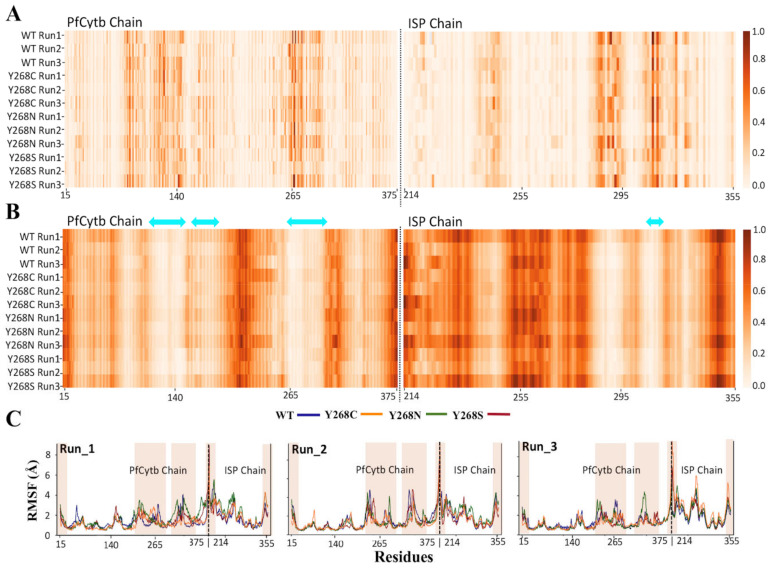
Dynamic residue network (DRN) analysis. Heat map representation of normalized values of (**A**) *Betweenness centrality (BC)* and (**B**) *Average shortest path (L)* for PfCytb and ISP chains. *L* values were correlated with root mean square fluctuation (RMSF) which is plotted in (**C**) where the WT (wild-type) and mutant systems Y268C, Y268N and Y268S are represented by the blue, orange, green and red lines, respectively. (**C**). Black dotted lines in RMSF plots show an intersection between the two chains. Two-sided cyan arrow on the *L* heat map represents active site residues. Each protein system had triplicate runs presented in (**A**–**C**).

Residue *BC* and *L* values across the last 10,001 frames of the MD simulation were averaged and normalized between 0 and 1 for all triplicate runs for all protein systems in both holo and ATQ-bound complexes. The results are presented in Figure 9 and Table S6 for holo and ATQ bound systems, respectively. The data gives an overall view of regions of significance in the different protein systems. A pairwise comparison of *BC* and *L* values among WT and mutant systems was performed. Using the Pearson method, a pairwise correlation value of > 0.90 was obtained across all the protein systems for *BC* calculations showing that the systems are highly correlated. Similarly, a correlation value of >0.72 was obtained across all the protein systems for *L* calculations. DRN analysis showed that there were several key residues identified to be system specific and deemed functionally important based on their high *BC* values. Worthy of attention are the PfCytb-ISP active site residues that are crucial in enhancing binding and stability [18,33].

In Figure 9A and Table 2, we compare high *BC* values in the holo mutant systems using WT as the reference. Here, we observed the following residues having high *BC* values across all the systems: 85, 120, 123, 124, 140, 141, 160, 260–265, 268, 271 and 272. Out of these, active site residues included: Val120, Phe123, Val124, Val140, Ile141, Pro260, Glu261, Phe264, Tyr268 and Leu271. These residues showed high average normalized *BC* (> 0.75), representing the upper quartile of the data in all systems.

Comparison of the high *BC* values in both holo and ATQ-bound proteins are displayed as a heat map in Figure S12. In PfCytb-ISP holo structure, Table 2 indicates residues having high *BC* values in the triplicate runs. Here, residue Trp262 was observed to have high *BC* values in all runs across WT and mutant systems. WT systems reported high *BC* values in residues Trp262 and His320 across the triplicate runs. Phe123, Val259, Phe264 and His301 residues were reported in at least two runs. In the mutant systems, Y268C reported residues Leu271 and His320 common across all runs and Phe123, Ile141, Glu261 and Cys268 common residues in at least two runs. Y268N reported residues Phe264, Asn268 and His320 to be common across all runs and Pro260, Glu261 and His301 residues to be common in at least two runs. Lastly, the Y268S mutant system reported residue His320 to be common across triplicate runs and Phe123, Val140, Ile141 and Phe264, residues to be common in at least two runs.

Likewise, DRN analysis on PfCytb-ISP ATQ-bound structure also reported high *BC* values as shown in Table S6. We noted that active site residue Phe264 and metal coordinating residues (His301 and His320) were reported in at least two runs in WT and Y268C, Y268N and Y268S mutant systems. In individual systems, residues Phe169, Val259, Phe264, Cys299, His301 and His320 reported high connectivity in at least two runs of the WT system. Similarly, all the mutant systems reported various active site residues as having high *BC* values. Specifically, these residues include: (Phe123, Val124, Tyr_263, Phe264, Leu271, His301 and His320), (Met133, Tyr263, Phe264, Asn268, His301 and His320) and (Phe123, Gly137, Val140, Tyr263, Phe264, Aer268, His301 and His320) observed in mutant systems Y268C, Y268N and Y268S, respectively. Overall, despite the different active site residues indicated by each system, all systems in both holo and ATQ-bound proteins had a similar number of residues, indicating high connectivity in the catalytic site of the protein.

Residues Trp262 and Tyr263, located in the mitochondrial PEWY (Pro260-Glu261-Trp262-Tyr263) conserved motif also known as the Qo motif [81] were noted to have high *BC* value. The PEWY motif found on a loop region of this protein has been reported to maintain the function of Qo active site. From the results, residue Trp262 was common among the triplicate runs in WT systems, whereas residue Tyr263 was common to all mutant systems across triplicate runs. These results indicate the importance of the conserved motif in protein function and thus supports previous findings [81]. Additionally, we noted that ATQ-bound protein highlighted residues His301 and His320 that coordinate the [2FE-2S] metal cluster present in the ISP chain in not only the WT but also mutant systems. According to our findings, when ATQ drug binds, the protein function is likely to be maintained even in the presence of mutations. This is a positive step towards drug discovery in identification of novel inhibitors that can withstand ATQ resistance caused by mutations [18].

**Figure 10:**
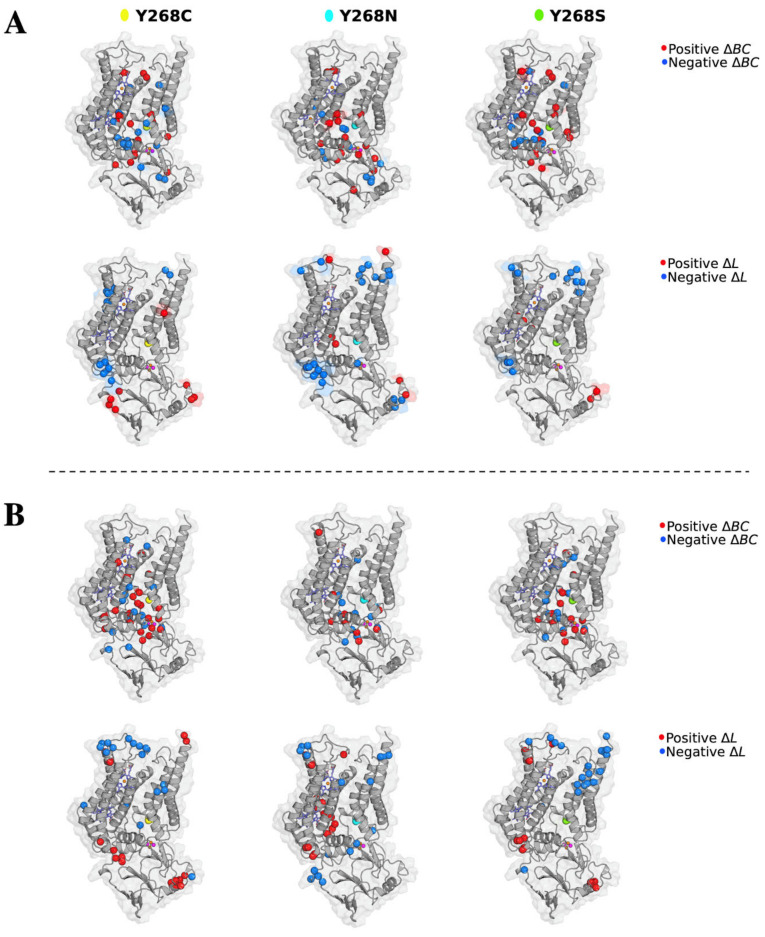
Effect of the three mutations Y268C, Y268N and Y268S on the protein intra-communication in the holo protein. (**A**) PfCytb-ISP holo protein (**B**) PfCytb-ISP ATQ-bound protein. Residues exhibiting a significant change in average *betweenness centrality* (Δ*BC*) are mapped onto the structure. The change in Δ*BC* was calculated as WT (wild-type) — mutant. The red spheres therefore represent residues with positive Δ*BC* values, i.e., residues that lost centrality in the mutant model relative to the WT. The blue spheres represent residues with negative Δ*BC* values, i.e., residues that gained more centrality in the mutant models relative to the WT (**B**) Residues exhibiting a significant change in average accessibility (Δ*L*) are mapped onto the structure. Change in Δ*L* was calculated as WT — mutant. The red spheres therefore represent residues with positive Δ*L*, i.e., residues that increased accessibility in the mutant model relative to the WT. The blue spheres represent residues with negative Δ*L* values, i.e., residues that decreased accessibility in the mutant model relative to the WT.

To investigate the effect of these mutations to protein communication, changes to average *BC* (Δ*BC*: WT—Mutant) was calculated. The data assumed a normal distribution as shown in Figure S13, thus a threshold value of ± 2 standard deviations (SD) of the Δ*BC* values was used for each holo and ATQ-bound PfCytb-ISP protein system. This was performed for all the triplicate runs and data is presented in Tables S7 and S8 where common significant residues with *BC* greater or less than two standard deviations were reported. Positive Δ*BC* signifies a decrease in residue connectivity while negative Δ*BC* signifies increased residue connectivity which in turn enhances residue participation in protein communication in the mutant system. Most of these residues were located either in the protein active site of PfCytb-ISP protein structure or within its proximity. The significant residues in both holo and ATQ-bound systems were mapped as shown in Figure 10. A closer look at the comparison across the triplicate runs with a focus on +Δ*BC* values highlights residue Trp262 which was observed to lose centrality in all three mutant systems. Additionally, residues Ile119 in Y268C; Phe123, Val140 and Ile141 in Y268N; as well as lle119 and Val259 in Y268S were observed to lose importance in mutant systems (Refer to Table S7).

Analysis of the ATQ-bound protein showed both decreased and increased connectivity due to the presence of mutations as shown in Table S8. In at least two runs, residues Val259 in Y268C and His301 in Y268S were observed to likely affect connectivity. Conversely, residues Phe264 in Y268C, His192 and His320 in Y268N and, Phe123 and Tyr263 in Y268S systems were reported to gain centrality in residue connectivity that agrees with the high *BC* values reported in these systems.

Important to note is that residue Trp262 and Tyr263, which form a part of the PEWY motif, are near point mutation Y268C/N/S. Point mutations at this region are likely to affect substrate binding. Literature also confirms the effect of mutations in close proximity to the PEWY motif, which impairs the redox process [82]. In general, a similar pattern has been observed in terms of the residues with highest Δ*BC* across all the mutant systems, suggesting a possible detrimental effect on residue communication because of point mutations. Also, the proper function of the protein would to be destabilized in the mutant system seeing as the active site residues are losing importance as compared to the WT. In addition, some active site residues were also observed to gain centrality in all mutant systems in at least two runs. These include, Phe123; Pro260 and Phe264; and Tyr263 in the Y268C, Y268N and Y268S mutant systems, respectively, which could indicate compensatory mutant mechanisms. These findings agree with those of residue contact map interactions (refer to Section 2.9) where residues Val140 and Val259 were observed to have reduced and a loss of contacts across the mutant systems. This provides more insights on the effect of these mutations on protein function and ATQ activity.

Concerning residue accessibility, the DRN which represents the protein in the form of nodes (residues) with their connections (edges) was analyzed. Here, the residues that participate in the residue network within the protein were reported to have a lower *L* value (< 0.25). These residues include: 116, 119, 122–123, 126, 137, 140–144, 260–269 and 271–272. Out of these residues, > 95% are active site residues thus easily accessible to participate in substrate binding. Also, the metal coordinating residues were observed to have lower *L* values including: His78, His92, His173, His187, Cys299, His301, Cys317 and His320. In this regard, Figure 9B and Figure 10B clearly highlight such lower *L* values to correspond to the Qo substrate binding region of the protein as well as regions of functional importance. A similar pattern was also observed across the WT and mutant systems which could possibly be useful in drug discovery. This is because the residues important to protein function are more accessible for substrate binding even in the presence of mutation. Thus, inhibitors can possibly be identified that can withstand mutation effects. Also, a direct correlation between the regions with highest average *L* and those having high residue fluctuations has been observed [83] as shown in Figure 9B and shaded regions in Figure 9C. These high fluctuations are because of loop regions that are known to be flexible in nature. In summary, *L* analysis correlates with RMSF in which residues located in the loop regions of the protein as shown in Figure 9C and Figure 10B exhibited a decrease in residue accessibility.

High (+) Δ*L* values mean that the mutant systems were reported to have increased residue reachability in both holo and ATQ bound proteins. Significant residues showing major changes to *L* value are detailed in Tables S9 and S10. Residues such as Cys214, Trp221 and Ile222 were observed to have low (−) Δ*L* values in the holo protein. Specifically, Trp221 was observed across Y268C and Y268N mutant systems in all triplicate runs while Ile222 in at least two runs of the same systems. Also, residue Cys214 was inconsistently reported across all mutant systems in at least two runs. In terms of residue location, Cys214 is one of the interfacing residues located between the two chains. Residues Trp221 and Ile222 are in the loop regions as well as at the heterodimer interface of the protein. This significantly showed the possible effect of growing distance between residues, making them less accessible. In essence, these point mutations have the potential to destabilize the protein structure, especially at the heterodimer interface. Conversely, in the ATQ-bound protein, the mutant systems exhibit high (+) Δ*L* values in the interfacing residues Cys214, Lys215 and Phe219 in at least two runs in all mutant systems. This shows the residues at the heterodimer interface are more accessible once ATQ drug binds.

## 3. Materials and Methods

### 3.1. Software

AMBER and AmberTools19, University of California, San Francisco, USA; AutoDock4.2 software, The Scripps Research Insitute, San Deigo, USA; Discovery Studio 2016, Dassault Systemes BIOVIA, San Deigo, USA; GaussView 5.0.9, Carnegie Mellon University Gaussian, Conneticut, USA; GROMACS v2018.2, University of Groningen, Uppsala Sweden; R v3.6.1, R Core Team, Vienna, Austria; Maestro v12.5, Schrödinger, New York, USA; MD-TASK v1.0.1, Research Unit in Bioinformatics (RUBi), Rhodes University, Makhanda, South Africa; PyMOL Molecular Graphics System; v1.7.2.1 Schrödinger, New York, USA.

### 3.2. Input Structure: Protein Modeling

The protein sequences of PfCytb (UniProt accession: Q02768) and ISP (UniProt accession: Q8IL75) subunits were downloaded from the Universal Protein Resources (UniProt) [84]. Crystal structures of the mentioned protein subunits were not available and therefore models were constructed using I-TASSER [42,43,44]. The program uses both threading and *ab initio* techniques. It first identifies proteins from the PDB through multiple threading approaches using LOMETS. Structural alignment is then performed on these high-scoring templates for refinement purposes, then predicted models are calculated based on protein structures common across the templates. In addition, besides structural similarity, protein function is also considered in the structural analogs identified to the predicted final model. Thus, PDB ID: 1PP9 was used as the principal template in guiding the modeling process. Full-length atomic models were then generated by iterative template-based fragment assembly simulations [44].

To obtain a complex between the two predicted models (PfCytb and ISP), the individually modeled subunits were separately superimposed onto corresponding chains of homologous *Bos taurus* cytbc_1_ complex (PDB ID: 1PP9) using PyMOL [85]. This was done by first superimposing the modeled PfCytb subunit onto the crystal structure after which the ISP chain was then superimposed onto the resulting complex to form the final PfCytb-ISP complex. This ensured that the model was in a similar orientation to that of the template, which would enhance the ease of transferring all the coordinates of the prosthetic groups. The coordinates for b-type hemes and [2FE-2S] cluster prosthetic groups on x-ray crystal structure 1PP9 were then directly transferred onto the modeled complex. This approach was performed, as the I-TASSER web server does not model cofactors. The overall structural quality of the PfCytb-ISP complex was further evaluated using z-DOPE score RAMPAGE [86] and PROCHECK [55].

### 3.3. Mutant Protein Modeling

The reported point mutations (Y268C, Y268N and Y268S) conferring PfCytb resistance to the ATQ drug were obtained from literature [7,29,30]. The crystal structures of the mutants (Y268C, Y268N and Y268S) were not available within the PDB; therefore, mutant protein models were generated using the wild-type (WT) protein as template. This was performed by mutating the Tyr268 to the appropriate mutant using Discovery Studio 2016 [87] followed by structure minimization. Model quality for each mutant model was then evaluated and validated according to that of the WT protein.

### 3.4. Structure Preparation: Protonation of the Protein

To the WT and mutant protein models, the H++ web server [88,89] was used to protonate all non-titratable and titratable residue groups to the correct state [90,91,92]. The titratable groups included residues such as His [93], Asp, Glu, Arg, Lys, Cys, Tyr residues. Each protein contained a total of 111 titratable sites. Protonation for the protein structure was performed at the following physiological conditions of a neutral pH of 7.0, salinity of 0.15 M and an external and internal dielectric of 80 and 10, respectively. At the post-protonation step, AmberTools19 software incorporated in H++ is used to generate coordinate and topology files. The topology files for the protonated PDB files were then inspected using Maestro software [94]. It was noted that the H++ server had distorted the orientation of the distal His301 residue that coordinates one of the metal ions within the [2FE-2S] cluster. To solve the problem, the His301 residue was manually rotated to bring it closer to and coordinate the Fe^2+^ atom. The resultant structure was then minimized using GROMACS v2018.2 software to relieve steric clashes [95]. Post-minimization, the protonation states for all other metal coordinating residues were then evaluated for correct protonation and manually adjusted if necessary. This was performed, as incorrect protonation states could influence metal ion parameterization, protein behavior and dynamics during MD simulation.

### 3.5. Force Field Parameter Calculations

The generation of force field parameters for the metal cofactors (heme bL and [2FE-2S] cluster) was performed using the WT protein as a template. Following structure preparation, Metal center parameter builder (MCPB) [96], was used to generate input files for Gaussian09 quantum mechanical (QM) calculations. A cut-off distance of 2.8 Å was applied to describe all metal site interactions. MCPB.py [96] is a script that comes bundled together with AmberTools19 that allows for simple generation of force field parameters [71]. Though force field parameters have been previously developed for the heme and [2FE-2S] cluster, re-parameterization was performed to allow development of parameters in a format that is simple to use and modify for further analysis.

To the MCPB generated input files, GaussView 5.0.9, a compatible graphical user interface and part of Gaussian software packages [97], was used to visualize all files to ensure that all cofactors and coordination residues were detected and had been factored in for QM calculations. All QM calculations were carried out using B3LYP/6-31G* level of theory Gaussian09 software [98]. The basis set was applied as it has been shown to produce accurate results using inexpensive computational resources [99]. The 6-31G* is a split-valence double-zeta plus polarization basis set, particularly suitable for organic molecules. The polarization functions on non-hydrogen atoms are indicated by the * (asterisk). The ** is used when the polarization is further added to hydrogen atoms such as in 6-31G**. First, geometry optimization of the molecular structures was performed and post optimization, evidence of any broken bonds and changes to coordination or geometry distortion was investigated using GaussView 5.0.9. These could potentially affect the reliability of the parameters and their ability to maintain the cofactors within the protein. This was done by visualizing the log files to check the precise coordination environment [69]. All QM calculations were performed at the CHPC (Center for High Performance Computing) cluster using 240 CPU cores and 15,000 MB of memory.

After QM calculations, the Seminario method incorporated in the MCPB was used to derive bond lengths, angles and respective force constants upon ensuring accurate geometry optimization. The Seminario method calculates these parameters from the submatrices of the Cartesian Hessian matrix [100]. Finally, to describe possible electrostatic residue interactions occurring between cofactors and coordination residues, restrained electrostatic potential (RESP) charges were derived on the protein model using MCPB. On the large model (containing not only the interacting residues but also surrounding atoms), QM was used for calculating the RESP charges. This was performed using a Merz-Kollman RESP fitting algorithm which factors in all the atoms that interact with the metal coordinating residues [101]. Regarding spin states, these describe the central metal’s d electrons where its potential spin configurations can either be high-spin or low-spin. For transition metal complexes involving metal clusters, DFT calculations in this study employed the framework of Hohenberg-kohn theorems (H-K) using non-degenerate electronic ground states [102].

The energy profiles were then calculated using AMBER energy function where force field parameters (force constants, atomic charges and distances) for bonds, angles, and dihedrals were factored according to Equation (1):

(1) $$V\left(r^{N}\right)=\sum_{bonds}^{n}k_{b}{\left(l-l_{0}\right)}^{2}+\sum_{angles}^{n}k_{a}{\left(\theta -\theta_{0}\right)}^{2}+\sum_{dihedrals}^{n}\;\sum_{n}^{i}\frac{1}{2}V_{n}\left[1+cos\left(n\omega -\gamma \right)\right]+\;\sum_{j=1}^{N-1}\sum_{i=J+1}^{N}f_{ij}\left\{\in_{ij}\left[{\left(\frac{r_{0ij}}{r_{ij}}\right)}^{12}-2{\left(\frac{r_{0ij}}{r_{ij}}\right)}^{6}\right]+\frac{qiqj}{4\pi \epsilon_{0}r_{ij}}\right\}$$

where the first three terms (bonds, angles and dihedrals) express harmonic oscillator calculations/approximations while the last term describes the nonbonded interactions. Bond and angle terms $l_{0}$ and $\theta_{0}$ are equilibrium bond lengths and bond angles while *ka* and *kb* denote the bond stretching and angle bending force constants. In the dihedral torsional term, *Vn* represents the energy barrier of torsional motion; *n*, γ and ω stand for periodicity, phase shift and dihedral angle, respectively [103,104].

Parameter calculations were performed for the heme bL and [2FE-2S] cluster in the WT model, after which the heme parameters were inferred onto the second heme (heme bH). This was done as the two heme structures have similar coordination geometry, both forming bonds with two His residues on each side of the metal. The residues that participate in metal bond formation; heme bH axial ligands are His78 and His173 while for heme bL axial ligands are His92 and His187. The derived Fe^2+^ parameters for the PfCytb-ISP protein were validated using MD simulation, compared to crystal structure (PDB ID: 1PP9). Also, the entire parameter set was inferred to all mutant protein models (Y268C, Y268N and Y268S) prior to MD simulations.

### 3.6. Retrieval of ATQ Structure and Molecular Docking Approach

The ATQ 2D chemical structure was retrieved from the PubChem database [105]. Prior to docking, the drug compound was converted to 3D then protonated to ensure accuracy in docking scores and poses generated. Both protein and ligand structures were prepared using prepare_receptor4.py and prepare_receptor4.py protocols in MGLToolsPckgs of AutoDock4.2 software, respectively [106]. Using the Gasteiger-Huckel method in AutoDock tools, the partial atomic changes of the ligands were assigned. Thereafter, molecular docking experiments were performed targeting the Qo site of the PfCytb-ISP WT protein model using AutoDock Vina v1.1.2 [107]. The docking parameters used were as follows: a cubic box of grid points 48, 47 and 54 along x, y, z dimensions with a grid spacing of 1 Å. For the docked results, an RMSD cut-off of 2 Å was utilized to obtain accurate predictions of the bound structures.

A total of 10 orientations were generated for the ATQ ligand, out of which ranking was performed based on binding energy and RMSD value. The ligand conformation with the lowest-docking energy score and RMSD was selected. Thereafter, the docked ligand coordinates from the WT model were transferred to the mutant proteins (Y268C, Y268N and Y268S) for modeling MD simulations.

### 3.7. POPC:POPE Lipid Bilayer Construction and Topology Generation

Prior to MD simulations, since PfCytb-ISP is an integral membrane protein, Packmol-memgen was used to build the IMM and insert the respective proteins in order to simulate WT and mutant protein dynamics accurately [39]. The IMM membrane is composed of PC, PE, CL, PA and PI [9]. Of these, PC, PE and PA lipid residues are available for membrane construction using Packmol-memgen. PC and PE make up approximately 60–70% of the membrane each depending on the organism, with PC being more abundant. PA is, however, not present in some organisms. As a result, the proteins were set up to be embedded in a POPC:POPE membrane of lipid ratio 1.3:1, respectively. The membrane density was set to allow 15 Å distance between the protein and the boundary of the potential box. The number of lipids to be constructed was then determined using the area per lipid and the desired lipid leaflet area. Packmol-memgen was also set to solvate the protein-membrane complex using the TIP3P water model [108] to a thickness of 17.5 Å above and below each leaflet. After construction of the protein-membrane complex, protein orientation within the membrane was validated with that of 1PP9 in the OPM/PPM server [109]. Visual inspection of the embedded protein was also performed to ensure that sufficient membrane lipids had been packed between the protein and box boundary to accurately investigate membrane effects. The potential presence of water within the membrane was also investigated. Figure S14 shows an image of the constructed membrane, and additional details are provided in Table S11.

Lipid residues composed of acyl chains Palmitoyl and Oleoyl accounting for a total of 327 lipids each while the head groups composed of PC and PE having 185 and 142 lipid17 residues respectively; more details for each system are provided as follows in Table S11. The protein embedded in the membrane is illustrated in Figure S14.

The constructed protein membrane systems were loaded into the LEaP program for the generation of MD simulation topology and coordinate files. The AMBER ff14SB and Lipid17 force field were selected to provide parameters for the amino acid and lipid residues [110]. The AMBER ff14SB was further extended using the previously calculated metal parameters to incorporate the metal cofactors and coordinating residues. Although Packmol-memgen solvated the protein membrane system, it was not set to neutralize it. Therefore, respective numbers of counter ions were incorporated as shown in Table S11 to neutralize the system prior to energy minimization. Finally, LEaP was set to generate the topology in a simulation box size of 105 × 105 × 127 Å. AMBER topology files produced include: coordinate (.inpcrd) file having coordinates for all atoms in the system, the parameter topology (.prmtop) file containing atomic coordinates and all the parameters were used to calculate forces and energies.

### 3.8. Molecular Dynamics Simulation of Protein-Membrane Systems

After topology generation, the GPU version of the PMEMD program was used to prepare the system for MD simulation [71]. Firstly, the protein-membrane system was minimized using a combination of the steepest descent algorithm and the conjugate gradient method for a maximum of 20,000 steps. These included: 10,000 steps of steepest descent minimization followed by 10,000 steps of conjugate gradient optimization. This was performed at a non-bonded cut-off distance of 10 Å. After minimization, temperature equilibration was then performed in two phases where the system was brought up to a maximum temperature of 310K using Langevin dynamics at a collision frequency of 1.0 per ps and the canonical (constant T) ensemble. In the first heating phase, the system temperature was gradually brought up to 310 K over 500 ps. Afterwards, the temperature for the system was maintained at 310 K for a further 500 ps, and then pressure equilibrated to 1.0 bar using anisotropic (x-, y-, z-) pressure scaling and the Berendsen barostat for 1 ns with a pressure relaxation time of 2.0 ps. During temperature and pressure equilibration, the membrane lipid residues numbering between 512 and 1501, were held fixed using a force constant of 10 kcal mol^−1^ Å^2^. All bonds involving hydrogen were also constrained using the SHAKE algorithm.

Prior to the production run, a hold step was performed to equilibrate the systems periodic boundary dimensions considering MD was to be performed using the PMEMD GPU code. Each WT and mutant protein membrane system was subjected to 10 separate equilibrations of 500 ps each (5 ns in total) with the system temperature and barostat set to 310 K and 1 bar, respectively. Due to the changing nature of the system’s periodic boundary conditions, the skinnb value of the system was set to 5 Å during every restart. Finally, a 350 ns MD production run was performed at the Center for High-Performance Computing (CHPC) in Cape Town, South Africa on 10 CPU cores and one Nvidia Tesla v100 GPU. The system was set to use a 0.002 ps timestep with coordinates written to file every 10 ps.

The production run trajectory was visualized using VMD [111] and post processed with CPPTRAJ [112] to remove solvent, membrane and counter ions from the protein-membrane complex. The CPPTRAJ program was further used to calculate root mean squared deviation (RMSD), root mean square fluctuations (RMSF) and the radius of gyration (Rg). All statistical tests were performed using Mann-Whitney U-test using R v3.6.1 where a *p*-value of < 0.05 was considered significant.

### 3.9. Dynamic Residue Network Calculations

To evaluate intra-protein communications and changes occurring in the protein residues across the MD simulation, MD-TASK was utilized [78]. The software utilized a reduced trajectory consisting of only Cβ atoms for each protein (Cα for glycine) system across all frames. The change to average *betweenness centrality* (*BC*) and average shortest path (*L*) of protein residues were calculated over the last 100 ns of the simulation representing the equilibrated phase (250–350 ns). Average *BC* identifies residues most important in communication within a protein while average *L* of protein residues is indicative of their relative accessibility. For *BC* and *L* calculations, a cut-off distance of 6.7 Å was used as the residue-residue contact distance. The average *BC* was calculated to identify the residues in the network that are important in protein communication while the average shortest path provided the density of all shortest paths among all node pairs. For easier comparison of *BC* and *L* between WT and mutant proteins, results were normalized onto a scale of 0–1 using unity-based normalization. Delta *BC* and *L* were then calculated by subtracting average values in the mutant from the WT protein. Using the normalized output data, the significant residues within ± 2 SD were mapped onto the protein structure using PyMOL Molecular Graphics System v1.7.2.1 [85]. Residue contact maps were also calculated for the equilibrated phase of the trajectory (last 100 ns) for all systems (WT, Y268C, Y268N and Y268S), based on pairwise residue distance between all Cβ (or Cα for glycine residue). A contact cut-off distance of 6.7 Å and an iteration step size of 100 were applied to obtain all residue-residue contacts around active site and point mutation residue positions. Calculated contacts were then represented as the percentage fraction of the selected MD frames. All DRN calculations were performed via online platform MDM-TASK-web available at (https://mdmtaskweb.rubi.ru.ac.za/; accessed on 1 February 2021) [113].

### 3.10. Principal Component Analysis

To investigate the conformational entropy of the proteins, principal component analysis (PCA) of the WT and mutant proteins was performed according to Sanyanga et al. [80] using CPPTRAJ [112]. Briefly, an RMS best-fit of the trajectories was applied to an average structure followed by the calculation of the coordinate covariance matrix for the Cα and Cβ atoms. The matrix was then diagonalized to obtain respective eigenvectors and eigenvalues. To the eigenvectors, protein coordinates were then projected. Normalization was then performed on the primary and secondary projections followed by plotting them against each other to obtain a plot of PC1 versus PC2. Associated free energy of the structural conformations was also calculated and plotted together with PC1 and PC2.

A detailed explanation of the computational tools used in the study is provided in the supplementary file (Table S12).

## 4. Conclusions

PfCytb-ISP protein complex is an essential part of the plasmodial cytochrome bc_1_ complex. Activity of this protein has been effectively inhibited using ATQ drug. However, spread of drug resistant parasites has compromised the efficacy of the drug and presented a challenge in the efforts to control and prevent malaria. In this study, we provide insights on the effect of mutations on PfCytb-ISP dynamics using in silico approaches. To promote efficiency in future studies such as virtual screening and drug design, we highlight the importance of accurate description of MD simulations which largely depend on the use of accurate force field parameters as well as membrane simulations in describing protein dynamics. Firstly, the derived Fe^+2^ parameters describing the b-type hemes and [2FE-2S] cluster were validated to hold and maintain the metal ions in the coordination sphere. These parameters were validated through all atom MD simulations of PfCytb-ISP protein embedded in a phospholipid bilayer and useful for not only PfCytb-ISP protein but also other metalloproteins which share similar coordination environments. Secondly, from MD simulations, DRN analysis and residue contact maps, we have observed that all the three mutations (Y268C, Y268N and Y268S) destabilize the protein system by directly affecting the Qo substrate binding site. Previous studies have associated ATQ drug resistance with the Y268S point mutation. Further clinical studies have linked ATQ treatment failures not only to Y268S point mutation but also to Y268C and Y268N mutations. Our results agree with these findings and additionally highlight that point mutations Y268C and Y268N share a similar resistance mechanism to Y268S mutation. With the clear ATQ drug resistance mechanisms [18,33,57], our study findings focusing on the local residue level confirm the effect of mutations on protein function which mostly affect the PfCytb-ISP active site residues. The computational findings highlight the parasite’s ability to evade drug action. As such, further experimental studies are needed to support these findings. On the other hand, increases to active site residue communication were observed even in the presence of mutations. This suggests a fitness cost of the parasites to available drugs resulting in parasites’ ability to evade drug action mechanism due to the compensatory nature of the mutations. The result of this is widespread drug resistance due to the fitness cost. The active site key residues with increased residue connectivity could be explored in drug design to circumvent the development of drug resistance by the parasite. Ultimately, the implication of the prediction of drug resistance by all three point mutations is important in the search for novel inhibitors that can withstand all these mutations. This is highly important in the current search for novel *P. falciparum* cytbc_1_ complex inhibitors.

## Figures and Tables

**Table 1:** Prediction on the effect of mutations (Y268C, Y268N and Y268S) on PfCytb-ISP protein stability using VAPOR and DynaMut. The tools define ΔΔG > 0 as stabilizing and ΔΔG < 0 as destabilizing.

	Mutations		
**Prediction Values**			
	**Y268C**	**Y268N**	**Y268S**
**DynaMut** (ΔΔG)	−1.06	1.45	1.49
**I-Mutant** (ΔΔG)	−0.82	−0.98	−1.31
**MuPro** (ΔΔG)	−0.91	−1.17	−1.37
**Stability**	Decrease	Decrease	Decrease
**PhD-SNP**	Disease	-	Disease

**Table 2:** Protein residues having high normalized *Betweenness Centrality (BC)* values in holo WT and three mutant systems across the triplicate runs; + sign indicate significant high values. Active site residues are shown in bold. The * and # suffixes indicate common residues across 3 runs and 3 runs, respectively. (A threshold value of ± 2 standard deviations (SD) of the *BC* values was used for each system.

*Betweenness Centrality* (*BC*); Significant Residues				
Protein Systems		RUN_1	RUN_2	RUN_3
**WT**	+	**PfCytb Chain:** Ser_83, Ile_93, Ala_122, Phe_123 *, I**le_141**, Asn_143, **Trp_262 #**, Leu_265, **Tyr_268**, Lys_272, Leu_290, Leu_291 **ISP Chain:** Gly_297, Ile_298, His_301 *, Leu_302, **His_320 #**, Ser_322, Ile_330	**PfCytb Chain:** Val_85, Thr_89, Met_102, Phe_115, Phe_118, **Ile_119**, Ala_122, Val_124, Ile_258, Val_259 *, Trp_262 #, Phe_264 *, Leu_265, Lys_272, Leu_291 **ISP Chain:** Gly_297, Ile_298, Gly_303, **His_320 #**, Ser_322, Ile_330	**PfCytb Chain:** Ser_83, Val_85, Phe_86, Thr_89, Leu_94, Phe_118, Ala_122, Phe_123 *, Thr_139, Ile_141, Val_259 *, Trp_262 #, Phe_264 *, Leu_265, **Leu_271**, Lys_272, Lys_277, Leu_290, Leu_294 **ISP Chain:** Gly_297, Ile_298, His_301 *, Val_305, **His_320 #**, Ser_322, Ile_330
**Y268C**	+	**PfCytb Chain:** Thr_80, Val_85, Phe_86, Thr_89, Leu_112, Phe_118, Ala_122, **Phe_123**, Ser_134, Thr_139, **Ile_141**, Thr_160, Val_161, **Glu_261**, **Trp_262**, Leu_265, Cys_268 *, Leu_271 #, Lys_272 **ISP Chain:** Lys_252, Cys_304, **His_320**, Ser_322	**PfCytb Chain:** Val_85, Phe_86, Thr_89, Ile_117, Phe_118, Val_120, Ala_122, **Phe_123**, Ala_138, Thr_139, Val_140, Ile_141, Tyr_263 *, Phe_264 *, Met_270, **Leu_271#**, Lys_272, Leu_281, Leu_290, Ile_320 **ISP Chain:** Gly_297, Ile_298, Gly_303, Cys_304, **His_320**, Ser_322, Ile_330	**PfCytb Chain:** Val_85, Phe_86, Thr_89, Leu_112, Phe_118, Thr_121, **Val_124**, Thr_160, Thr_165, **Ile_258**, **Pro_260, Glu_261**, **Tyr_263** *, Phe_264 *, Cys_268 *, Leu_271 #, Lys_272, Lys_277, Ile_349 **ISP Chain:** Lys_252, His_301, Leu_302, **His_320**, Ser_322
**Y268N**	+	**PfCytb Chain:** Val_85, Phe_86, Thr_89, Ile_93, **Val_124**, Phe_169, Ser_241, His_242, Thr_254, **Tyr_263** #, **Phe_264 #, Asn_268 #**, Lys_272, Lys_277, Leu_288, Leu_291 **ISP Chain:** Gly_297, Ile_298, His_301 *, Leu_302, Gly_303, **His_320 #**, Ser_322, Ile_330	**PfCytb Chain:** Val_85, Phe_86, Thr_89, Leu_112, Ile_117, Phe_118, I**le_119, Gly_137**, Thr_160, Leu_172, Leu_176, His_242, Thr_254, Pro_260 *, Glu_261 *, Tyr_263 #, **Phe_264 #, Asn_268 #,** Lys_272, ISP Chain: Cys_299 *, Gly_303, **His_320 #**, Ser_322, Ile_330, Pro_334	**PfCytb Chain:** Ser_83, Val_85, Phe_86, Leu_112, Ile_117, **Met_133**, Thr_139, Leu_172, Leu_176, Pro_260 *, Glu_261 *, Tyr_263 #, P**he_264 #, Asn_268 #**, **Leu_271**, Lys_272, Lys_277, Leu_288, Leu_291 **ISP Chain:** Gly_297, Ile_298, Cys_299 *, His_301 *, Gly_303, **His_320 #**, Ser_322, Pro_334
**Y268S**	+	**PfCytb Chain:** Ile_35, Val_85, Phe_86, Thr_89, Trp_108, Ile_117, **Val_120, Phe_123, Val_140, Ile_141, Thr_142, Leu_145**, Thr_160, **Val_259**, **Pro_260, Glu_261,** Leu_265, **Leu_271** **ISP Chain:** Lys_252, Ile_298, Cys_304, Pro_318, **His_320 #**, Ser_322, His_323	**PfCytb Chain:** Leu_31, Val_85, Phe_86, Thr_89, Leu_112, Ile_117, Phe_118, Thr_121, Ala_122, **Phe_123, Val_124,** Val_127, **Trp_136, Gly_137**, Thr_160, Ser_241, Tyr_263 *, Phe_264 *, Lys_272, Ile_320 **ISP Chain:** Gly_297, Ile_298, Val_305, Ala_307, **His_320 #**, Ser_322, Ile_330	**PfCytb Chain:** Ser_83, Phe_86, Thr_89, Arg_95, Phe_118, Thr_121, Ala_138, Thr_139, **Val_140, Ile_141, Phe_169**, Tyr_263 *, Phe_264 *, Ser_268, Lys_272, Ile_349 **ISP Chain:** Ile_298, **Cys_299,** Cys_304, Val_305, **His_320#**, Gly_321, Ser_322, Ile_330, Pro_334

## Data Availability

All the data is presented in this article and in the supplementary files.

## Supplementary Materials

Supplementary materials can be found at https://www.mdpi.com/1422-0067/22/4/2138/s1.

## Abbreviations

ATP	Adenosine triphosphate
ATQ	Atovaquone
*BC*	*Betweenness centrality*
DFT	Density functional theory
DRN	Dynamic residue network
ETC	Electron transport chain
IMM	Inner mitochondrial membrane
*L*	Average shortest path
MM	Molecular mechanics
MD	Molecular dynamics
MEP	Molecular electrostatic potential
PCA	Principal component analysis
POPC	1-palmitoyl-2-oleoyl-sn-glycero-3-phosphocholine
POPE	1-palmitoyl-2-oleoyl-sn-glycero-3-phosphoethanolamine
QM	Quantum mechanics
RESP	Restrained electrostatic potential
RMSD	Root mean square deviation
RMSF	Root mean square fluctuation
Rg	Radius of gyration
VAPOR	Variant Analysis Portal
WT	Wild-type
