# Supplementary material for: Decoding the Molecular Effects of Atovaquone Linked Resistant Mutations on Plasmodium falciparum Cytb-ISP Complex in the Phospholipid Bilayer Membrane
Source: Int J Mol Sci. 2021 Feb 21;22(4):2138. doi: 10.3390/ijms22042138 (PMC7926518; doi:10.3390/ijms22042138)
Supplement: Supplementary file 1 [file ijms-22-02138-s001.pdf]

Supplementary data: Decoding the molecular effects of atovaquone linked resistant mutations on *Plasmodium falciparum* Cytb-ISP complex in the phospholipid bilayer membrane

Lorna Chebon-Bore [1,†], Taremekedzwa Allan Sanyanga [1,†], Colleen Varaidzo Manyumwa [1], Afrah Khairallah [1] and Özlem Tastan Bishop [1,*]

[1] Research Unit in Bioinformatics (RUBi), Department of Biochemistry and Microbiology, Rhodes University, Grahamstown 6140, South Africa; lornajemosop@gmail.com, asanyanga@gmail.com, colleen.manyumwa06@gmail.com, afrahkhairalla@gmail.com

† Equally contributed first authorship

* Correspondence: o.tastanbishop@ru.ac.za (Ö.T.B); Tel.: +27-46-603-8072 (Ö.T.B.)

Table S1. Computed pKa (1/2) values for all ionizable groups (side chains) that are coordinated in the metal center of the PfCytb-ISP protein complex (Internal dielectric of 10).

**Deprotonation state(ε)**		**Deprotonation state(δ/SG)**	
**Site**	**pKa values**	**Site**	**pKa values**
His78	4.7	His301	<0.0
His92	<0.0	His320	1.2
His173	2.6	Cys299	>12.0
His187	4.5	Cys317	6.6
C-Term	4.7	N-Term	5.1
Total charge at pH 7.0	4	Isoelectric point	8.64

Table S2. Titratable residues and pKa values in the PfCytb-ISP protein complex

Residue	pK (int)	pKa_(1/2)	Residue	pK (int)	pKa_(1/2)	Residue	pK (int)	pKa_(1/2)
TYR-2	11.160	>12.000	CYS-310	10.463	10.189	ASP-473	6.271	<0.000
CYS-4	11.058	>12.000	TYR-313	12.143	>12.000	ARG-477	11.789	>12.000
TYR-14	11.330	>12.000	CYS-320	9.510	9.361	ARG-479	11.287	>12.000
ARG-35	12.118	>12.000	ASP-325	3.876	2.734	GLU-488	5.623	1.812
TYR-36	14.652	>12.000	TYR-330	10.027	10.072	TYR-492	12.303	>12.000
ASP-39	3.584	2.076	ARG-332	12.244	>12.000	GLU-493	4.930	3.562
TYR-42	10.429	10.636	CYS-340	9.619	9.393	ASP-496	4.608	3.001
TYR-44	11.718	>12.000	HIS-348	5.819	4.919	GLU-497	4.451	2.924
TYR-45	10.057	10.160	TYR-349	10.003	9.952	LYS-501	10.182	>12.000
HIS-49	6.067	6.658	ARG-350	13.576	>12.000	CTASN-361	5.192	4.681
ARG-52	12.409	>12.000	ARG-351	11.992	>12.000	ASP-473	6.271	<0.000
GLU-53	4.706	2.437	HIS-353	2.975	<0.000	ARG-477	11.789	>12.000
CYS-59	9.050	8.825	TYR-354	9.973	9.929	ARG-479	11.287	>12.000
ARG-61	10.818	>12.000	ASP-355	5.352	3.234			
TYR-62	11.376	11.657	TYR-356	9.834	10.132			
HID-64	6.239	4.746	NTASN-1	6.908	5.029			
TYR-76	11.774	>12.000	NT CYS-362	8.126	7.935			
HIS-78	4.667	<0.000	LYS-363	10.201	10.024			
ARG-81	10.864	>12.000	HIS-366	6.496	5.836			
TYR-85	12.449	10.926	LYS-372	10.203	10.941			
TYR-87	12.909	>12.000	ASP-373	3.921	2.741			
TYR-89	10.095	9.951	GLU-381	5.545	5.074			
TYR-112	10.773	>12.000	ASP-383	3.867	1.768			
TYR-121	12.813	>12.000	ARG-385	12.067	>12.000			
CYS-142	11.459	11.532	GLU-391	4.212	3.079			
TYR-145	12.226	9.966	HIS-392	5.725	4.122			
ASP-149	4.262	0.551	LYS-396	10.095	10.531			
LYS-153	9.714	>12.000	ARG-398	12.462	>12.000			
ARG-154	12.600	>12.000	LYS-400	9.934	>12.000			

HIS-159 5.792 2.608	LYS-405 10.189 >12.000	
CYS-168 9.273 9.182	HID-406 7.143 7.022	
HID-173 6.246 4.463	ARG-407 12.854 >12.000	
HIS-178 6.066 5.662	GLU-410 5.172 4.492	
HIS-180 6.564 5.832	ASP-411 4.318 <0.000	
TYR-188 11.056 10.993	ARG-414 12.066 >12.000	
ASP-189 3.965 2.709	LYS-416 9.838 >12.000	
LYS-193 9.978 10.823	GLU-417 5.511 2.963	
TYR-197 9.328 9.343	ASP-418 5.434 0.436	
ASP-204 4.730 3.064	ASP-419 3.755 2.706	
LYS-206 10.360 10.876	LYS-420 10.393 11.725	
HID-228 4.530 4.797	ARG-426 12.923 >12.000	
ASP-230 3.544 2.014	ASP-427 3.237 1.368	
TYR-238 12.779 >12.000	ASP-431 7.220 <0.000	
GLU-247 6.434 3.415	ASP-433 3.770 3.138	
TYR-249 14.109 >12.000	ARG-434 12.729 >12.000	
LYS-258 11.439 >12.000	LYS-437 9.841 >12.000	
LYS-263 10.896 11.739	GLU-439 5.317 3.630	
GLU-282 4.497 2.110	CYS-447 8.775 >12.000	
ARG-284 12.469 >12.000	HIS-449 3.925 <0.000	
LYS-293 10.144 10.054	TYR-460 10.496 11.004	
ARG-299 13.129 >12.000	TYR-463 14.864 >12.000	
ASP-300 4.115 2.877	CYS-465 7.745 6.648	
TYR-301 10.462 11.616	HID-468 5.114 1.246	

Table S3. Partial atomic charges before (formal charge) and after charge calculations restrained electrostatic potential (RESP) charges derived using gaussian09 software using B3LYP/6-31G* basic set at DFT level of theory. NB: RESP charges for only the coordinating residues are presented below.

Atoms	Initial atomic Charge	RESP charge
	**BEFORE**	**AFTER**
FE1 (HEME BL)	2.0000	-0.0093
FE1 (HEME BH)	2.0000	-0.0093
FE2 [2FE-2S]	2.0000	0.5906
FE3 [2FE-2S]	2.0000	0.7323
SG1 CYS [2FE-2S]	-2.0000	-0.8579
SG2 CYS [2FE-2S]	-2.0000	-0.6260

Table S4. Parameter and coordinate files for both Heme & [2FE-2S) cluster model B3LYP/6-31G* containing optimized coordinates and derived atomic partial charges (AMBER parameter files included also):

(a) Protoporphyrin IX containing FE; Heme (HEM.mol2)
(b) Heme proximal histidine ligand (His78.mol2)
(c) Heme distal histidine ligand (His173.mol2)
(d) [2FE-2S] proximal histidine ligand (His301.mol2)
(e) [2FE-2S] distal histidine ligand (His320.mol2)
(f) [2FE-2S] proximal cysteine ligand (Cys299.mol2)
(g) [2FE-2S] distal cysteine ligand (Cys317.mol2)
(h) An AMBER parameter (*.frcmod) file

(a) Protoporphyrin IX Heme octa-coordinate and its derived atomic charges.

HEM.mol2

```
@<TRIPOS>MOLECULE
HEM
   72    76     1     0     0
SMALL
RESP Charge


@<TRIPOS>ATOM
      1 CHA        56.5750   66.3360   63.4640 ce         1 HEM      -0.053091
      2 CHB        60.1540   67.7760   66.4260 ce         1 HEM      -0.142569
      3 CHC        57.4820   66.6740   70.2620 ce         1 HEM      -0.109350
      4 CHD        53.9860   65.2570   67.2620 ce         1 HEM      -0.139521
      5 C1A        57.8200   66.8080   63.9350 cc         1 HEM      -0.074815
      6 C2A        58.9610   67.3250   63.1290 cc         1 HEM      -0.097541
      7 C3A        59.9930   67.6700   63.9390 cd         1 HEM       0.042618
      8 C4A        59.4420   67.4480   65.2850 cd         1 HEM      -0.055780
      9 CMA        61.3210   68.2390   63.5190 c3         1 HEM      -0.105332
     10 CAA        58.9710   67.6000   61.6440 c3         1 HEM      -0.022660
     11 CBA        58.1140   68.8300   61.4130 c3         1 HEM      -0.041797
     12 CGA        57.7700   69.0520   59.9490 c          1 HEM       0.541097
     13 O1A        58.6220   69.5690   59.1970 o          1 HEM      -0.620901
     14 O2A        56.6400   68.7090   59.5600 o          1 HEM      -0.413672
     15 C1B        59.7360   67.6250   67.7480 cc         1 HEM      -0.099086
     16 C2B        60.5620   67.8750   68.9500 cc         1 HEM       0.060434
     17 C3B        59.8020   67.5880   70.0560 cd         1 HEM      -0.027679
     18 C4B        58.5100   67.1300   69.5160 cd         1 HEM      -0.054395
     19 CMB        61.9990   68.4120   68.9750 c3         1 HEM      -0.068779
     20 CAB        60.1620   67.7100   71.3790 cf         1 HEM      -0.095735
     21 CBB        61.4930   67.3490   72.0660 c2         1 HEM      -0.533903
     22 C1C        56.3060   66.0990   69.7970 cc         1 HEM      -0.019937
     23 C2C        55.2140   65.4930   70.6100 cc         1 HEM       0.051407
     24 C3C        54.2770   65.0860   69.6910 cd         1 HEM      -0.018292
     25 C4C        54.7650   65.4670   68.4040 cd         1 HEM      -0.059841
     26 CMC        55.1110   65.3790   72.1150 c3         1 HEM      -0.139834
     27 CAC        53.0820   64.4560   69.8290 cf         1 HEM      -0.148847
     28 CBC        52.5290   63.7950   71.1010 c2         1 HEM      -0.560753
     29 C1D        54.3020   65.4620   65.9720 cc         1 HEM      -0.053396
     30 C2D        53.4620   65.0580   64.8450 cc         1 HEM       0.040491
     31 C3D        54.1720   65.4390   63.7570 cd         1 HEM      -0.066318
     32 C4D        55.4860   65.9830   64.2460 cd         1 HEM      -0.055737
     33 CMD        52.1540   64.2910   64.9280 c3         1 HEM      -0.101229
     34 CAD        53.8050   65.2490   62.2930 c3         1 HEM      -0.019091
     35 CBD        53.1580   66.4690   61.6420 c3         1 HEM      -0.006871
     36 CGD        54.1670   67.5250   61.2390 c          1 HEM       0.611618
     37 O1D        54.0410   68.6920   61.6790 o          1 HEM      -0.659831
     38 O2D        55.0900   67.1730   60.4790 o          1 HEM      -0.516704
     39 NA         58.1540   66.9310   65.2830 Y3         1 HEM      -0.094695
     40 NB         58.4810   67.1140   68.0970 Y4         1 HEM      -0.011203
     41 NC         55.9850   66.0330   68.4530 Y5         1 HEM       0.040970
     42 ND         55.5520   65.9930   65.6200 Y6         1 HEM      -0.059847
     43 HMA1       61.5890   67.8460   62.6730 hc         1 HEM       0.040745
     44 HMA2       61.9880   68.0370   64.1940 hc         1 HEM       0.040745
     45 HMA3       61.2440   69.2010   63.4190 hc         1 HEM       0.040745
     46 HMB1       62.1430   68.9140   69.7920 hc         1 HEM       0.030254
```

```
     47 HMB2        62.1410   68.9920   68.2110 hc        1 HEM       0.030254
     48 HMB3        62.6230   67.6700   68.9380 hc        1 HEM       0.030254
     49 HMC1        54.1770   65.3950   72.3780 hc        1 HEM       0.051120
     50 HMC2        55.5760   66.1230   72.5290 hc        1 HEM       0.051120
     51 HMC3        55.5140   64.5460   72.4050 hc        1 HEM       0.051120
     52 HMD1        51.5830   64.5440   64.1860 hc        1 HEM       0.037984
     53 HMD2        51.7090   64.4990   65.7640 hc        1 HEM       0.037984
     54 HMD3        52.3340   63.3390   64.8870 hc        1 HEM       0.037984
     55 HBB1        61.6010   67.4930   73.0190 ha        1 HEM       0.197664
     56 HBB2        62.2220   66.9780   71.5450 ha        1 HEM       0.197664
     57 HBC1        51.6570   63.3700   71.0870 ha        1 HEM       0.187462
     58 HBC2        53.0520   63.8020   71.9180 ha        1 HEM       0.187462
     59 HBA1        58.5800   69.6100   61.7520 hc        1 HEM       0.046864
     60 HBA2        57.2930   68.7460   61.9230 hc        1 HEM       0.046864
     61 HAA1        58.6210   66.8410   61.1520 hc        1 HEM       0.044499
     62 HAA2        59.8760   67.7490   61.3290 hc        1 HEM       0.044499
     63 HBD1        52.5180   66.8580   62.2580 hc        1 HEM      -0.005904
     64 HBD2        52.6610   66.1870   60.8580 hc        1 HEM      -0.005904
     65 HAD1        53.1980   64.4960   62.2180 hc        1 HEM       0.022799
     66 HAD2        54.6060   65.0180   61.7970 hc        1 HEM       0.022799
     67 HHA         56.4760   66.2540   62.5430 ha        1 HEM       0.159129
     68 HHB         61.0020   68.1360   66.2970 ha        1 HEM       0.148531
     69 HHC         57.5700   66.7530   71.1840 ha        1 HEM       0.110019
     70 HHD         53.1290   64.9300   67.4170 ha        1 HEM       0.111705
     71 HAB         59.4790   68.0760   71.9630 ha        1 HEM       0.129768
     72 HAC         52.5180   64.4190   69.0410 ha        1 HEM       0.165550
@<TRIPOS>BOND
     1     1     5 1
     2     1    32 1
     3     1    67 1
     4     2     8 1
     5     2    15 1
     6     2    68 1
     7     3    18 1
     8     3    22 1
     9     3    69 1
    10     4    25 1
    11     4    29 1
    12     4    70 1
    13     5     6 1
    14     5    39 1
    15     6     7 1
    16     6    10 1
    17     7     8 1
    18     7     9 1
    19     8    39 1
    20     9    43 1
    21     9    44 1
    22     9    45 1
    23    10    11 1
    24    10    61 1
    25    10    62 1
    26    11    12 1
    27    11    59 1
    28    11    60 1
    29    12    13 1
    30    12    14 1
    31    15    16 1
    32    15    40 1
    33    16    17 1
    34    16    19 1
    35    17    18 1
```

```
    36   17   20 1
    37   18   40 1
    38   19   46 1
    39   19   47 1
    40   19   48 1
    41   20   21 1
    42   20   71 1
    43   21   55 1
    44   21   56 1
    45   22   23 1
    46   22   41 1
    47   23   24 1
    48   23   26 1
    49   24   25 1
    50   24   27 1
    51   25   41 1
    52   26   49 1
    53   26   50 1
    54   26   51 1
    55   27   28 1
    56   27   72 1
    57   28   57 1
    58   28   58 1
    59   29   30 1
    60   29   42 1
    61   30   31 1
    62   30   33 1
    63   31   32 1
    64   31   34 1
    65   32   42 1
    66   33   52 1
    67   33   53 1
    68   33   54 1
    69   34   35 1
    70   34   65 1
    71   34   66 1
    72   35   36 1
    73   35   63 1
    74   35   64 1
    75   36   37 1
    76   36   38 1
@<TRIPOS>SUBSTRUCTURE
     1 HEM        1 TEMP              0 ****  ****    0 ROOT
```

(b) HEME proximal histidine ligand coordinates and atomic charges

<u>His78.mol2</u>

```
@<TRIPOS>MOLECULE
HD3
   17    17     1     0     0
SMALL
RESP Charge


@<TRIPOS>ATOM
      1 N          55.2010   74.0280   65.4910 N         1 HD3     -0.415700
      2 H          54.4690   73.8790   65.0650 H         1 HD3      0.235973
      3 CA         55.9850   72.8320   65.7720 CX        1 HD3      0.018800
      4 HA         56.2340   72.8430   66.7090 H1        1 HD3      0.056284
      5 CB         55.1420   71.5730   65.5280 CT        1 HD3      0.082654
      6 HB2        54.2330   71.7390   65.8230 HC        1 HD3      0.035925
      7 HB3        55.1030   71.3980   64.5750 HC        1 HD3      0.035925
      8 CG         55.6730   70.3480   66.2320 CC        1 HD3     -0.172662
      9 ND1        55.4930   70.0840   67.5890 NA        1 HD3     -0.192872
     10 HD1        55.1160   70.5950   68.1690 H         1 HD3      0.329990
     11 CE1        56.0260   68.8700   67.8090 CR        1 HD3     -0.034138
     12 HE1        56.0320   68.4340   68.6300 H5        1 HD3      0.083349
     13 NE2        56.5490   68.3760   66.6750 Y1        1 HD3     -0.014437
     14 CD2        56.3410   69.2980   65.6670 CV        1 HD3     -0.005823
     15 HD2        56.6040   69.2190   64.7780 H4        1 HD3      0.159459
     16 C          57.2920   72.8160   64.9870 C         1 HD3      0.597300
     17 O          58.3470   72.7770   65.6090 O         1 HD3     -0.567900
@<TRIPOS>BOND
     1    1    2 1
     2    1    3 1
     3    3    4 1
     4    3    5 1
     5    3   16 1
     6    5    6 1
     7    5    7 1
     8    5    8 1
     9    8    9 1
    10    8   14 1
    11    9   10 1
    12    9   11 1
    13   11   12 1
    14   11   13 1
    15   13   14 1
    16   14   15 1
    17   16   17 1
@<TRIPOS>SUBSTRUCTURE
     1 HD3         1 TEMP              0 ****  ****    0 ROOT
```

(c) HEME distal histidine ligand coordinates and atomic charges

<u>His173.mol2</u>

```
@<TRIPOS>MOLECULE
HD4
   17    17     1     0     0
SMALL
RESP Charge


@<TRIPOS>ATOM
      1 N          58.4080   59.0740   68.6040 N          1 HD4     -0.415700
      2 H          58.1220   58.7250   67.8720 H          1 HD4      0.228349
      3 CA         58.3640   60.5340   68.5970 CX         1 HD4      0.018800
      4 HA         59.2630   60.8110   68.8350 H1         1 HD4      0.074433
      5 CB         58.0100   61.1030   67.2150 CT         1 HD4      0.048962
      6 HB2        58.6540   60.7720   66.5700 HC         1 HD4      0.034786
      7 HB3        57.1420   60.7590   66.9520 HC         1 HD4      0.034786
      8 CG         57.9760   62.6190   67.1310 CC         1 HD4     -0.116106
      9 ND1        59.0670   63.4650   67.3120 NA         1 HD4     -0.223384
     10 HD1        59.8780   63.2330   67.4780 H          1 HD4      0.336786
     11 CE1        58.6010   64.7120   67.1760 CR         1 HD4     -0.009107
     12 HE1        59.1130   65.4830   67.2680 H5         1 HD4      0.136437
     13 NE2        57.2840   64.6910   66.8870 Y2         1 HD4      0.028113
     14 CD2        56.8650   63.3740   66.8620 CV         1 HD4     -0.124850
     15 HD2        56.0040   63.0640   66.6960 H4         1 HD4      0.152567
     16 C          57.3820   61.0980   69.6230 C          1 HD4      0.597300
     17 O          57.6140   62.1550   70.2020 O          1 HD4     -0.567900
@<TRIPOS>BOND
     1    1    2 1
     2    1    3 1
     3    3    4 1
     4    3    5 1
     5    3   16 1
     6    5    6 1
     7    5    7 1
     8    5    8 1
     9    8    9 1
    10    8   14 1
    11    9   10 1
    12    9   11 1
    13   11   12 1
    14   11   13 1
    15   13   14 1
    16   14   15 1
    17   16   17 1
@<TRIPOS>SUBSTRUCTURE
     1 HD4         1 TEMP              0 ****  ****    0 ROOT
```

(d) [2FE-2S] cluster proximal histidine ligand coordinates and atomic charges

His320.mol2

```
@<TRIPOS>MOLECULE
HE2
   17    17     1     0     0
SMALL
RESP Charge


@<TRIPOS>ATOM
      1 N          76.6730   60.4870   51.0020 N         1 HE2     -0.415700
      2 H          76.4680   59.6910   50.7480 H         1 HE2      0.263227
      3 CA         78.0730   60.6710   51.2760 CX        1 HE2     -0.058100
      4 HA         78.0620   61.5640   51.6540 H1        1 HE2      0.078755
      5 CB         78.6600   59.8310   52.3950 CT        1 HE2     -0.005376
      6 HB2        78.7920   58.9110   52.1170 HC        1 HE2      0.059976
      7 HB3        79.5240   60.1690   52.6770 HC        1 HE2      0.059976
      8 CG         77.6500   59.9330   53.4880 CC        1 HE2      0.130370
      9 ND1        76.5060   59.1620   53.4310 Z4        1 HE2     -0.382996
     10 CE1        75.7900   59.4580   54.5470 CR        1 HE2     -0.021464
     11 HE1        74.9690   59.0730   54.7530 H5        1 HE2      0.155613
     12 NE2        76.3940   60.3570   55.3070 NA        1 HE2     -0.148993
     13 HE2        76.1180   60.6840   56.0530 H         1 HE2      0.327309
     14 CD2        77.5690   60.6560   54.6360 CW        1 HE2     -0.266746
     15 HD2        78.2090   61.2630   54.9290 H4        1 HE2      0.185024
     16 C          78.9640   60.7970   50.0760 C         1 HE2      0.597300
     17 O          80.1690   60.5950   50.2030 O         1 HE2     -0.567900
@<TRIPOS>BOND
     1    1    2 1
     2    1    3 1
     3    3    4 1
     4    3    5 1
     5    3   16 1
     6    5    6 1
     7    5    7 1
     8    5    8 1
     9    8    9 1
    10    8   14 1
    11    9   10 1
    12   10   11 1
    13   10   12 1
    14   12   13 1
    15   12   14 1
    16   14   15 1
    17   16   17 1
@<TRIPOS>SUBSTRUCTURE
     1 HE2         1 TEMP              0 ****  ****    0 ROOT
```

(e) [2FE-2S] cluster distal histidine ligand coordinates and atomic charges

<u>His301.mol2</u>

```
@<TRIPOS>MOLECULE
HE1
   17    17     1     0     0
SMALL
RESP Charge


@<TRIPOS>ATOM
      1 N           76.9840   52.1080   51.4450 N          1 HE1      -0.415700
      2 H           76.9200   52.5150   50.5930 H          1 HE1       0.325555
      3 CA          77.2400   52.6660   52.4610 CX         1 HE1      -0.058100
      4 HA          78.0560   52.1390   52.9350 H1         1 HE1       0.038670
      5 CB          77.5370   54.0500   51.9540 CT         1 HE1       0.080741
      6 HB2         78.3760   54.0220   51.2350 HC         1 HE1      -0.027235
      7 HB3         76.6270   54.3380   51.4260 HC         1 HE1      -0.027235
      8 CG          77.7700   55.1340   52.9230 CC         1 HE1       0.082017
      9 ND1         77.1630   55.4180   54.1410 Z2         1 HE1      -0.454443
     10 CE1         77.7440   56.4410   54.6630 CR         1 HE1       0.089071
     11 HE1         77.5230   56.9120   55.6000 H5         1 HE1       0.115415
     12 NE2         78.5580   56.9320   53.6630 NA         1 HE1      -0.082129
     13 HE2         79.3430   57.6740   53.8690 H          1 HE1       0.212982
     14 CD2         78.5950   56.0730   52.6800 CW         1 HE1      -0.079996
     15 HD2         79.1690   56.1450   51.8220 H4         1 HE1       0.190476
     16 C           76.0740   52.6280   53.3580 C          1 HE1       0.597300
     17 O           75.7280   51.6550   53.9980 O          1 HE1      -0.567900
@<TRIPOS>BOND
     1    1    2 1
     2    1    3 1
     3    3    4 1
     4    3    5 1
     5    3   16 1
     6    5    6 1
     7    5    7 1
     8    5    8 1
     9    8    9 1
    10    8   14 1
    11    9   10 1
    12   10   11 1
    13   10   12 1
    14   12   13 1
    15   12   14 1
    16   14   15 1
    17   16   17 1
@<TRIPOS>SUBSTRUCTURE
     1 HE1          1 TEMP              0 ****  ****    0 ROOT
```

(f) [2FE-2S] cluster proximal cysteine ligand coordinates and atomic charges

Cys317.mol2

```
@<TRIPOS>MOLECULE
CX1
   10     9     1     0     0
SMALL
RESP Charge


@<TRIPOS>ATOM
      1 N          73.2370   51.9060   47.9180 N          1 CX1      -0.415700
      2 H          72.7060   52.5760   48.0110 H          1 CX1       0.267345
      3 CA         74.6370   52.1740   48.1270 CX         1 CX1       0.042900
      4 HA         75.0900   51.7480   47.3830 H1         1 CX1       0.028004
      5 CB         75.1250   53.6340   47.9420 2C         1 CX1       0.147741
      6 HB2        76.0690   53.6020   47.7190 H1         1 CX1      -0.056234
      7 HB3        74.6640   54.0020   47.1720 H1         1 CX1      -0.056234
      8 SG         74.9250   54.8020   49.3090 Y7         1 CX1      -0.276273
      9 C          75.0760   51.5540   49.4170 C          1 CX1       0.597300
     10 O          74.4540   51.7120   50.4670 O          1 CX1      -0.567900
@<TRIPOS>BOND
     1    1    2 1
     2    1    3 1
     3    3    4 1
     4    3    5 1
     5    3    9 1
     6    5    6 1
     7    5    7 1
     8    5    8 1
     9    9   10 1
@<TRIPOS>SUBSTRUCTURE
     1 CX1         1 TEMP              0 ****  ****    0 ROOT
```

(g) [2FE-2S] cluster distal cysteine ligand coordinates and atomic charges

<u>Cys299.mol2</u>

```
@<TRIPOS>MOLECULE
CX2
   10     9     1     0     0
SMALL
RESP Charge


@<TRIPOS>ATOM
      1 N          76.1250   58.5360   44.7370 N          1 CX2      -0.415700
      2 H          76.9360   58.3270   44.9330 H          1 CX2       0.247913
      3 CA         75.2200   58.5520   45.8390 CX         1 CX2       0.042900
      4 HA         74.2890   58.5170   45.5690 H1         1 CX2       0.028474
      5 CB         75.4370   57.3240   46.6870 2C         1 CX2      -0.023435
      6 HB2        75.4190   56.5340   46.1240 H1         1 CX2       0.098355
      7 HB3        76.3170   57.3620   47.0940 H1         1 CX2       0.098355
      8 SG         74.1830   57.1740   47.9760 Y8         1 CX2      -0.454266
      9 C          75.4600   59.8410   46.5610 C          1 CX2       0.597300
     10 O          76.5310   60.0950   47.1120 O          1 CX2      -0.567900
@<TRIPOS>BOND
     1    1    2 1
     2    1    3 1
     3    3    4 1
     4    3    5 1
     5    3    9 1
     6    5    6 1
     7    5    7 1
     8    5    8 1
     9    9   10 1
@<TRIPOS>SUBSTRUCTURE
     1 CX2         1 TEMP              0 ****  ****    0 ROOT
```

(h) An AMBER parameter (*.frcmod) file

```
REMARK GOES HERE, THIS FILE IS GENERATED BY MCPB.PY
MASS
M1 55.85                             Fe ion
M2 55.85                             Fe ion
M3 55.85                             Fe ion
Y1 14.01         0.530              sp2 N in 5 memb.ring w/LP (HIS,ADE,GUA)
Y2 14.01         0.530              sp2 N in 5 memb.ring w/LP (HIS,ADE,GUA)
Y7 32.06         2.900              S in disulfide linkage,pol:JPC,102,2399,98
Z2 14.01         0.530              sp2 N in 5 memb.ring w/LP (HIS,ADE,GUA)
Y8 32.06         2.900              S in disulfide linkage,pol:JPC,102,2399,98
Z4 14.01         0.530              sp2 N in 5 memb.ring w/LP (HIS,ADE,GUA)
Y3 14.01         0.530              Sp2 N in non-pure aromatic systems, identical
Y4 14.01         0.530              Sp2 N in non-pure aromatic systems, identical
Y5 14.01         0.530              Sp2 N in non-pure aromatic systems, identical
Y6 14.01         0.530              Sp2 N in non-pure aromatic systems, identical
Z1 32.060        2.900              same as s
Z3 32.060        2.900              same as s

BOND
M2-Z1  139.7    2.1207      Created by Seminario method using MCPB.py
M2-Z3  125.7    2.1429      Created by Seminario method using MCPB.py
M3-Z1   52.8    2.3568      Created by Seminario method using MCPB.py
M3-Z3   76.5    2.2768      Created by Seminario method using MCPB.py
Y1-M1   42.7    2.0205      Created by Seminario method using MCPB.py
Y2-M1   43.0    2.0146      Created by Seminario method using MCPB.py
Y3-M1   55.9    2.0073      Created by Seminario method using MCPB.py
Y4-M1   44.2    2.0251      Created by Seminario method using MCPB.py
Y5-M1   46.2    2.0276      Created by Seminario method using MCPB.py
Y6-M1   64.2    2.0069      Created by Seminario method using MCPB.py
Y7-M2  131.0    2.1410      Created by Seminario method using MCPB.py
Y8-M2  118.6    2.1544      Created by Seminario method using MCPB.py
Z2-M3   32.8    2.2053      Created by Seminario method using MCPB.py
Z4-M3   74.0    1.9660      Created by Seminario method using MCPB.py
2C-Y7  227.0    1.8100
2C-Y8  227.0    1.8100
CC-Z2  410.0    1.394       JCC,7,(1986),230; HIS
CC-Z4  410.0    1.394       JCC,7,(1986),230; HIS
CR-Y1  488.0    1.335       JCC,7,(1986),230; HIS
CR-Y2  488.0    1.335       JCC,7,(1986),230; HIS
Y1-CV  410.0    1.394       JCC,7,(1986),230; HIS
Y2-CV  410.0    1.394       JCC,7,(1986),230; HIS
Z2-CR  488.0    1.335       JCC,7,(1986),230; HIS
Z4-CR  488.0    1.335       JCC,7,(1986),230; HIS
cc-Y3  525.4    1.3172          SOURCE3_SOURCE5    4612    0.0083
cc-Y4  525.4    1.3172          SOURCE3_SOURCE5    4612    0.0083
cc-Y5  525.4    1.3172          SOURCE3_SOURCE5    4612    0.0083
cc-Y6  525.4    1.3172          SOURCE3_SOURCE5    4612    0.0083
cd-Y3  441.1    1.3694          SOURCE1_SOURCE5    2269    0.0086
cd-Y4  441.1    1.3694          SOURCE1_SOURCE5    2269    0.0086
cd-Y5  441.1    1.3694          SOURCE1_SOURCE5    2269    0.0086
cd-Y6  441.1    1.3694          SOURCE1_SOURCE5    2269    0.0086

ANGLE
2C-SG1-FE2  113.72     105.40    Created by Seminario method using MCPB.py
2C-SG2-FE2  114.44     108.29    Created by Seminario method using MCPB.py
```

CC-ND1-FE3	27.74	75.99	Created by Seminario method using MCPB.py
CC-NDI-FE3	111.09	131.96	Created by Seminario method using MCPB.py
CR-NE2-FE1	104.90	125.70	Created by Seminario method using MCPB.py
CR-NE2-FE1	116.43	125.74	Created by Seminario method using MCPB.py
FE1-NE2-CV	100.14	127.65	Created by Seminario method using MCPB.py
FE1-NE2-CV	116.63	127.90	Created by Seminario method using MCPB.py
FE2-S1-FE3	47.73	82.71	Created by Seminario method using MCPB.py
FE2-S2-FE3	59.04	84.16	Created by Seminario method using MCPB.py
FE3-ND1-CR	0.00	64.27	Created by Seminario method using MCPB.py
FE3-ND1-CR	113.94	120.57	Created by Seminario method using MCPB.py
NE2-FE1-NE2	168.21	178.29	Created by Seminario method using MCPB.py
NE2-FE1-NA	134.59	91.43	Created by Seminario method using MCPB.py
NE2-FE1-NB	139.06	91.23	Created by Seminario method using MCPB.py
NE2-FE1-NC	127.35	91.04	Created by Seminario method using MCPB.py
NE2-FE1-ND	134.33	87.49	Created by Seminario method using MCPB.py
NE2-FE1-NA	118.97	88.52	Created by Seminario method using MCPB.py
NE2-FE1-NB	130.18	90.49	Created by Seminario method using MCPB.py
NE2-FE1-NC	116.87	89.01	Created by Seminario method using MCPB.py
NE2-FE1-ND	126.00	90.80	Created by Seminario method using MCPB.py
NA-FE1-NB	148.86	90.13	Created by Seminario method using MCPB.py
NA-FE1-NC	98.29	177.53	Created by Seminario method using MCPB.py
NA-FE1-ND	141.95	90.05	Created by Seminario method using MCPB.py
NB-FE1-NC	132.38	89.77	Created by Seminario method using MCPB.py
NB-FE1-ND	120.06	178.71	Created by Seminario method using MCPB.py
NC-FE1-ND	130.63	90.11	Created by Seminario method using MCPB.py
SG1-FE2-SG2	71.89	112.81	Created by Seminario method using MCPB.py
SG1-FE2-S1	62.87	118.90	Created by Seminario method using MCPB.py
SG1-FE2-S2	49.14	102.83	Created by Seminario method using MCPB.py
SG2-FE2-S1	57.34	110.40	Created by Seminario method using MCPB.py
SG2-FE2-S2	61.01	109.01	Created by Seminario method using MCPB.py
ND1-FE3-S1	9.62	147.65	Created by Seminario method using MCPB.py
ND1-FE3-S2	40.97	97.83	Created by Seminario method using MCPB.py
ND1-FE3-ND1	23.70	114.21	Created by Seminario method using MCPB.py
S2-FE2-S1	46.25	101.55	Created by Seminario method using MCPB.py
S2-FE3-S1	93.46	90.92	Created by Seminario method using MCPB.py
ND1-FE3-S1	64.05	95.81	Created by Seminario method using MCPB.py
ND1-FE3-S2	55.17	95.03	Created by Seminario method using MCPB.py
cc-NA-FE1	144.34	127.03	Created by Seminario method using MCPB.py
cc-NB-FE1	132.43	126.78	Created by Seminario method using MCPB.py
cc-NC-FE1	139.16	127.11	Created by Seminario method using MCPB.py
cc-ND-FE1	154.01	126.94	Created by Seminario method using MCPB.py
cd-NA-FE1	138.95	127.38	Created by Seminario method using MCPB.py
cd-NB-FE1	133.72	127.36	Created by Seminario method using MCPB.py
cd-NC-FE1	138.87	126.95	Created by Seminario method using MCPB.py
cd-ND-FE1	149.99	126.74	Created by Seminario method using MCPB.py
CC-CV-Y1	70.0	120.00	AA his
CC-CV-Y2	70.0	120.00	AA his
CC-Z2-CR	70.0	117.00	AA his
CC-Z4-CR	70.0	117.00	AA his
CR-Y1-CV	70.0	117.00	AA his
CR-Y2-CV	70.0	117.00	AA his
CT-CC-Z2	70.0	120.00	AA his
CT-CC-Z4	70.0	120.00	AA his
CW-CC-Z2	70.0	120.00	AA his
CW-CC-Z4	70.0	120.00	AA his
CX-2C-SG1	50.0	114.70	
CX-2C-Y8	50.0	114.70	
NA-CR-Y1	70.0	120.00	AA his
NA-CR-Y2	70.0	120.00	AA his
Y1-CR-H5	50.0	120.00	AA his
Y1-CV-H4	50.0	120.00	AA his
Y2-CR-H5	50.0	120.00	AA his

```
Y2-CV-H4    50.0       120.00    AA his
Y3-cc-cc   71.57       112.56                 SOURCE3       141    4.2871
Y4-cc-cc   71.57       112.56                 SOURCE3       141    4.2871
Y5-cc-cc   71.57       112.56                 SOURCE3       141    4.2871
Y6-cc-cc   71.57       112.56                 SOURCE3       141    4.2871
SG1-2C-H1    50.0       109.50
Y8-2C-H1    50.0       109.50
Z2-CR-H5    50.0       120.00    AA his
Z2-CR-NA    70.0       120.00    AA his
Z4-CR-H5    50.0       120.00    AA his
Z4-CR-NA    70.0       120.00    AA his
cc-Y3-cd   71.76       105.49            CORR_SOURCE5      1810    1.9032
cc-Y4-cd   71.76       105.49            CORR_SOURCE5      1810    1.9032
cc-Y5-cd   71.76       105.49            CORR_SOURCE5      1810    1.9032
cc-Y6-cd   71.76       105.49            CORR_SOURCE5      1810    1.9032
cd-cd-Y3   67.63       121.98            CORR_SOURCE5       141    1.9633
cd-cd-Y4   67.63       121.98            CORR_SOURCE5       141    1.9633
cd-cd-Y5   67.63       121.98            CORR_SOURCE5       141    1.9633
cd-cd-Y6   67.63       121.98            CORR_SOURCE5       141    1.9633
ce-cc-Y3   68.07       121.70            CORR_SOURCE5        58    1.4179
ce-cc-Y4   68.07       121.70            CORR_SOURCE5        58    1.4179
ce-cc-Y5   68.07       121.70            CORR_SOURCE5        58    1.4179
ce-cc-Y6   68.07       121.70            CORR_SOURCE5        58    1.4179
ce-cd-Y3   68.67       123.98         SOURCE4_SOURCE5        10    2.4097
ce-cd-Y4   68.67       123.98         SOURCE4_SOURCE5        10    2.4097
ce-cd-Y5   68.67       123.98         SOURCE4_SOURCE5        10    2.4097
ce-cd-Y6   68.67       123.98         SOURCE4_SOURCE5        10    2.4097

DIHE
X -CC-Z2-X     2      4.8      180.0   2.0        JCC,7,(1986),230
X -CC-Z4-X     2      4.8      180.0   2.0        JCC,7,(1986),230
X -CR-Y1-X     2     10.0      180.0   2.0        JCC,7,(1986),230
X -CR-Y2-X     2     10.0      180.0   2.0        JCC,7,(1986),230
X -CV-Y1-X     2      4.8      180.0   2.0        JCC,7,(1986),230
X -CV-Y2-X     2      4.8      180.0   2.0        JCC,7,(1986),230
X -Z2-CR-X     2     10.0      180.0   2.0        JCC,7,(1986),230
X -Z4-CR-X     2     10.0      180.0   2.0        JCC,7,(1986),230
X -cc-Y3-X     2      9.5      180.0   2.0     statistic value from parm94
X -cc-Y4-X     2      9.5      180.0   2.0     statistic value from parm94
X -cc-Y5-X     2      9.5      180.0   2.0     statistic value from parm94
X -cc-Y6-X     2      9.5      180.0   2.0     statistic value from parm94
X -cd-Y3-X     2      9.5      180.0   2.0     statistiv value from parm94
X -cd-Y4-X     2      9.5      180.0   2.0     statistiv value from parm94
X -cd-Y5-X     2      9.5      180.0   2.0     statistiv value from parm94
X -cd-Y6-X     2      9.5      180.0   2.0     statistiv value from parm94
2C-SG1-M2-Y8   3     0.00       0.00   3.0   Treat as zero by MCPB.py
2C-SG1-M2-Z1   3     0.00       0.00   3.0   Treat as zero by MCPB.py
2C-SG1-M2-Z3   3     0.00       0.00   3.0   Treat as zero by MCPB.py
2C-Y8-M2-Z1    3     0.00       0.00   3.0   Treat as zero by MCPB.py
2C-Y8-M2-Z3    3     0.00       0.00   3.0   Treat as zero by MCPB.py
C -CX-2C-SG1   1    0.278        0.0  -4.0    C-C
C -CX-2C-SG1   1    0.323        0.0  -3.0
C -CX-2C-SG1   1    0.394      180.0  -2.0
C -CX-2C-SG1   1    0.602        0.0   1.0
C -CX-2C-Y8    1    0.278        0.0  -4.0    C-C
C -CX-2C-Y8    1    0.323        0.0  -3.0
C -CX-2C-Y8    1    0.394      180.0  -2.0
C -CX-2C-Y8    1    0.602        0.0   1.0
CC-CV-Y1-M1    3     0.00       0.00   3.0   Treat as zero by MCPB.py
CC-CV-Y2-M1    3     0.00       0.00   3.0   Treat as zero by MCPB.py
CC-Z2-M3-Z1    3     0.00       0.00   3.0   Treat as zero by MCPB.py
CC-Z2-M3-Z3    3     0.00       0.00   3.0   Treat as zero by MCPB.py
```

CC-Z2-M3-Z4	3	0.00	0.00	3.0	Treat as zero by MCPB.py
CC-Z4-M3-Z1	3	0.00	0.00	3.0	Treat as zero by MCPB.py
CC-Z4-M3-Z3	3	0.00	0.00	3.0	Treat as zero by MCPB.py
CR-Y1-M1-Y2	3	0.00	0.00	3.0	Treat as zero by MCPB.py
CR-Y1-M1-Y3	3	0.00	0.00	3.0	Treat as zero by MCPB.py
CR-Y1-M1-Y4	3	0.00	0.00	3.0	Treat as zero by MCPB.py
CR-Y1-M1-Y5	3	0.00	0.00	3.0	Treat as zero by MCPB.py
CR-Y1-M1-Y6	3	0.00	0.00	3.0	Treat as zero by MCPB.py
CR-Y2-M1-Y3	3	0.00	0.00	3.0	Treat as zero by MCPB.py
CR-Y2-M1-Y4	3	0.00	0.00	3.0	Treat as zero by MCPB.py
CR-Y2-M1-Y5	3	0.00	0.00	3.0	Treat as zero by MCPB.py
CR-Y2-M1-Y6	3	0.00	0.00	3.0	Treat as zero by MCPB.py
CT-CC-Z2-M3	3	0.00	0.00	3.0	Treat as zero by MCPB.py
CT-CC-Z4-M3	3	0.00	0.00	3.0	Treat as zero by MCPB.py
CW-CC-Z2-M3	3	0.00	0.00	3.0	Treat as zero by MCPB.py
CW-CC-Z4-M3	3	0.00	0.00	3.0	Treat as zero by MCPB.py
CX-2C-Y7-M2	3	0.00	0.00	3.0	Treat as zero by MCPB.py
CX-2C-Y8-M2	3	0.00	0.00	3.0	Treat as zero by MCPB.py
CX-CT-CC-Z2	1	0.047	180.0	-4.0	
CX-CT-CC-Z2	1	0.74	0.0	-3.0	
CX-CT-CC-Z2	1	0.204	0.0	-2.0	
CX-CT-CC-Z2	1	0.69	0.0	1.0	
CX-CT-CC-Z4	1	0.047	180.0	-4.0	
CX-CT-CC-Z4	1	0.74	0.0	-3.0	
CX-CT-CC-Z4	1	0.204	0.0	-2.0	
CX-CT-CC-Z4	1	0.69	0.0	1.0	
M1-Y1-CR-H5	3	0.00	0.00	3.0	Treat as zero by MCPB.py
M1-Y1-CV-H4	3	0.00	0.00	3.0	Treat as zero by MCPB.py
M1-Y2-CR-H5	3	0.00	0.00	3.0	Treat as zero by MCPB.py
M1-Y2-CV-H4	3	0.00	0.00	3.0	Treat as zero by MCPB.py
M1-Y3-cc-cc	3	0.00	0.00	3.0	Treat as zero by MCPB.py
M1-Y4-cc-cc	3	0.00	0.00	3.0	Treat as zero by MCPB.py
M1-Y5-cc-cc	3	0.00	0.00	3.0	Treat as zero by MCPB.py
M1-Y6-cc-cc	3	0.00	0.00	3.0	Treat as zero by MCPB.py
M2-Y7-2C-H1	3	0.00	0.00	3.0	Treat as zero by MCPB.py
M2-Y8-2C-H1	3	0.00	0.00	3.0	Treat as zero by MCPB.py
M2-Z1-M3-Z3	3	0.00	0.00	3.0	Treat as zero by MCPB.py
M2-Z3-M3-Z1	3	0.00	0.00	3.0	Treat as zero by MCPB.py
M3-Z2-CR-H5	3	0.00	0.00	3.0	Treat as zero by MCPB.py
M3-Z2-CR-NA	3	0.00	0.00	3.0	Treat as zero by MCPB.py
M3-Z3-M2-Z1	3	0.00	0.00	3.0	Treat as zero by MCPB.py
M3-Z4-CR-H5	3	0.00	0.00	3.0	Treat as zero by MCPB.py
M3-Z4-CR-NA	3	0.00	0.00	3.0	Treat as zero by MCPB.py
N -CX-2C-Y7	1	0.064	0.0	-4.0	
N -CX-2C-Y7	1	0.323	0.0	-3.0	
N -CX-2C-Y7	1	0.021	180.0	-2.0	
N -CX-2C-Y7	1	0.469	0.0	1.0	
N -CX-2C-Y8	1	0.064	0.0	-4.0	
N -CX-2C-Y8	1	0.323	0.0	-3.0	
N -CX-2C-Y8	1	0.021	180.0	-2.0	
N -CX-2C-Y8	1	0.469	0.0	1.0	
NA-CR-Y1-M1	3	0.00	0.00	3.0	Treat as zero by MCPB.py
NA-CR-Y2-M1	3	0.00	0.00	3.0	Treat as zero by MCPB.py
Y1-M1-Y2-CR	3	0.00	0.00	3.0	Treat as zero by MCPB.py
Y1-M1-Y2-CV	3	0.00	0.00	3.0	Treat as zero by MCPB.py
Y1-M1-Y3-cc	3	0.00	0.00	3.0	Treat as zero by MCPB.py
Y1-M1-Y3-cd	3	0.00	0.00	3.0	Treat as zero by MCPB.py
Y1-M1-Y4-cc	3	0.00	0.00	3.0	Treat as zero by MCPB.py
Y1-M1-Y4-cd	3	0.00	0.00	3.0	Treat as zero by MCPB.py
Y1-M1-Y5-cc	3	0.00	0.00	3.0	Treat as zero by MCPB.py
Y1-M1-Y5-cd	3	0.00	0.00	3.0	Treat as zero by MCPB.py
Y1-M1-Y6-cc	3	0.00	0.00	3.0	Treat as zero by MCPB.py

Y1-M1-Y6-cd	3	0.00	0.00	3.0	Treat as zero by MCPB.py
Y2-M1-Y1-CV	3	0.00	0.00	3.0	Treat as zero by MCPB.py
Y2-M1-Y3-cc	3	0.00	0.00	3.0	Treat as zero by MCPB.py
Y2-M1-Y3-cd	3	0.00	0.00	3.0	Treat as zero by MCPB.py
Y2-M1-Y4-cc	3	0.00	0.00	3.0	Treat as zero by MCPB.py
Y2-M1-Y4-cd	3	0.00	0.00	3.0	Treat as zero by MCPB.py
Y2-M1-Y5-cc	3	0.00	0.00	3.0	Treat as zero by MCPB.py
Y2-M1-Y5-cd	3	0.00	0.00	3.0	Treat as zero by MCPB.py
Y2-M1-Y6-cc	3	0.00	0.00	3.0	Treat as zero by MCPB.py
Y2-M1-Y6-cd	3	0.00	0.00	3.0	Treat as zero by MCPB.py
Y3-M1-Y1-CV	3	0.00	0.00	3.0	Treat as zero by MCPB.py
Y3-M1-Y2-CV	3	0.00	0.00	3.0	Treat as zero by MCPB.py
Y4-M1-Y1-CV	3	0.00	0.00	3.0	Treat as zero by MCPB.py
Y4-M1-Y2-CV	3	0.00	0.00	3.0	Treat as zero by MCPB.py
Y4-cc-ce-cd	1	1.0	180.0	2.0	same as X -ce-ce-X
Y5-M1-Y1-CV	3	0.00	0.00	3.0	Treat as zero by MCPB.py
Y5-M1-Y2-CV	3	0.00	0.00	3.0	Treat as zero by MCPB.py
Y5-cc-ce-cd	1	1.0	180.0	2.0	same as X -ce-ce-X
Y6-M1-Y1-CV	3	0.00	0.00	3.0	Treat as zero by MCPB.py
Y6-M1-Y2-CV	3	0.00	0.00	3.0	Treat as zero by MCPB.py
Y6-cc-ce-cd	1	1.0	180.0	2.0	same as X -ce-ce-X
Y6-cd-ce-cc	1	1.0	180.0	2.0	same as X -ce-ce-X
Y7-M2-Y8-2C	3	0.00	0.00	3.0	Treat as zero by MCPB.py
Y7-M2-Z1-M3	3	0.00	0.00	3.0	Treat as zero by MCPB.py
Y7-M2-Z3-M3	3	0.00	0.00	3.0	Treat as zero by MCPB.py
Y8-M2-Z1-M3	3	0.00	0.00	3.0	Treat as zero by MCPB.py
Y8-M2-Z3-M3	3	0.00	0.00	3.0	Treat as zero by MCPB.py
Z1-M3-Z2-CR	3	0.00	0.00	3.0	Treat as zero by MCPB.py
Z1-M3-Z4-CR	3	0.00	0.00	3.0	Treat as zero by MCPB.py
Z2-M3-Z1-M2	3	0.00	0.00	3.0	Treat as zero by MCPB.py
Z2-M3-Z3-M2	3	0.00	0.00	3.0	Treat as zero by MCPB.py
Z2-M3-Z4-CC	3	0.00	0.00	3.0	Treat as zero by MCPB.py
Z2-M3-Z4-CR	3	0.00	0.00	3.0	Treat as zero by MCPB.py
Z3-M2-Z1-M3	3	0.00	0.00	3.0	Treat as zero by MCPB.py
Z3-M3-Z2-CR	3	0.00	0.00	3.0	Treat as zero by MCPB.py
Z3-M3-Z4-CR	3	0.00	0.00	3.0	Treat as zero by MCPB.py
Z4-M3-Z1-M2	3	0.00	0.00	3.0	Treat as zero by MCPB.py
Z4-M3-Z2-CR	3	0.00	0.00	3.0	Treat as zero by MCPB.py
Z4-M3-Z3-M2	3	0.00	0.00	3.0	Treat as zero by MCPB.py
cc-Y3-M1-Y4	3	0.00	0.00	3.0	Treat as zero by MCPB.py
cc-Y3-M1-Y5	3	0.00	0.00	3.0	Treat as zero by MCPB.py
cc-Y3-M1-Y6	3	0.00	0.00	3.0	Treat as zero by MCPB.py
cc-Y4-M1-Y3	3	0.00	0.00	3.0	Treat as zero by MCPB.py
cc-Y4-M1-Y5	3	0.00	0.00	3.0	Treat as zero by MCPB.py
cc-Y4-M1-Y6	3	0.00	0.00	3.0	Treat as zero by MCPB.py
cc-Y5-M1-Y3	3	0.00	0.00	3.0	Treat as zero by MCPB.py
cc-Y5-M1-Y4	3	0.00	0.00	3.0	Treat as zero by MCPB.py
cc-Y5-M1-Y6	3	0.00	0.00	3.0	Treat as zero by MCPB.py
cc-Y6-M1-Y3	3	0.00	0.00	3.0	Treat as zero by MCPB.py
cc-Y6-M1-Y4	3	0.00	0.00	3.0	Treat as zero by MCPB.py
cc-Y6-M1-Y5	3	0.00	0.00	3.0	Treat as zero by MCPB.py
cc-ce-cd-Y3	1	1.0	180.0	2.0	same as X -ce-ce-X
cc-ce-cd-Y4	1	1.0	180.0	2.0	same as X -ce-ce-X
cc-ce-cd-Y5	1	1.0	180.0	2.0	same as X -ce-ce-X
cd-Y3-M1-Y4	3	0.00	0.00	3.0	Treat as zero by MCPB.py
cd-Y3-M1-Y5	3	0.00	0.00	3.0	Treat as zero by MCPB.py
cd-Y3-M1-Y6	3	0.00	0.00	3.0	Treat as zero by MCPB.py
cd-Y4-M1-Y3	3	0.00	0.00	3.0	Treat as zero by MCPB.py
cd-Y4-M1-Y5	3	0.00	0.00	3.0	Treat as zero by MCPB.py
cd-Y4-M1-Y6	3	0.00	0.00	3.0	Treat as zero by MCPB.py
cd-Y5-M1-Y3	3	0.00	0.00	3.0	Treat as zero by MCPB.py
cd-Y5-M1-Y4	3	0.00	0.00	3.0	Treat as zero by MCPB.py

```
cd-Y5-M1-Y6    3       0.00       0.00   3.0    Treat as zero by MCPB.py
cd-Y6-M1-Y3    3       0.00       0.00   3.0    Treat as zero by MCPB.py
cd-Y6-M1-Y4    3       0.00       0.00   3.0    Treat as zero by MCPB.py
cd-Y6-M1-Y5    3       0.00       0.00   3.0    Treat as zero by MCPB.py
cd-cd-Y3-M1    3       0.00       0.00   3.0    Treat as zero by MCPB.py
cd-cd-Y4-M1    3       0.00       0.00   3.0    Treat as zero by MCPB.py
cd-cd-Y5-M1    3       0.00       0.00   3.0    Treat as zero by MCPB.py
cd-cd-Y6-M1    3       0.00       0.00   3.0    Treat as zero by MCPB.py
cd-ce-cc-Y3    1        1.0      180.0   2.0      same as X -ce-ce-X
ce-cc-Y3-M1    3       0.00       0.00   3.0    Treat as zero by MCPB.py
ce-cc-Y4-M1    3       0.00       0.00   3.0    Treat as zero by MCPB.py
ce-cc-Y5-M1    3       0.00       0.00   3.0    Treat as zero by MCPB.py
ce-cc-Y6-M1    3       0.00       0.00   3.0    Treat as zero by MCPB.py
ce-cd-Y3-M1    3       0.00       0.00   3.0    Treat as zero by MCPB.py
ce-cd-Y4-M1    3       0.00       0.00   3.0    Treat as zero by MCPB.py
ce-cd-Y5-M1    3       0.00       0.00   3.0    Treat as zero by MCPB.py
ce-cd-Y6-M1    3       0.00       0.00   3.0    Treat as zero by MCPB.py
ha-ce-cc-Y3    1        1.0      180.0   2.0      same as X -ce-ce-X
ha-ce-cc-Y4    1        1.0      180.0   2.0      same as X -ce-ce-X
ha-ce-cc-Y5    1        1.0      180.0   2.0      same as X -ce-ce-X
ha-ce-cc-Y6    1        1.0      180.0   2.0      same as X -ce-ce-X
ha-ce-cd-Y3    1        1.0      180.0   2.0      same as X -ce-ce-X
ha-ce-cd-Y4    1        1.0      180.0   2.0      same as X -ce-ce-X
ha-ce-cd-Y5    1        1.0      180.0   2.0      same as X -ce-ce-X
ha-ce-cd-Y6    1        1.0      180.0   2.0      same as X -ce-ce-X

IMPR
CT-CW-CC-Z2         1.1          180.          2.
Y3-cd-cd-ce         1.1          180.0         2.0          Using default value
Y4-cd-cd-ce         1.1          180.0         2.0          Using default value
CT-CW-CC-Z4         1.1          180.          2.
Y6-cc-cc-ce         1.1          180.0         2.0          Using default value
Y6-cd-cd-ce         1.1          180.0         2.0          Using default value
Y4-cc-cc-ce         1.1          180.0         2.0          Using default value
Y5-cd-cd-ce         1.1          180.0         2.0          Using default value
Y5-cc-cc-ce         1.1          180.0         2.0          Using default value
Y3-cc-cc-ce         1.1          180.0         2.0          Using default value

NONB
  M1          1.4090  0.0172100000       IOD set for Fe2+ ion from Li et al. 2013
  M2          1.4090  0.0172100000       IOD set for Fe2+ ion from Li et al. 2013
  M3          1.4090  0.0172100000       IOD set for Fe2+ ion from Li et al. 2013
  Y1          1.8240  0.1700             OPLS
  Y2          1.8240  0.1700             OPLS
  Y7          2.0000  0.2500             W. Cornell CH3SH and CH3SCH3 FEP's
  Z2          1.8240  0.1700             OPLS
  Y8          2.0000  0.2500             W. Cornell CH3SH and CH3SCH3 FEP's
  Z4          1.8240  0.1700             OPLS
  Y3          1.8240  0.1700             OPLS
  Y4          1.8240  0.1700             OPLS
  Y5          1.8240  0.1700             OPLS
  Y6          1.8240  0.1700             OPLS
  Z1          2.0000  0.2500             same as s
  Z3          2.0000  0.2500             same as s
```

Table S5: Derived metal force-field parameters. Comparison of Fe^{2+} bond distance (between Fe^{2+} metal and its interacting residues) among template structure, force field parameters and post-MD (**A**) Heme bL and (**B**) [2FE-2S] cluster located in both PfCytb and ISP chains, respectively.

**A**			**Bond distance (Å): Heme bL**	
	**Template (1PP9)**	**QM derived parameters**	**Force constants (kcal/ mol^{-1} Å^{-2})**	**Post-MD**
**FE_1- NA**	1.970	2.0073	55.9	2.0387±0.00591*
**FE_1- NB**	2.032	2.0251	44.2	2.0479±0.00585*
**FE_1- NC**	1.927	2.0276	46.2	2.0745±0.00581*
**FE_1- ND**	2.007	2.0069	64.2	2.0275±0.00583*
**FE_1-NE2 (His78)**	2.005	2.0205	42.7	2.0546±0.00580*
**FE_1-NE2 (His173)**	2.003	2.0146	43.0	2.0831±0.00581*
**B**			**Bond distance (Å): [2FE-2S]**	
	**Template (1PP9)**	**QM derived parameters**	**Force constants (kcal/mol^{-1} Å^{-2})**	**Post-MD**
**FE_3-SG (Cys299)**	2.279	2.1410	131.0	2.1199±0.00573*
**FE_2-ND1 (His301)**	2.139	2.2053	32.8	2.1507±0.00582*
**FE_3-SG (Cys317)**	2.225	2.1544	118.6	2.1234±0.00583*
**FE_2-ND1 (His320)**	2.147	1.9660	74.0	1.9311±0.00576*

*350 ns MD simulation values provided as an average ± 1 standard deviation

Table S6. Table of residues having high *Betweenness Centrality* (*BC*) values in ATQ-bound protein systems (WT, Y268C, Y268N and Y268S) to assess effect of mutation; + significant positive values while – stands for significant negative values. Active site residues are shown in bold, common residues in all systems (Bold#) and common residues in at least two runs (Bold*). A threshold value of +/- 2 SD of the *BC* values was used for each system.

Betweenness Centrality (BC); **Significant Residues**				
**Mutant systems**		**RUN_1**	**RUN_2**	**RUN_3**
**WT**	+	**PfCytb Chain:** Ser_83, Val_85, Phe_86, Thr_89, Leu_112, Ile_117, Phe_118, Ala_122, **Tyr_126**, Val_127, **Gly_137**, Thr_139, Asn_250, **Val_259*, Pro_260, Glu_261, Tyr_263, Phe_264*, Leu_271**, Lys_277, Gly_280, Leu_291<br>**ISP Chain:** Gly_297, Ile_298, **Cys_299***, **His_301***, Gly_303, **His_320***, Ile_330, Ala_335<br>**ISP Chain:**	**PfCytb Chain:** Ser_83, Val_85, Thr_89, Ala_122, **Phe_123**, Val_127, Thr_139, Thr_160, Thr_165, **Phe_169***, **Trp_262**, Leu_265, Ala_269, Met_270, **Leu_271**, Lys_272, Cys_324<br><br>**ISP Chain:** Lys_252, Ile_296, Gly_297, Ile_298, Leu_302, **His_320***, Ser_322	**PfCytb Chain:** Val_85, Leu_88, Arg_95, Thr_121, **Val_124**, Val_127, **Phe_169***, Ile_237, Ser_241, His_242, Thr_254, **Ile_258, Val_259*, Phe_264***, Leu_265, **Tyr_268**, Val_274, Lys_277, Gly_280, Leu_281, Val_284, Ser_287, Leu_290, Leu_291**ISP Chain:**Gly_297, **Cys_299***, **His_301***, Gly_303, Val_305, Ile_330, Pro_334, Ala_335
**Y268C**	+	**PfCytb Chain:** Ser_83, Val_85, Thr_89, Tyr_99, Leu_112, Phe_118, Ala_122, **Phe_123*, Val_124***, Thr_139, **Ile_141**, Ser_162, **Tyr_263***, **Phe_264#, Leu_271***, Lys_272, Lys_277, Val_284<br><br>**ISP Chain:** Leu_226, Ile_298, **His_301#**, Leu_302, Gly_303, **His_320#**, Ser_322, Ile_330	**PfCytb Chain:** Ala_82, Val_85, Arg_95, Phe_118, Thr_121, Ala_122, **Phe_123*, Val_124***, Val_127, Ala_138, Thr_160, Thr_165, Arg_168, **Phe_169**, **Leu_172**, Leu_176, **Phe_264#, Leu_271***, Lys_272, Lys_277, Leu_281, Ile_320<br><br>**ISP Chain:** Lys_252, **His_301#**, Leu_302, Gly_303, **His_320#**, Ser_322, Ile_330	**PfCytb Chain:** Ser_83, Thr_89, Leu_112, Phe_118, Thr_121, Ala_122, Tyr_126, Val_127, **Met_133, Gly_137**, Thr_139, Pro_255, **Pro_260, Tyr_263*, Phe_264#, Cys_268,** Lys_272, Lys_277, Leu_288<br><br>**ISP Chain:** Gly_297, Ile_298, **His_301#**, Gly_303, Val_305, **His_320#**, Ser_322, Ile_330
**Y268N**	+	**PfCytb Chain:** Ser_83, Val_85, Phe_86, Thr_89, Leu_112, Val_127, **Met_133***, Tyr_252, Thr_254, **Ile_258**, **Val_259, Pro_260, Tyr_263*, Phe_264***, **Asn_268***, Lys_272, Lys_277<br>**ISP Chain:** Gly_297, **His_301***, Leu_302, Val_305, **His_320#**, Ser_322, Ile_330	**PfCytb Chain:** Val_85, Phe_86, Thr_89, Leu_112, Phe_118, **Ile_119**, Ala_122, Ser_134, Asn_143, Val_249, **Tyr_263*, Phe_264*, Asn_268***, Ala_269, Lys_272, Lys_277<br><br>**ISP Chain:** Ile_298, **His_301***, Leu_302, Gly_303, **His_320#**, Ser_322, Ile_330	**PfCytb Chain:** Leu_112, Ala_122, **Phe_123, Val_124**, Tyr_126, Val_127, **Met_133***, Ser_134, **Trp_136**, Thr_139, Ile_237, Ser_241, His_242, Phe_264, Met_270, **Leu_271**, Lys_272, Leu_281<br><br>**ISP Chain:** Gly_297, Ile_298, Gly_303, **His_320#**, Ser_322, Ile_330, Pro_334, Ala_335
**Y268S**	+	**PfCytb Chain:** Ser_83, Val_85, Phe_86, Thr_89, Ile_93, Leu_97, Leu_112, Ala_122, **Phe_123*, Val_140*, Ile_141, Ile_258, Trp_262, Phe_264#**, Leu_265, **Ser_268***, Lys_272, Leu_288<br>**ISP Chain:** Gly_297, Ile_298, **His_301***, Gly_303, Val_305, **His_320#**, Ser_322	**PfCytb Chain:** Val_85, Phe_86, Thr_89, Arg_95, Phe_118, Thr_121, **Val_124**, Val_127, **Gly_137*, Val_140***, Thr_160, Val_161, Thr_165, **Phe_169, Tyr_263*, Phe_264#, Ser_268*, Leu_271**, Lys_272, Lys_277, Leu_281, Gln_289, Leu_290, Phe_292<br>**ISP Chain:** Lys_252, **His_301***, Leu_302, **His_320#**, Ser_322, Ile_330	**PfCytb Chain:** Ala_82, Val_85, Phe_86, Thr_89, Leu_112, Ala_122, **Phe_123*, Trp_136, Gly_137*,** Thr_142, Asn_143, Thr_160, Thr_165, **Tyr_263*, Phe_264#**, Lys_272, Leu_290, Leu_294 **ISP Chain:** Gly_297, Ile_298, Leu_302, Val_305, **His_320#**, Pro_336

Table S7. Table of residues having significant changes in *Betweenness Centrality* (Δ*BC*) in three Holo mutant systems to assess effect of mutation; + significant positive values while – stands for significant negative values. Active site residues are shown in bold, common residues in all systems (Bold#) and common residues in at least two runs (Bold*). A threshold value of +/- 2 SD of the Δ*BC* values was used for each system.

Betweenness Centrality (ΔBC); **(WT– Mutant); Significant Residues**				
**Mutant systems**		**RUN_1**	**RUN_2**	**RUN_3**
**Y268C**	+	**PfCytb chain:** Ser_83, Ile_93, Asn_143, **Trp_262#,** Leu_265, **Cys_268,** Lys_272, Val_274, Ile_283, Leu_288<br>**ISP chain:** Trp_221, Ile_222, Ile_298, Leu_302, His_320, Ser_322, Ile_330	**PfCytb chain:** Trp_26, Tyr_101, Met_102, Phe_115, **Ile_119*, Val_124**, Thr_160, Gln_257, **Ile_258, Val_259**, **Trp_262**#, Leu_265, Leu_291<br><br>**ISP chain:** Val_227	**PfCytb chain:** Leu_25, Leu_94, **Ile_119***, Ala_122, **Phe_123, Trp_136, Ile_141**, Ile_237, Ser_241, **Trp_262**#, Leu_265, Thr_301, Trp_331, Val_305
	-	**PfCytb chain:** Phe_86, Met_102, Phe_118, Ala_122, **Phe_123***, Thr_160, Val_161, His_187, Gln_232, **Glu_261**	**PfCytb chain:** Leu_94, **Val_120, Phe_123***, Ala_138, Thr_139, **Val_140, Ile_141**, Ser_241, Val_249, Tyr_263, Gln_289, Leu_290, Cys_324, Leu_357<br><br>**ISP chain:** Ser_223, Cys_304, Pro_334	**PfCytb chain:** Tyr_99, Leu_112, **Ile_155**, Thr_160, Thr_165, Arg_168, Leu_240, Asp_244, Tyr_252, Gln_257, **Phe_264**, Lys_252, Leu_302
**Y268N**	+	**PfCytb chain:** Ser_83, Ala_122, **Phe_123*, Val_140,* Ile_141***, Asn_143, **Leu_144**, **Trp_262#,** Leu_265, **Asn_268**, Lys_272, Ile_309<br>**ISP chain:** Trp_221, Ile_222, Leu_302	**PfCytb chain:** Trp_26, Phe_118, Ala_122, Thr_197, Ile_247, Thr_251, Gln_257, **Ile_258, Val_259**, **Trp_262**#, Lys_277, Leu_281, Leu_291<br><br>**ISP chain:** Ile_222, Ile_298, Cys_304, Pro_318	**PfCytb chain:** Phe_118, Ala_122, **Phe_123*, Trp_136, Val_140,* Ile_141***, Thr_160, Ser_162, Ile_237, **Trp_262#,** Leu_265, Leu_294, Val_305
	-	**PfCytb chain:** Phe_86, **Val_124**, Thr_160, Phe_169, Ile_237, Ser_241, His_242, Asn_250, Tyr_263, **Phe_264**#, Lys_277, Leu_357	**PfCytb chain:** Ser_241, His_242, Val_253, Thr_254, **Pro_260***, Tyr_263, **Phe_264**#, **Asn_268**, Ile_342, Pro_334, Pro_336	**PfCytb chain:** Phe_30, His_92, Leu_112, Ile_155, Leu_172, Leu_176, Leu_215, Tyr_252, **Pro_260*, Phe_264**#, Val_274, Leu_288, Gly_303, Pro_334, Pro_336
**Y268S**	+	**PfCytb chain:** Thr_80, Ser_83, Asn_143, Tyr_252, **Trp_262**#, Leu_265, **Ser_268**, Lys_272, Leu_281, Leu_288, Leu_290, Leu_291<br>**ISP chain:** Ile_222, Leu_302, His_320*, Ile_330	**PfCytb chain:** Tyr_101, Met_102, Phe_115, **Ile_119***, Thr_197, Thr_251, Gln_257, **Ile_258, Val_259***, **Glu_261**, **Trp_262**#, **Phe_264**, Lys_277, Leu_291, Leu_294, Cys_304	**PfCytb chain:** Leu_25, Ala_82, **Ile_119***, Ala_122, **Phe_123**, Gly_131, **Trp_136**, Ile_237, Asn_250, **Val_259***, **Trp_262**#, Leu_265<br><br>**ISP chain:** Ile_222, His_320*
	-	**PfCytb chain:** Ile_35, Val_85, Phe_86, Thr_89, Met_102, Trp_108, **Val_120, Leu_145** Ser_146, Tyr_159, Thr_160, **Pro_260, Glu_261, Leu_271**, Cys_304, Pro_318	**PfCytb chain:** Tyr_16, Val_127, **Trp_136**, Asn_143, Ser_146, Ile_188, Ser_241, His_242, **Tyr_263***, Cys_324, Cys_319*	**PfCytb chain:** Ser_83, Thr_121, Ala_138, Thr_139, **Ile_155**, **Tyr_263*, Phe_264, Phe_267**, **Ser_268,** Val_274<br>**ISP chain:** Leu_226, Cys_304, Cys_319*, Gly_321, Pro_334

Table S8. Table of residues having significant changes in *Betweenness Centrality* (Δ*BC*) in three ATQ-bound mutant systems to assess effect of mutation; + significant positive values while – stands for significant negative values. Active site residues are shown in bold, common residues in all systems (Bold#) and common residues in at least two runs (Bold*). A threshold value of +/- 2 SD of the Δ*BC* values was used for each system.

Betweenness Centrality (ΔBC); **(WT– Mutant); Significant Residues**				
**Mutant systems**		**RUN_1**	**RUN_2**	**RUN_3**
**Y268C**	+	**PfCytb Chain:** Phe_86, Arg_95, Val_127, **Gly_137**, Thr_139, Pro_164, Arg_168, **His_187**, **Val_259*, Pro_260, Glu_261, Phe_26**4, Lys_277, Gly_280, Leu_291<br>**ISP Chain:** Ile_298, **Cys_299**, **His_301**, Gly_303, **Cys_319,** Ala_335	**PfCytb Chain:** Leu_25, Ser_83, Phe_86, Tyr_90, Thr_139, **Ile_141**, Cys_156, Pro_164, Leu_191, His_242, Asp_244, **Trp_262**, Leu_265, Ala_269, Met_270, Lys_272, Cys_324, Tyr_363<br>**ISP Chain:** Trp_221, Ile_298	**PfCytb Chain: Ile_155**, Thr_160, **Phe_169**, Ile_237, Ser_241, His_242, Thr_254, **Ile_258**, **Val_259***, Leu_265, Thr_273, Val_274, Gly_280, Leu_281, Ser_287, Pro_334, Ala_335
	-	**PfCytb Chain:** Tyr_99, **Met_116, Phe_123, Trp_136, Ile_141, Leu_144,** Ser_162, Ser_196, Ser_241, Ser_287<br>**ISP Chain:** Leu_226, Lys_252, Leu_302, Ser_322	**PfCytb Chain:** Asn_27, Leu_31, Arg_95, Met_102, Phe_118, Ala_122, Thr_160, **Leu_172**, Ala_246, **Phe_264***, Lys_277, Leu_281, **Leu_285**, Gln_289, Ile_320<br>**ISP Chain:** Phe_219, Gly_251, Lys_252, **His_301**	**PfCytb Chain:** Ser_83, Thr_89, Ala_122, **Phe_264***, Leu_271, Lys_272, Tyr_327, Pro_337<br>**ISP Chain: His_320**, Gly_321, Ser_322
**Y268N**	+	**PfCytb Chain:** Tyr_16, Ala_122, **Gly_137**, Thr_139, Ile_247, Leu_271, Pro_337, Ile_298, Gly_303, Ala_335	**PfCytb Chain:** Ser_83, Thr_139, Thr_160, Ser_162, Pro_164, Thr_165, **Trp_262**, Leu_265, Met_270, Leu_271, Cys_324, Gly_333<br>**ISP Chain:** Trp_221	**PfCytb Chain:** Tyr_28, Val_85, Leu_88, Thr_160, **Phe_169**, Asn_245, **Ile_258, Val_259**, Val_274, Pro_275, Ser_276, Lys_277, Gly_280, Ser_287, Leu_294, Ile_309<br>**ISP Chain: His_301**, Val_305
	-	**PfCytb Chain:** Leu_94, **Met_133**, Ser_241, His_242, Tyr_252, Thr_254, **Ile_258**, Lys_272<br>**ISP Chain:** Val_305, **His_320***, Ser_322	**PfCytb Chain:** Phe_86, **His_92**, Phe_118, Gly_131, **His_192***, Asn_245, Val_249, Tyr_252, **Tyr_263, Phe_264, Tyr_268**, Pro_278<br>**ISP Chain: His_301**, Gly_303	**PfCytb Chain:** Ala_122, **Trp_136**, Ser_147, **Leu_172**, **His_187**, **His_192***, **Leu_271**, Lys_272<br>**ISP Chain:** Cys_324, **His_320***, Ser_322, Pro_334, Pro_336
**Y268S**	+	**PfCytb Chain:** Arg_95, **Gly_137**, Thr_139, Ile_247, **Pro_260, Phe_26**4, Lys_277, Leu_291<br>**ISP Chain:** Ile_298, **His_301***, Gly_303, **Cys_319**, Ala_335	**PfCytb Chain:** Leu_25, Ala_122, **Phe_123**, Thr_139, Pro_164, Ile_188, **Trp_262**, Leu_265, Ala_269, Lys_272, Leu_288, Cys_324, Ala_328<br>**ISP Chain:** Ile_298, Leu_302	**PfCytb Chain: Val_124**, Val_127, **Phe_169**, Ile_237, Ser_241, His_242, Thr_254, Pro_255, **Ile_258, Val_259**, Leu_265, Val_274, Lys_277, Gly_280, Leu_291 **ISP Chain: His_301***
	-	**PfCytb Chain:** Thr_89, Ile_93, Leu_97, **Phe_123*, Met_133, Val_140, Ile_141**, Thr_160, Ser_241, **Trp_262**, Leu_265, Cys_304, Ser_322	**PfCytb Chain:** Phe_86, Arg_95, Leu_97, Met_102, **Gly_137, Val_14**0, **Leu_144**, Val_161, Ser_241, **Tyr_263**, **Phe_264**, Lys_277, Leu_281, Leu_285, Gln_289, Leu_290, Phe_292 **ISP Chain: His_301**	**PfCytb Chain:** Phe_86, Thr_89, Ala_122, **Phe_123*, Trp_136**, **Thr_142**, Asn_143, Gly_195, **Tyr_263***, Lys_272<br>**ISP Chain:** Tyr_327, Pro_337, **His_320**, Pro_336

Table S9. Residues having significant changes in average shortest path (Δ*L*) in three Holo mutant systems to assess effect of mutation ; + significant positive values while – stands for significant negative values. Interfacing residues in Bold, common residues in all systems (Bold#) and common residues in at least two runs (Bold*). A threshold value of +/- 2 SD of the Δ*L* values was used for each system.

<table>
<tr><th colspan="5">Average Shortest Path (ΔL); (WT– Mutant); Significant Residues</th></tr>
<tr><th>Mutant systems</th><th></th><th>RUN_1:</th><th>RUN_2</th><th>RUN_3</th></tr>
<tr><td rowspan="2">Y268C</td><td>+</td><td>PfCytb chain: Arg_298
ISP chain: Val_227, Ala_228*, Gly_229, Gly_251, Lys_272, Leu_273, Thr_276</td><td>PfCytb chain: Pro_17, Pro_19, Leu_20, Asn_23, Leu_25, Leu_94, Asn_98, Ile_237, Ser_241, His_242, Pro_243, Asn_245, Lys_307, Leu_357, Tyr_368
ISP chain: Lys_215</td><td>PfCytb chain: Asn_15, Tyr_16, Ala_151, Gly_201, Arg_298, Phe_306, Lys_307, Met_308, Ile_309, Phe_310,
ISP chain: Leu_226, Ala_228*, Gly_229, Glu_262, Asp_263, Arg_266, Lys_272, Leu_273, Thr_276</td></tr>
<tr><td>-</td><td>PfCytb chain: Gly_221, Phe_222, Asn_223, Val_225, Ile_226, Phe_229, Leu_230, Ser_233, Leu_234, Gly_311, Ala_312
ISP chain: Cys_214, Ser_216, Val_217, His_218, Trp_221</td><td>PfCytb chain: Thr_302, Gln_373, Ala_374, Asn_375
ISP chain: Ile_222*, Lys_224, Val_227, Ala_228, Gly_251</td><td>PfCytb chain: Leu_25, Ala_328
ISP chain: Phe_219, Phe_220, Trp_221, Ile_222*, Lys_224</td></tr>
<tr><td rowspan="2">Y268N</td><td>+</td><td>PfCytb chain: Ala_205, Ser_241, His_242, Asn_375
ISP chain: Leu_273, Thr_276</td><td>PfCytb chain: Asn_224, Phe_235, Gly_236, Ile_237, Ile_238, Ser_241, His_242, Pro_243, Asp_244, Asn_245, Thr_254,
ISP chain: Lys_215, Ser_216, Val_217, Thr_276, Glu_349</td><td>PfCytb chain: Ala_151, Leu_200, Leu_215, Ile_304
ISP chain: Cys_214, Ala_228, Glu_262, Asp_263, Gln_265, Arg_266, Ala_267, Lys_268, Glu_269</td></tr>
<tr><td>-</td><td>PfCytb chain: Asn_15, Ser_147, Lys_207, Met_308, Ile_309, Phe_310, Gly_311, Asp_314, His_367, Asp_369, Tyr_370,
ISP chain: Cys_214, Lys_215, Ser_216, Val_217, His_218, Phe_220, Trp_221#, Gln_265, Arg_266, Glu_269</td><td>PfCytb chain: Ser_147, Gly_195, Ser_196, Ala_312, Arg_313, Gln_373, Ala_374, Asn_375
ISP chain: Trp_221#, Ile_222*, Ser_223, Lys_224, Glu_262, Asp_263</td><td>PfCytb chain: Lys_207, Asn_224, Val_225, Leu_228, Ile_231, Gln_232, Ile_237, Pro_239, Leu_240, Lys_307, Met_308, Ile_309
ISP chain: Val_217, Trp_221#, Ile_222*, Leu_234</td></tr>
<tr><td rowspan="2">Y268S</td><td>+</td><td>PfCytb chain: Phe_235, Gly_236, Pro_239, Asp_270, Gln_275</td><td>PfCytb chain: Asn_15, Tyr_16, Pro_17, Pro_19, Leu_206, Ile_208, Pro_209, Phe_210, Asn_213, Gly_236, Ile_237, Ile_238, Ser_241, His_242, Pro_243, Asp_244, Asn_245
ISP chain: Lys_215, Ser_223, Glu_349</td><td>PfCytb chain: Ser_147, Val_150, Ala_151, Phe_306, Lys_307, Met_308, Ile_309, Phe_310
ISP chain: Cys_214, Lys_215, Ser_216, Leu_226, Val_227</td></tr>
<tr><td>-</td><td>PfCytb chain: Asn_15, Tyr_16, Pro_199, Ile_304, Ile_309, Phe_310, Gly_311, Asp_314, Ser_316
ISP chain: Cys_214, Lys_215, His_218</td><td>PfCytb chain: Ile_22, Gly_195, Ser_196, Thr_197, Asn_198, Pro_199, Leu_200, Leu_215, Gln_257, Gln_297, Arg_298, Ser_299, Met_308, Ile_309, Phe_310, Ser_371, Gln_373, Asn_375
ISP chain: Val_227</td><td>PfCytb chain: Leu_25, Asp_203, Val_248, Asn_250, Thr_251, Tyr_252, Ile_340
ISP chain: Ile_222</td></tr>
</table>

Table S10. Residues having significant changes in average shortest path (Δ*L*) in three ATQ-bound mutant systems to assess effect of mutation ; + significant positive values while – stands for significant negative values. Interfacing residues in Bold, common residues in all systems (Bold#) and common residues in at least two runs (Bold*). A threshold value of +/- 2 SD of the Δ*L* values was used for each system.

**Average Shortest Path (Δ*L*)**; (WT– Mutant); Significant Residues				
**Mutant systems**		**RUN_1:**	**RUN_2**	**RUN_3**
**Y268C**	+	**PfCytb Chain:** Gly_158, Tyr_159, Thr_160, Ser_216, Asp_218, Cys_334, Ala_374, Asn_375<br>**ISP Chain: Cys_214**#, Phe_220, Glu_262, Asp_263, Arg_266, Ala_267, Lys_268, Asp_270	**PfCytb Chain:** Thr_197, Tyr_202, Arg_364, Arg_365, Tyr_368, Asp_369, Tyr_370, Ser_371, Ser_372, Gln_373, Ala_374, Asn_375<br>**ISP Chain: Cys_214**#, Val_217, His_218, Phe_219	**PfCytb Chain:** Asn_15, Tyr_16, Pro_17, Pro_209, Phe_210, Tyr_211, Pro_212, Leu_215, Pro_337, Gln_338, Ile_340,<br>**ISP Chain: Cys_214#,** Lys_215, Ser_216, Phe_219, Gln_275, Gly_309, Gly_310, Asn_311
	-	**PfCytb Chain:** Asn_15, Tyr_16, Pro_17, Ser_70, Pro_199, Leu_200, Tyr_202, Asp_203, Thr_204, Ala_205, Pro_209, Pro_212, **Pro_239#, Leu_240**#, Val_259, Gln_297, Arg_298, Leu_300<br>**ISP Chain:** Leu_273	**PfCytb Chain:** Asn_15, Leu_25, Thr_204, **Ile_238*, Pro_239**#, **Leu_240**#, Ser_241, His_242, Pro_243, Asp_244, Asn_245<br>**ISP Chain:** Ser_223, Lys_224, Asp_225, Val_227, Lys_272	**PfCytb Chain:** Pro_149, Val_150, Ile_155, Ser_196, Pro_199, Gly_236, Ile_237, **Ile_238*, Pro_239#, Leu_240#,** Ser_241, His_242, Pro_243, Met_308, Ile_309, Phe_310,<br>**ISP Chain:** Trp_221, Ile_222
**Y268N**	+	**PfCytb Chain:** Pro_199, Ser_216, Asp_218, Phe_235, Gly_236, Ile_237, Ile_238, Ser_241, His_242, Pro_243, Asn_245, Cys_334<br>**ISP Chain: Cys_214*, Lys_215***, His_218, **Phe_219***	**PfCytb Chain:** Ile_303, Leu_360, Arg_364, Arg_365, Thr_366, Tyr_368, Asp_369, Tyr_370, Ser_372, Gln_373, Ala_374, Asn_375<br>**ISP Chain:** Ser_216, Val_217, **Phe_219***, Phe_220, Glu_262, Asp_263, Gln_265, Arg_266, Glu_269	**PfCytb Chain:** Ser_147, Ile_304, Pro_337, Gln_338<br>**ISP Chain: Cys_214*, Lys_215***, Ser_216, Gly_309, Gly_310, Asn_311, Pro_336
	-	**PfCytb Chain:** Asn_15, Tyr_16, Pro_105, Ser_147, Pro_149, Val_161, Pro_209, Pro_212, Ser_299, Pro_337, Tyr_368, Asp_369<br>**ISP Chain: Ser_223***, Asp_225, **Leu_226*, Val_227***	**PfCytb Chain:** Asn_21, Asn_23, Pro_199<br>**ISP Chain: Ser_223*** , Lys_224, **Leu_226* , Val_227*** , Lys_272, Leu_273, Thr_276, Asn_311	**PfCytb Chain:** Tyr_28, Gly_229, Gly_230, Gly_251, Ala_267, Lys_268, Glu_269, Asp_270, Asp_271, Lys_272, Leu_273, Ile_274
**Y268S**	+	**PfCytb Chain:** Asn_21, Ser_216, Asp_218, Cys_334<br>**ISP Chain: Cys_214, Lys_215, Ser_216, His_218, Phe_219***, Arg_266, Ala_267, Lys_268	**PfCytb Chain:** Ser_147, Asn_213, Leu_214, Ile_304, Arg_364, Arg_365, Tyr_368, Asp_369, Tyr_370, Ser_371, Ser_372, Gln_373, Ala_374, Asn_375,<br>**ISP Chain:** Glu_262, Asp_263, Gln_265, Arg_266, Ala_267, Lys_268	**PfCytb Chain:** Thr_142, Ile_148, Phe_210, Leu_215, Arg_298, Pro_337, Gln_338<br>**ISP Chain: Phe_219***, Gln_275, Thr_276, Met_277, Arg_278, Gly_310, Asn_311, Pro_336
	-	**PfCytb Chain:** Asp_203, Thr_204, Ala_205, Pro_209, Pro_239, Glu_296, Gln_297, Ser_299, Leu_300, Thr_301, Thr_302, Ile_303, Val_361, Arg_365, Tyr_370, Ser_371, Ser_372, Asn_375<br>**ISP Chain: Leu_226***	**PfCytb Chain:** Asn_15, Asn_23, Leu_25, Ala_205, Ala_328<br>**ISP Chain:** Lys_215, Ser_223, **Asp_270***, Asp_271, Lys_272, Leu_273, Thr_276	**PfCytb Chain:** Asp_203, Leu_228, Phe_235, Gly_236, Ile_237, Ile_238, Ser_241, His_242, Pro_243, Asp_244, Asn_245, Gln_257, Ala_312, Arg_313, Cys_334, Asn_375<br>**ISP Chain:** Asp_225, **Leu_226**, **Asp_270**

Table S11. Molecular composition of the simulation systems

System	Bilayer	Lipids	Water	Counter Ions (added after tleap)	
				Na+	Cl-
Wild-type	POPC:POPE	990	29497	3	4
Y268C	POPC:POPE	984	29574	3	4
Y268N	POPC:POPE	987	29710	2	3
Y268S	POPC:POPE	981	29504	2	3

Table S12. Computational research tools used in the study and brief descriptions of their functionalities.

Program	Functionality/Description
AmberTools [1]	A suite of MD tools and associated programs that allow users to carry out molecular dynamics simulations on biomolecules.
CPPTRAJ [2]	A program designed for processing and analysing MD simulation trajectories including RMSD, Rg, RMSF as well as hydrogen bond analysis. It can also perform clustering analysis between trajectories or between frames of the same trajectory. It is a part of AmberTools.
Discovery Studio Visualizer [3]	3D molecular graphics visualization tool used to view and analyze macromolecular structures.
Gaussian09 [4]	Computational chemistry software package. It employs laws of quantum mechanics to perform a multitude of calculations to predict energies, molecular structures, and spectroscopic data.
GaussView [5]	GaussView is a graphical interface for Gaussian, it mainly assists with pre-and post-processing of jobs from Gaussian.
GROMACS [6]	Tool for molecular dynamics simulations of macromolecular structures. The software package also contains functionality for trajectory analysis .
H++ server [7]	Open source web-server used for the protonation of a protein structure. It first computes the pK values of ionizable groups in the protein, then adds missing protons according to the pH of the environment. It outputs parameters and coordinate files for simulations, currently in AMBER format.
I-TASSER [8–10]	Template-based prediction tool for protein structures and function. Structure prediction proceeds by threading with the use of one main template along with other templates. The Local Meta-Threading Server,(LOMETS) is utilized for threading and the BioLIP protein function database is used to predict the function of the model.
Maestro [11]	Molecular modeling software. It is also a molecular graphics visualization tool used to view and analyze macromolecular structures.
MD-TASK [12]	A suite of Python scripts that It employs graph theory techniques, perturbation response scanning, and dynamic cross-correlation for analyzing MD trajectories.
MCPB.py [13]	Python-based metal center parameter builder for the modeling of metal centers and simulation of metal complexes. It involves a semi-automated workflow of parameterization schemes to drive both force constants of metals and charged parameters.
Packmol-memgen [14]	Generalized workflow for automated building of membrane-protein-lipid-bilayer systems.
PROCHECK [15]	A suite of programs used to assess the stereochemical quality of proteins. It produces a number of Pos-script plots showing the overall analysis of the protein and residues by residue geometry, it further highlights regions that may need investigation.
PyMOL [16]	3D molecular graphics visualization tools used to view and analyze macromolecular structures.
RAMPAGE [17]	A Web server used for the validation of 3D structure modeled.
VMD [18]	Molecular modeling and visualization computer program.

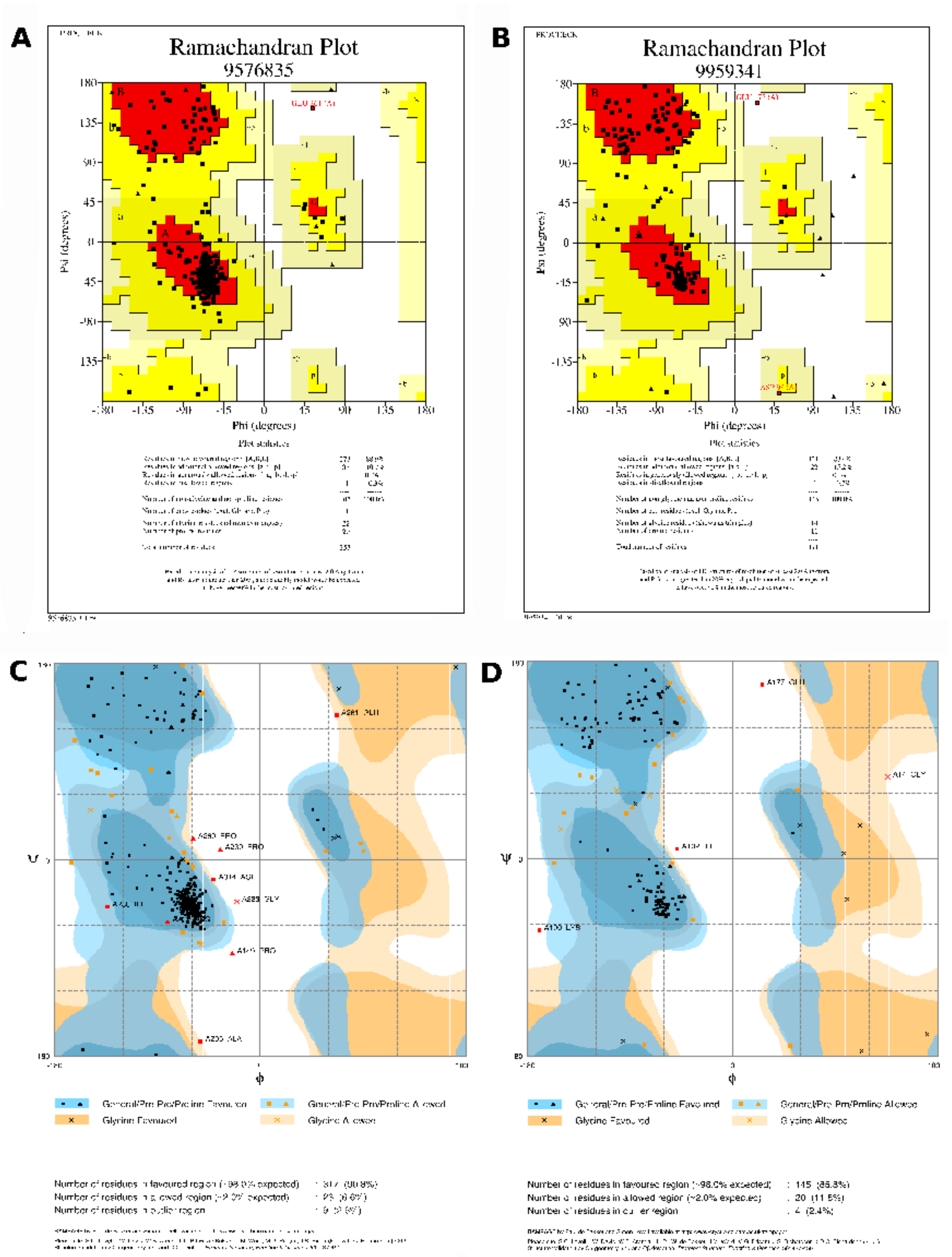

Figure S1. Predicted protein model validation in form of Ramachandran plots. (**A**) and (**B**) representing PfCytb and ISP respectively using PROCHECK as well as (**C**) and (**D**) representing PfCytb and ISP respectively using RAMPAGE. Most of sthe residues are observed to be in the most favored and allowed regions. Overall, the model quality was good.

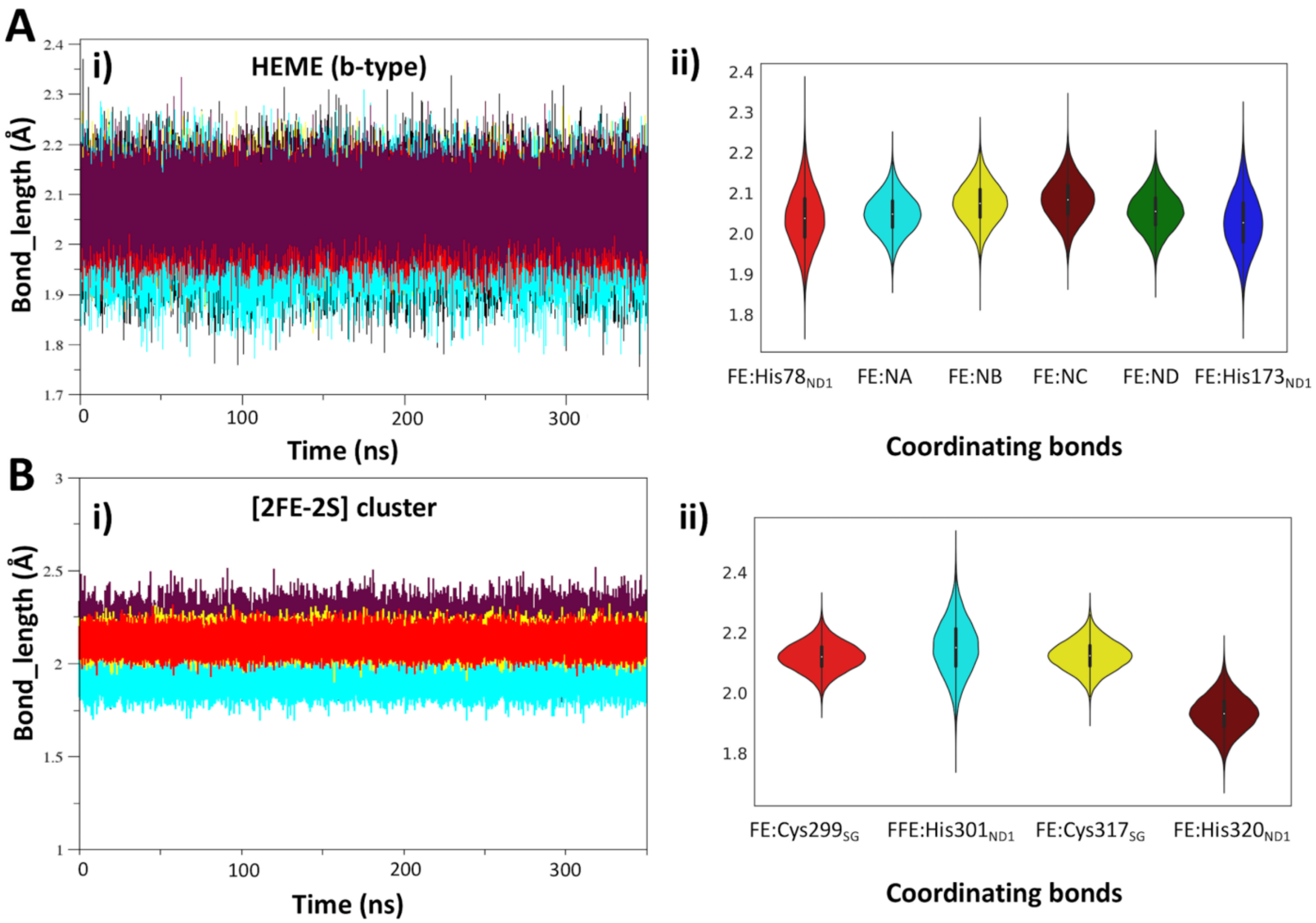

Figure S2. Calculated bond distances monitored during 350 ns MD simulations. (**A**) and (**B**) represents i) Heme and ii) [2FE-2S] cofactors, respectively. Metal ion FE stands for FE_1 for the heme and two iron metals (FE_2 and FE_3) for the 2FE-2S] cluster. The various bond distances are also represented as violin plots for distinction of some of the overlapping bonds present in the line graph. Fe^{2+} bonds to His78, His173, NA, NB, NC, and ND are shown in red, blue, cyan, yellow, maroon and green respectively for Heme cofactor while Fe^{2+} bonds to His301, His320, Cys299 and Cys317 are shown in red, cyan, maroon and yellow colors respectively for 2FE-2S] cluster.

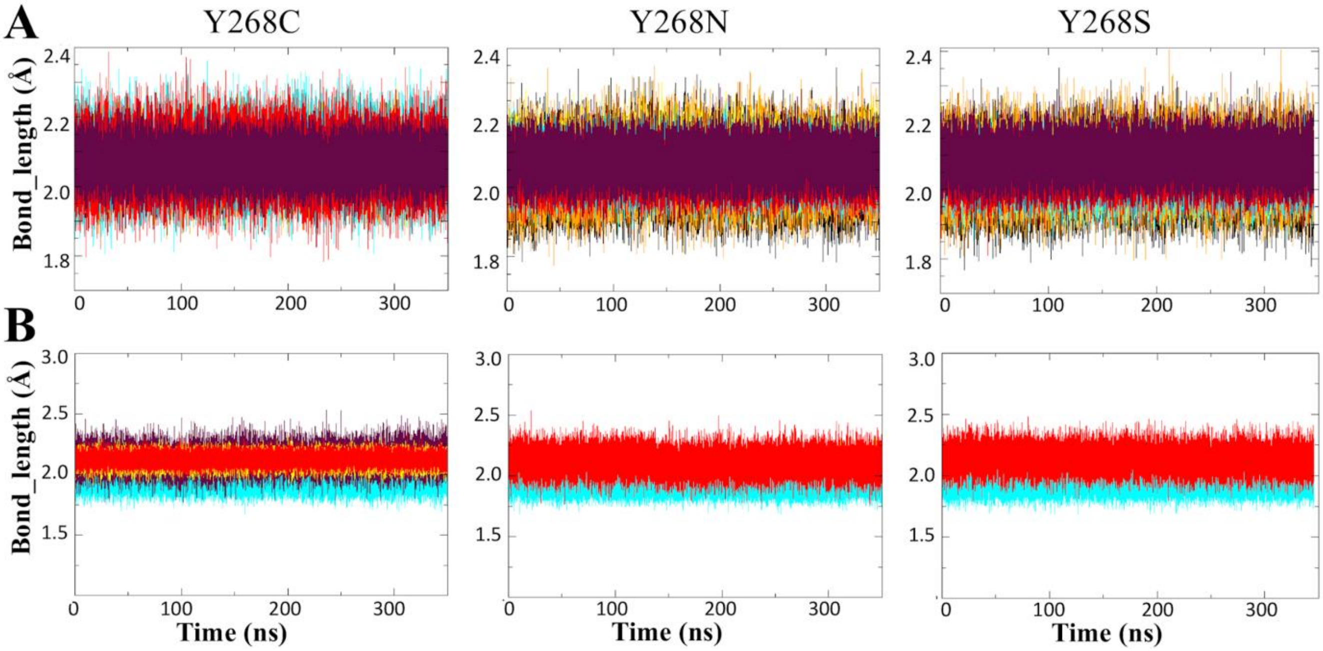

Figure S3. Calculated bond distances monitored during 350 ns MD simulations in the mutant systems (Y268C, Y268N and Y268S). Graphs shown in (**A**) and (**B**) represents Heme and [2FE-2S] cluster cofactors, respectively. Fe^{2+} bonds to His78, His173, NA, NB, NC, and ND are shown in red, blue, cyan, yellow, maroon and green respectively for Heme cofactor while Fe^{2+} bonds to His301, His320, Cys299 and Cys317 are shown in red, cyan, maroon and yellow colors respectively for 2FE-2S] cluster. Metal parameters were successfully transferred to the systems prior to MD simulation. The bond distances are comparable to the WT system shown in Figure S2.

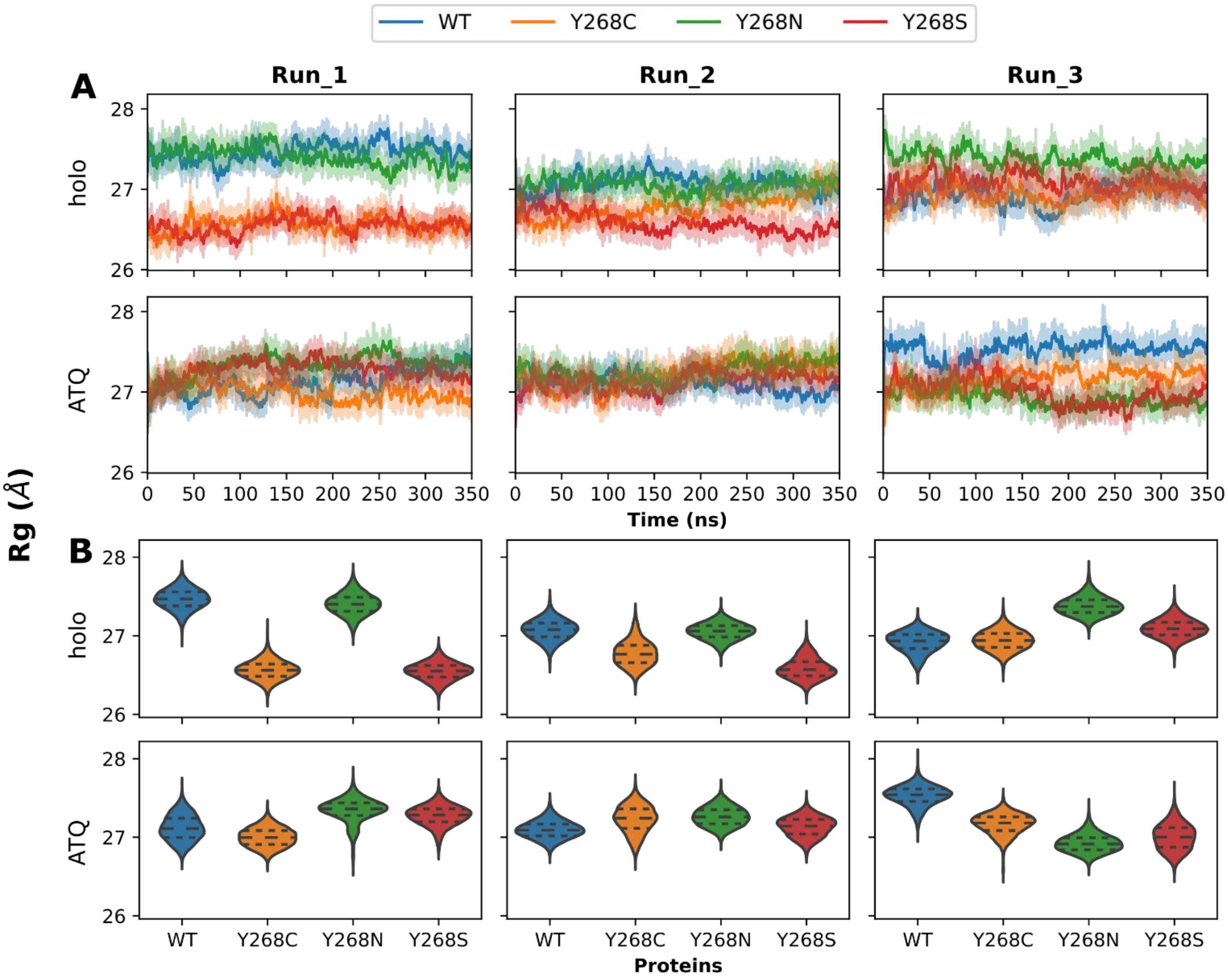

Figure S4. Rg comparison of WT and mutant both in the presence and absence of ATQ during the 350 ns MD simulation. A Line graph representation. **B** Violin plot presentation

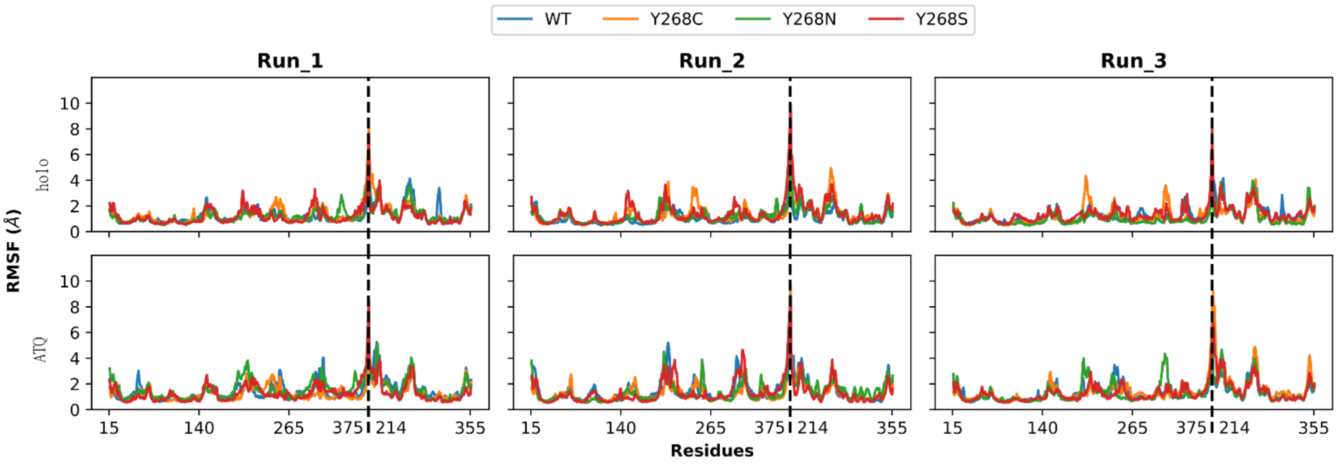

Figure S5. Line graph comparison of RMSF as observed in the WT and mutant PfCytb-ISP protein systems during 350 ns MD simulation in the presence and absence of ATQ. A total of three replicate runs were calculated and displayed. High values show regions of high flexibility.

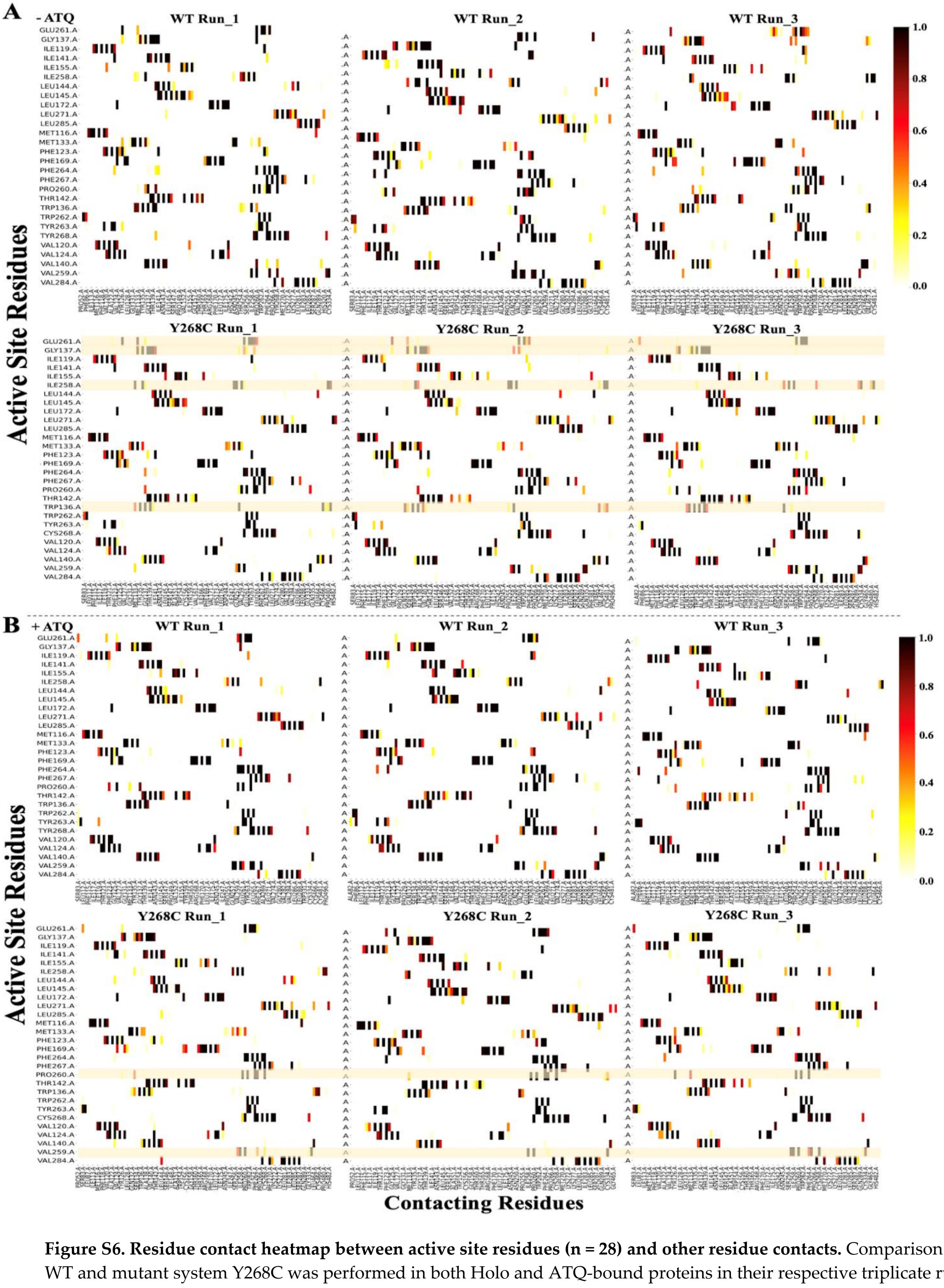

Figure S6. Residue contact heatmap between active site residues (n = 28) and other residue contacts. Comparison of WT and mutant system Y268C was performed in both Holo and ATQ-bound proteins in their respective triplicate runs in the last 100 ns of MD simulation time. Regions observed to reduce or lose residue contact are shaded in light golden color.

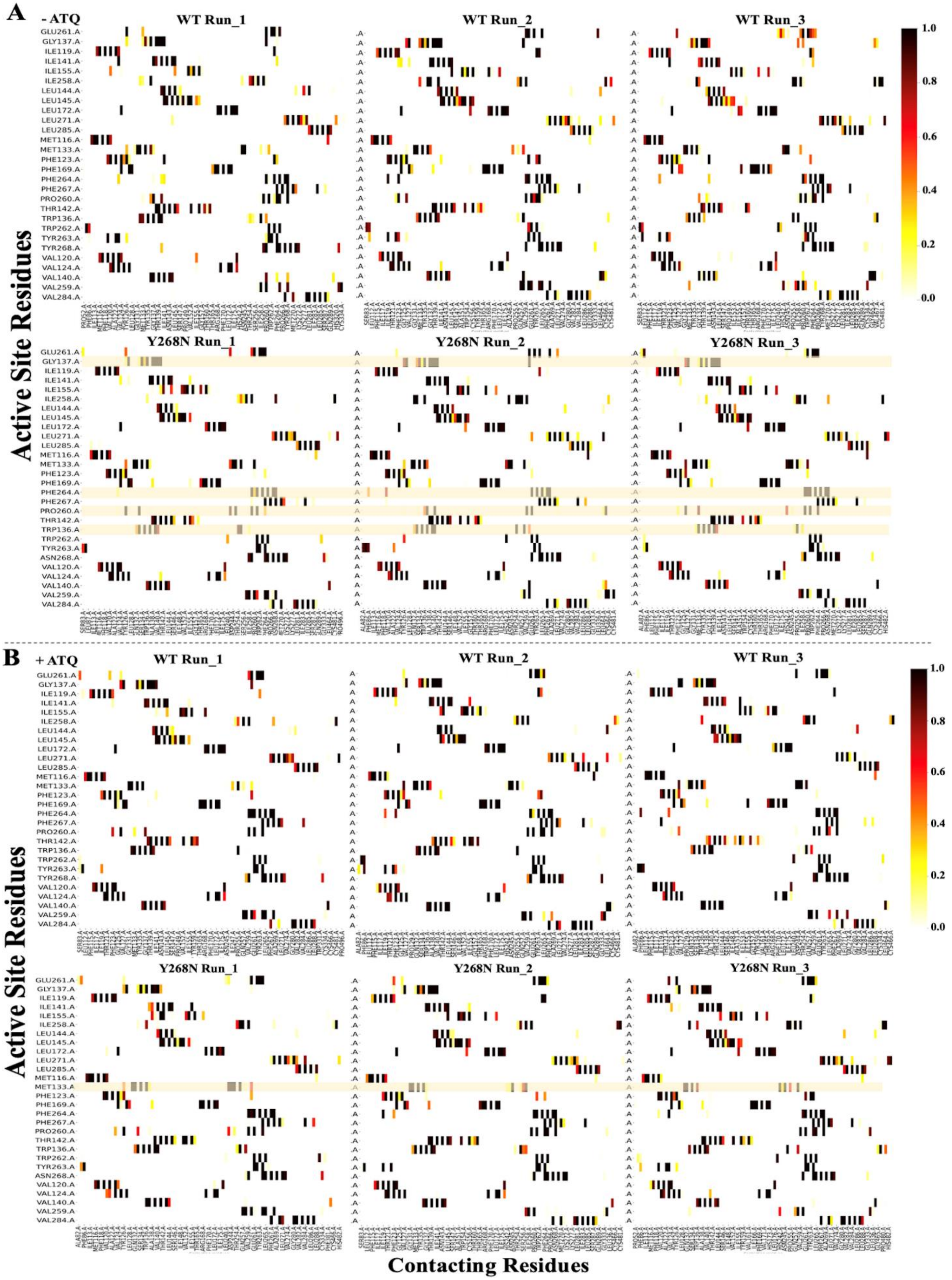

Figure S7. Residue contact heatmap between active site residues (n = 28) and other residue contacts. Comparison of the WT and mutant system Y268N was performed in both Holo and ATQ-bound proteins in their respective triplicate runs in the last 100 ns of MD simulation time. Regions observed to reduce or lose residue contact are shaded in light golden color.

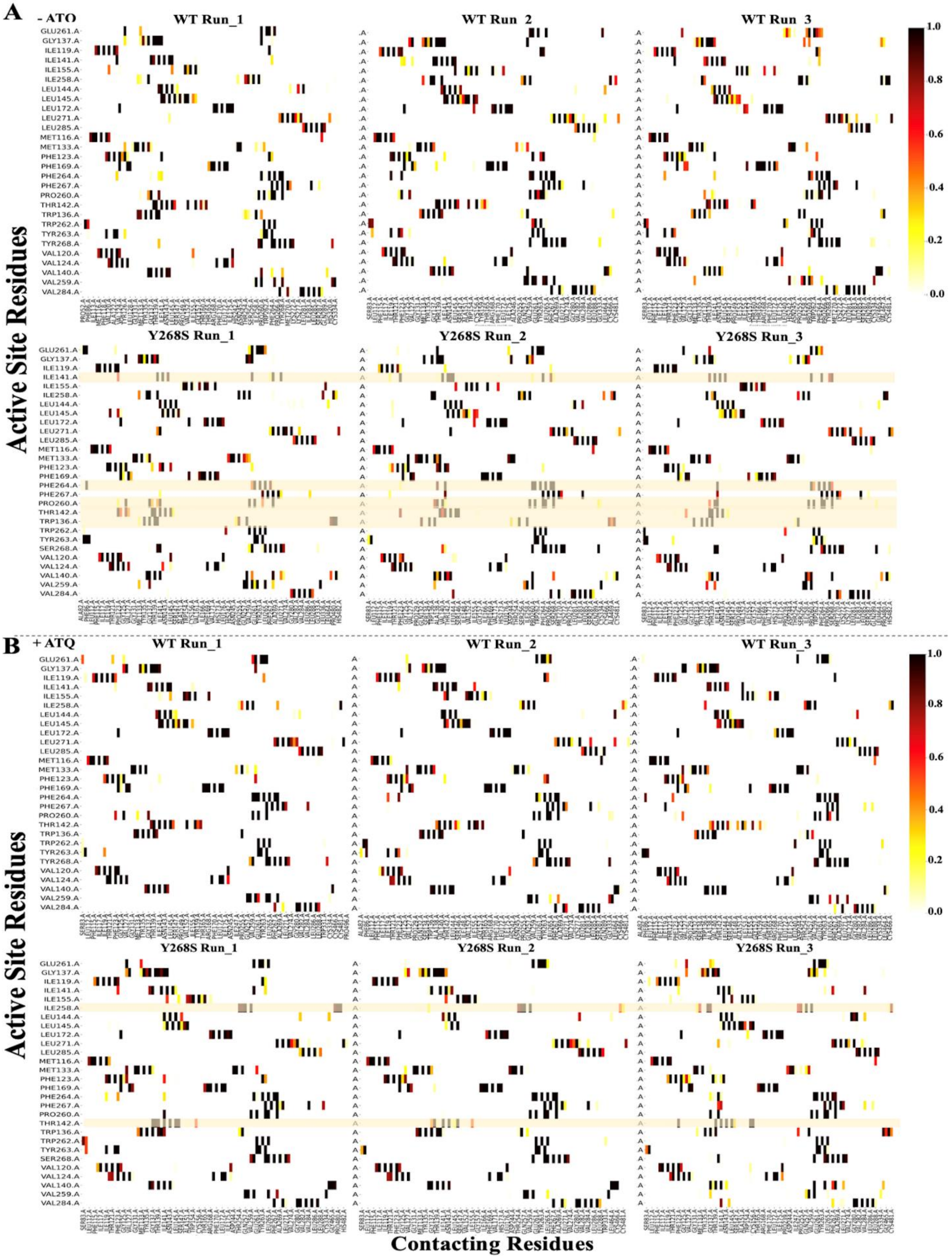

Figure S8. Residue contact heatmap between active site residues (n = 28) and other residue contacts. Comparison of the WT and mutant system Y268S was performed in both Holo and ATQ-bound proteins in their respective triplicate runs in the last 100 ns of MD simulation time. Regions observed to reduce or lose residue contact are shaded in light golden color.

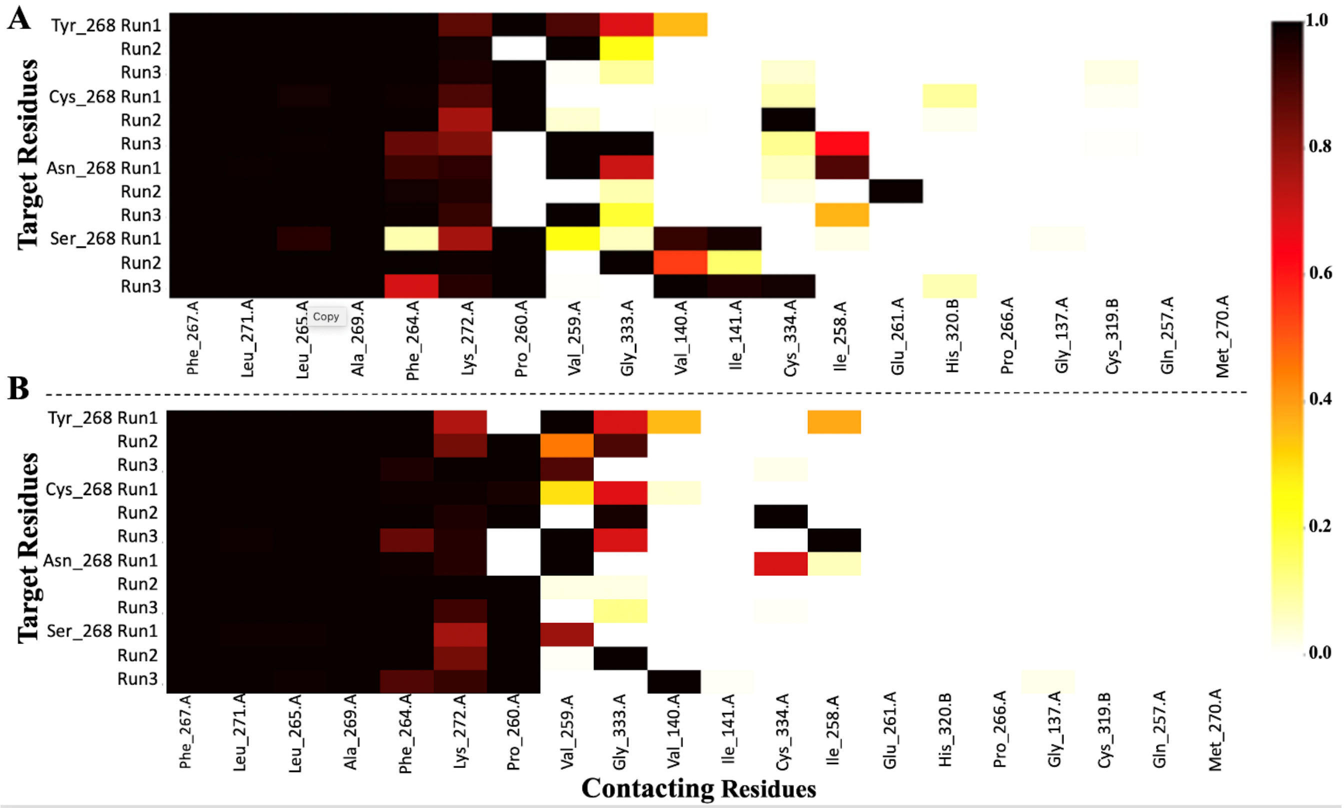

Figure S9. Residue contact heat map of Y268C, Y268N, Y268S mutant systems with reference to wild type yielded during the last 100 ns of MD simulations for all systems in the triplicate runs. **A** PfCytb-ISP holo protein **B** PfCytb-ISP ATQ-bound protein. Individual residue contact maps for WT and all mutant systems in triplicate runs are displayed in Figure S10. Residues ordered by the proportion of contacts across simulation time. Residue names ending with A or B represent chain A and B, respectively.

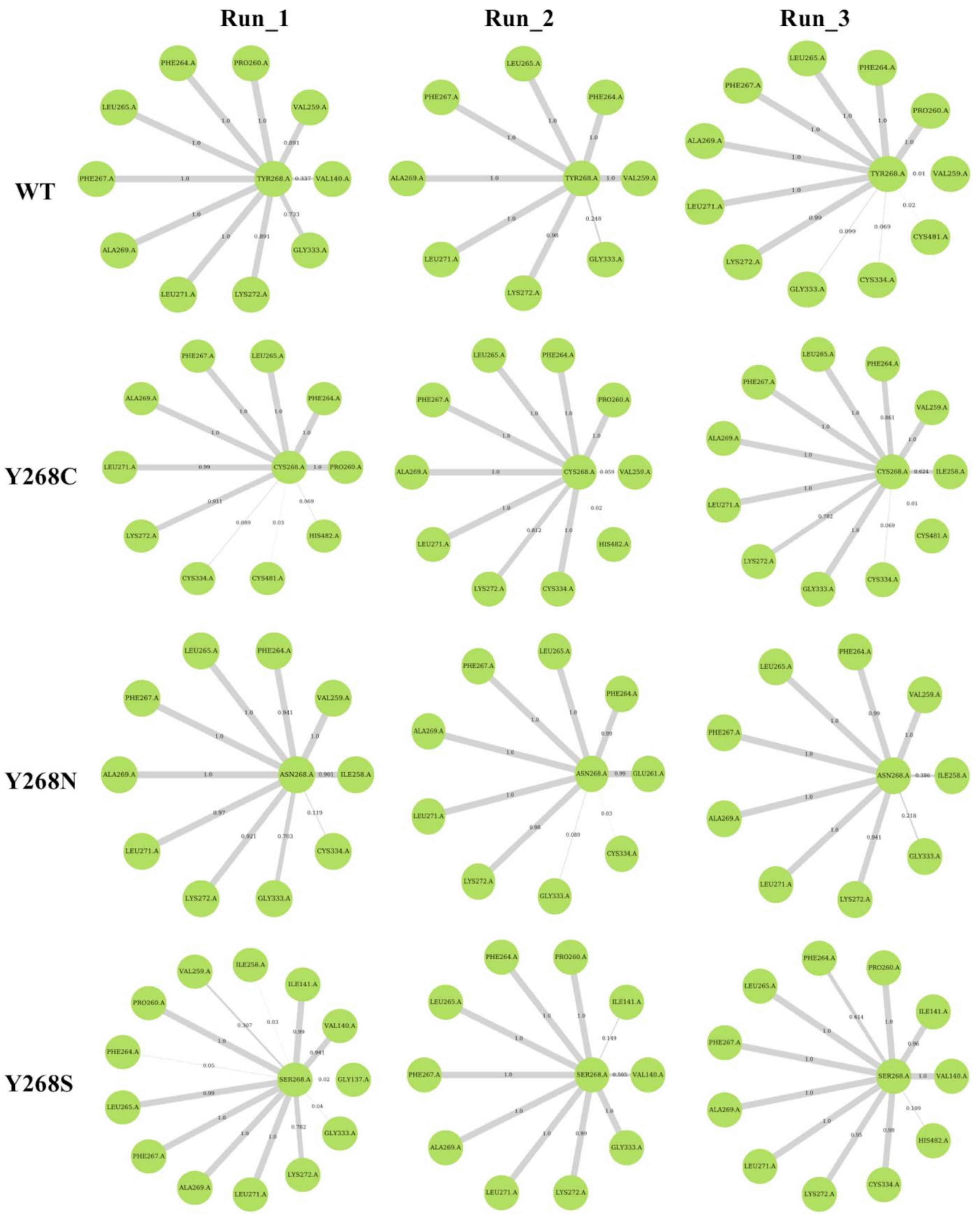

Figure S10. Residue contact map for each of the PfCytb-ISP Holo protein systems (WT, Y268C, Y268N and Y268S) in their respective triplicate runs in the last 100 ns of MD simulation time. Using WT as the reference, contact residues Gly333, Val259, Pro260 and Phe264 were noted to be compromised (reduced/lost) in the presence of mutation.

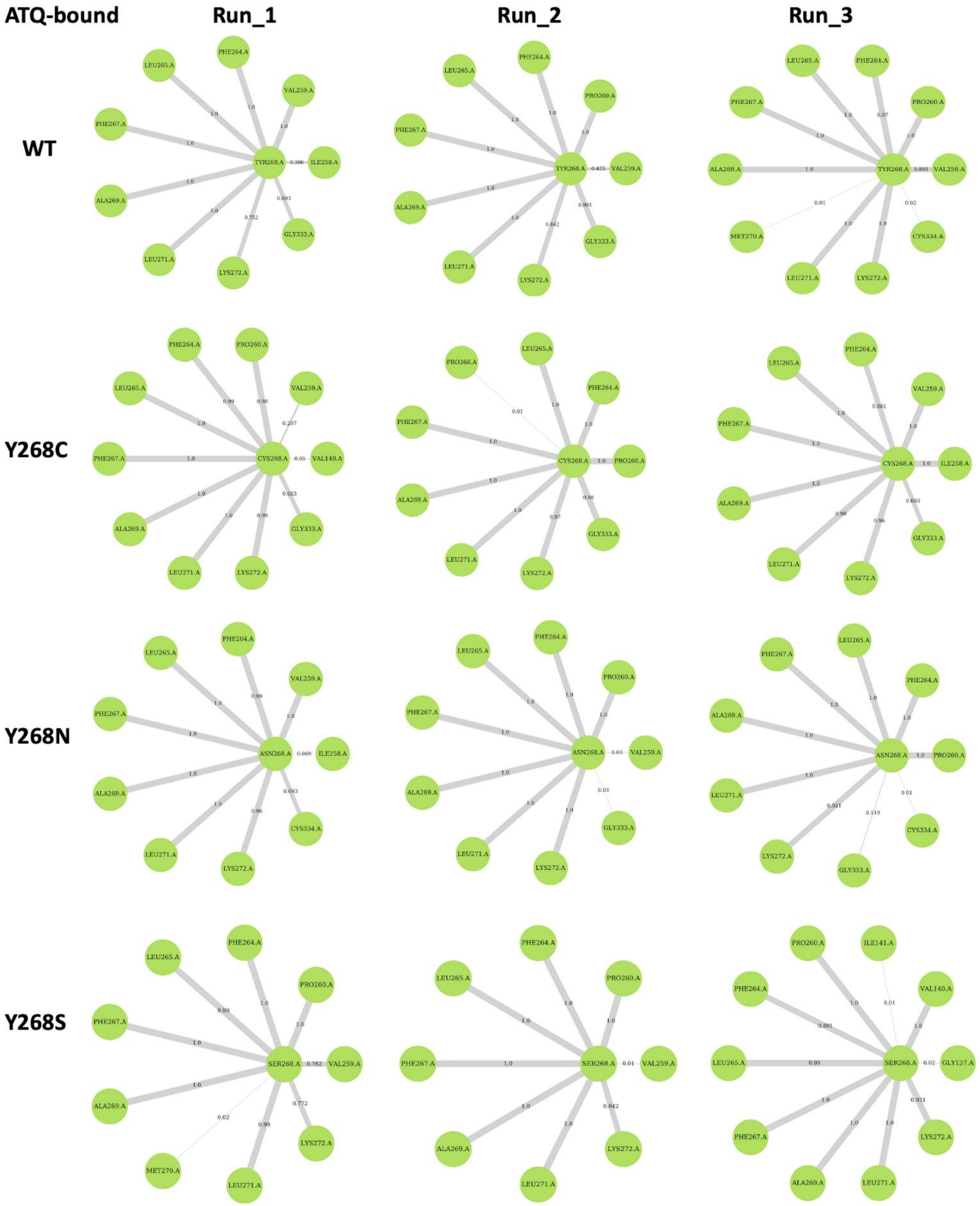

Figure S11. Residue contact map for each of the PfCytb-ISP ATQ-bound protein systems (WT, Y268C, Y268N and Y268S) in their respective triplicate runs in the last 100 ns of MD simulation time. Using WT as the reference, contact residues Gly333, Val259, Pro260 and Phe264 were noted to be compromised (reduced/lost) in the presence of mutation.

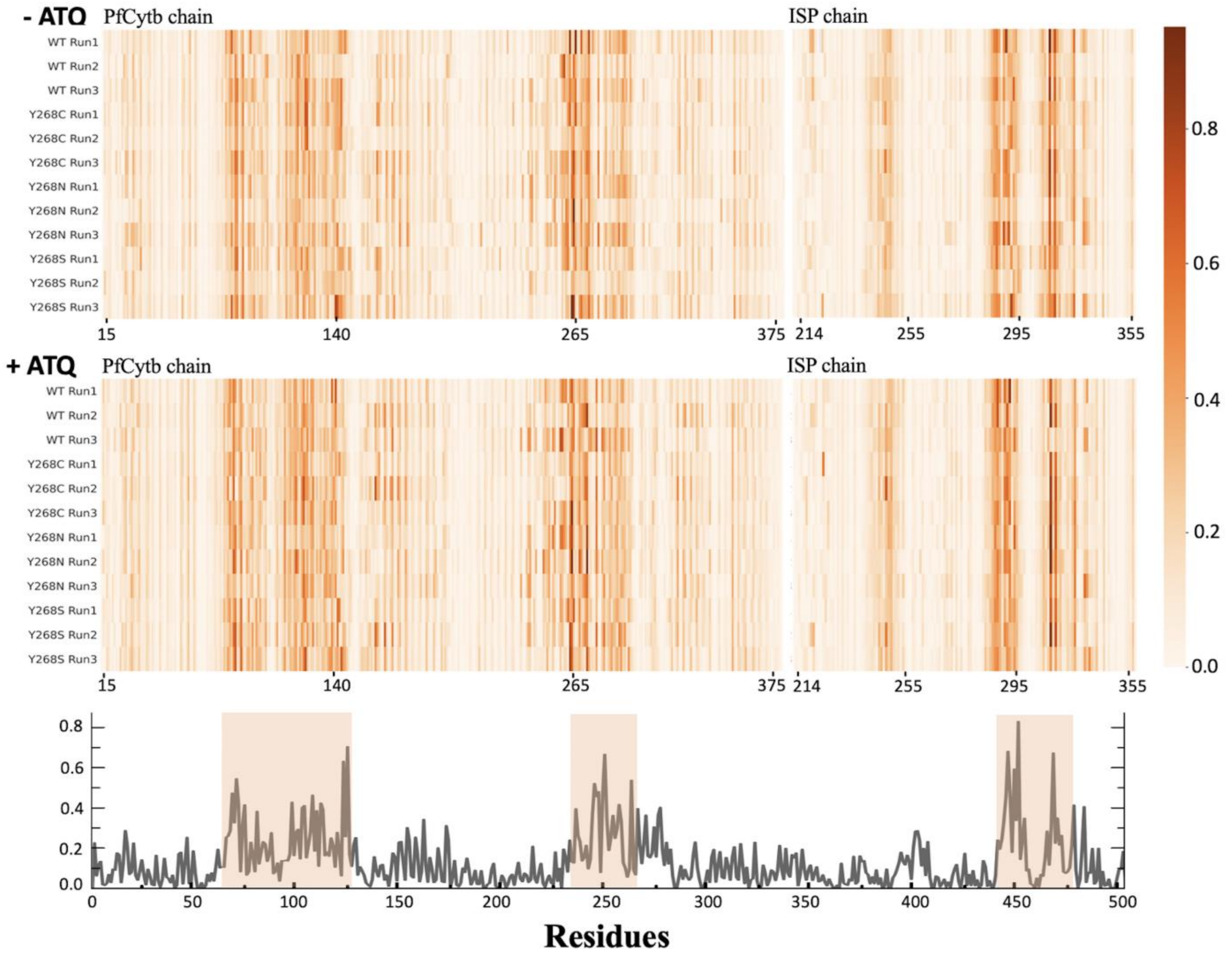

Figure S12. Heat map comparison of BC values in PfCytb-ISP holo and ATQ-bound proteins. High BC values indicate regions of high residue connectivity.

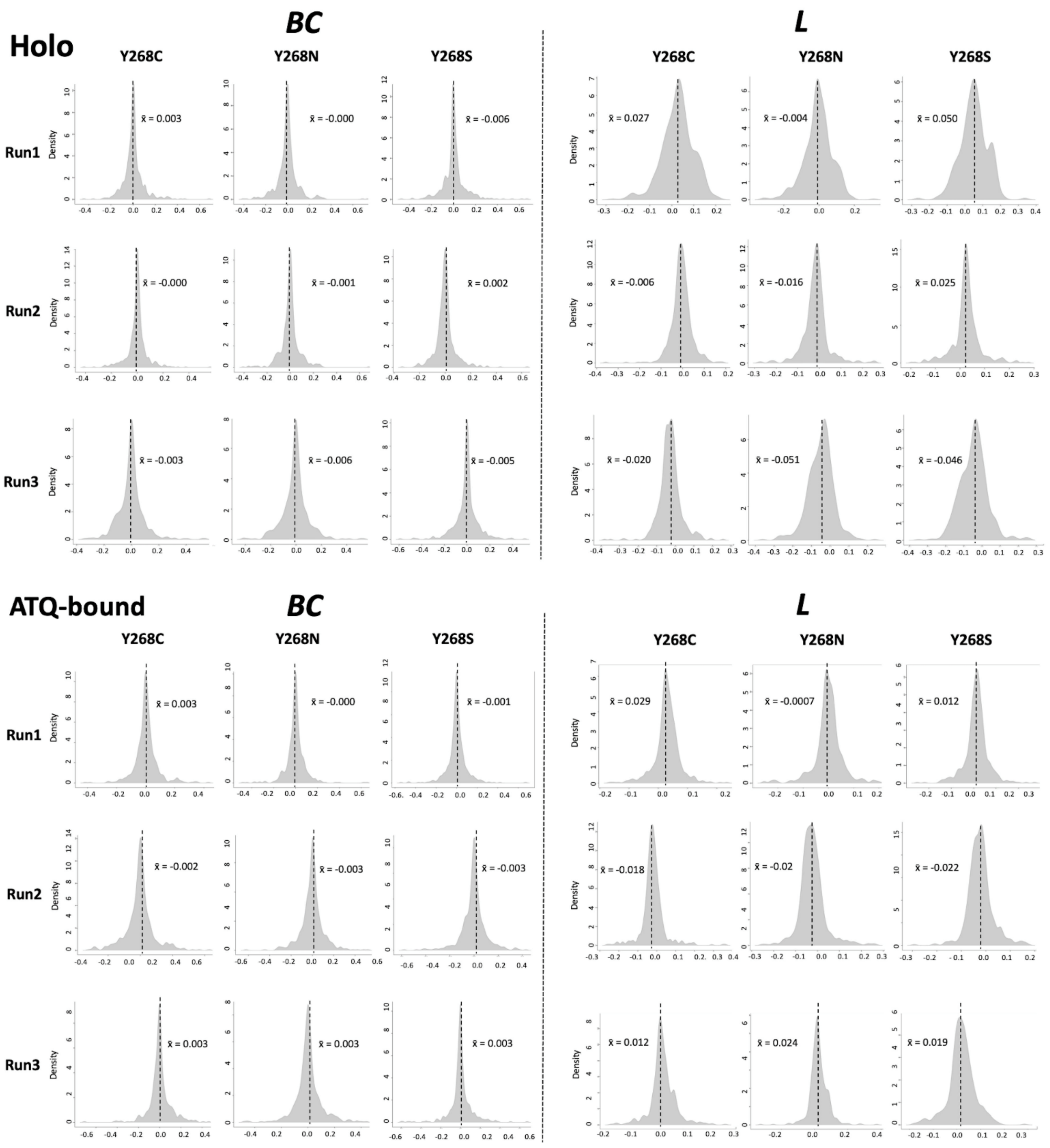

Figure S13. Density plot for delta *BC* and *L* values (N =503) for mutant systems (Y268C, Y268N, and Y268S) in three replicate runs. The plots show that (**A**) *BC* and (**B**) *L* data was normally distributed where the mean is displayed by a dotted line.

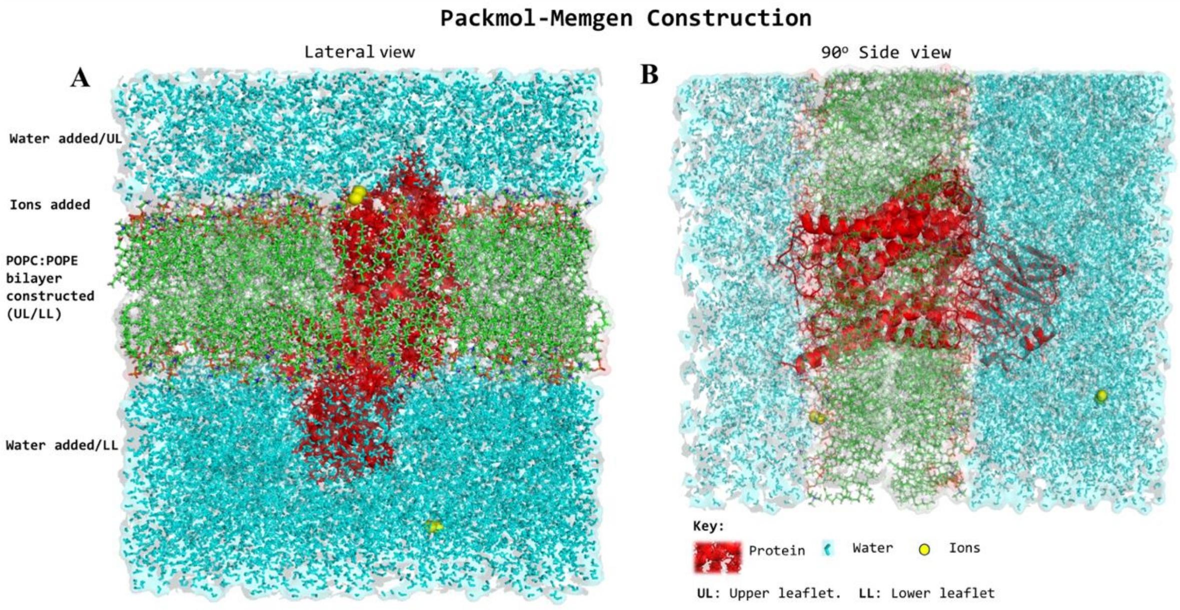

Figure S14. Structural representation of membrane construction with PfCytb-ISP protein. The protein embedded in a POPC:POPE bilayer at a ratio of 1.3:1. (**A**) Shows a lateral view of the system while (**B**) shows the orientation of the protein as seen on the side view.

References

1. Case, D.A.; Betz, R.; Cerutti, D.; Cheatham, T.; Darden, T.; Duke, R.; Giese, T.; Gohlke, H.; Goetz, A.; Homeyer, N.; et al. AMBER 2016 Reference Manual. *Univ. Calif. San Franc.* **2016**, 1–923.
2. Roe, D.R.; Cheatham, T.E. PTRAJ and CPPTRAJ: Software for Processing and Analysis of Molecular Dynamics Trajectory Data. *J Chem Theory Comput* **2013**, *9*, 3084–3095, doi:10.1021/ct400341p.
3. BIOVIA, D.S. Discovery Studio Modeling Environment, Release 2017, San Diego: DassaultSystèmes, 2016. *Available FromAccessed 1 Sept. 2016* **2016**.
4. Frisch, M.; Trucks, G.; Schlegel, H.; Scuseria, G.; Robb, M.; Cheeseman, J.; Scalmani, G.; Barone, V.; Petersson, G.; Nakatsuji, H.; et al. Gaussian 09, Revision A. 02, Gaussian, Inc., Wallingford, CT, 2016. *Chem. Chem. Phys. Accept. Manuscr.*
5. Nielsen, A.; Holder, A. Gauss View 3.0, User's Reference. *Gaussian Inc Pittsburgh PA* **2003**.
6. Abraham, M.; Murtola, T.; Schulz, R.; Páll, S.; Smith, J.; Hess, B.; Lindahl, E. GROMACS: High Performance Molecular Simulations through Multi-Level Parallelism from Laptops to Supercomputers. *SoftwareX* **2015**, *1*, doi:10.1016/j.softx.2015.06.001.
7. Gordon, J.C.; Myers, J.B.; Folta, T.; Shoja, V.; Heath, L.S.; Onufriev, A. H++: A Server for Estimating PKas and Adding Missing Hydrogens to Macromolecules. *Nucleic Acids Res* **2005**, *33*, W368–W371, doi:10.1093/nar/gki464.
8. Zhang, Y. I-TASSER Server for Protein 3D Structure Prediction. *BMC Bioinformatics* **2008**, *9*, 40, doi:10.1186/1471-2105-9-40.
9. Roy, A.; Kucukural, A.; Zhang, Y. I-TASSER: A Unified Platform for Automated Protein Structure and Function Prediction. *Nat. Protoc.* **2010**, *5*, 725–738, doi:10.1038/nprot.2010.5.
10. Yang, J.; Yan, R.; Roy, A.; Xu, D.; Poisson, J.; Zhang, Y. The I-TASSER Suite: Protein Structure and Function Prediction. *Nat. Methods* **2015**, *12*, 7–8, doi:10.1038/nmeth.3213.
11. Schrödinger *Schrödinger Release 2019–3, Maestro*; New York, 2019;
12. Brown, D.K.; Penkler, D.L.; Sheik Amamuddy, O.; Ross, C.; Atilgan, A.R.; Atilgan, C.; Tastan Bishop, Ö. MD-TASK: A Software Suite for Analyzing Molecular Dynamics Trajectories. *Bioinformatics* **2017**, *33*, 2768–2771, doi:10.1093/bioinformatics/btx349.
13. Li, P.; Merz, K.M. MCPB.Py: A Python Based Metal Center Parameter Builder. *J Chem Inf Model* **2016**, *56*, 599–604, doi:10.1021/acs.jcim.5b00674.
14. Schott-Verdugo, S.; Gohlke, H. PACKMOL-Memgen: A Simple-to-Use, Generalized Workflow for Membrane-Protein–Lipid-Bilayer System Building. *J. Chem. Inf. Model.* **2019**, *59*, 2522–2528, doi:10.1021/acs.jcim.9b00269.
15. Laskowski, R.; MacArthur, M.; Moss, D.; Thornton, J. PROCHECK: A Program to Check the Stereochemical Quality of Protein Structures. *J. Appl. Crystallogr.* **1993**, *26*, 283–291, doi:10.1107/s0021889892009944.
16. DeLano, W.L.; others Pymol: An Open-Source Molecular Graphics Tool. *CCP4 Newsl. Protein Crystallogr.* **2002**, *40*, 82–92.

17. Lovell, S.C.; Davis, I.W.; Arendall, W.B.; de Bakker, P.I.W.; Word, J.M.; Prisant, M.G.; Richardson, J.S.; Richardson, D.C. Structure Validation by Calpha Geometry: Phi,Psi and Cbeta Deviation. *Proteins* **2003**, *50*, 437–450, doi:10.1002/prot.10286.
18. Humphrey, W.; Dalke, A.; Schulten, K. VMD: Visual Molecular Dynamics. *J. Mol. Graph.* **1996**, *14*, 33–8, 27–8, doi:10.1016/0263-7855(96)00018-5.
